# Multi-omics analysis reveals the influence of genetic and environmental risk factors on developing gut microbiota in infants at risk of celiac disease

**DOI:** 10.1186/s40168-020-00906-w

**Published:** 2020-09-11

**Authors:** Maureen M. Leonard, Hiren Karathia, Meritxell Pujolassos, Jacopo Troisi, Francesco Valitutti, Poorani Subramanian, Stephanie Camhi, Victoria Kenyon, Angelo Colucci, Gloria Serena, Salvatore Cucchiara, Monica Montuori, Basilio Malamisura, Ruggiero Francavilla, Luca Elli, Brian Fanelli, Rita Colwell, Nur Hasan, Ali R. Zomorrodi, Alessio Fasano, the CD-GEMM Team, Pasqua Piemontese, Angela Calvi, Mariella Baldassarre, Lorenzo Norsa, Chiara Maria Trovato, Celeste Lidia Raguseo, Tiziana Passaro, Paola Roggero, Marco Crocco, Annalisa Morelli, Michela Perrone, Marcello Chieppa, Giovanni Scala, Maria Elena Lionetti, Carlo Catassi, Adelaide Serretiello, Corrado Vecchi, Gemma Castillejo de Villsante

**Affiliations:** 1 Division of Pediatric Gastroenterology and Nutrition, MassGeneral Hospital for Children, Harvard Medical School, Boston, MA USA; 2 Mucosal Immunology and Biology Research Center, MassGeneral Hospital for Children, Boston, USA; 3 Harvard Medical School, Boston, MA USA; 4 Celiac Research Program, Harvard Medical School, Boston, MA USA; 5 CosmosID Inc., Rockville, MD USA; 6 Theoreo srl, University of Salerno, Montecorvino Pugliano, Italy; 7 Department of Medicine, Surgery and Dentistry, Scuola Medica Salernitana, University of Salerno, Salerno, Italy; 8 European Biomedical Research Institute of Salerno (EBRIS), Via S. De Renzi, 50, 84125 Salerno, Italy; 9 Pediatric Unit, Maternal and Child Health Department, AOU San Giovanni di Dio e Ruggi d’Aragona, Salerno, Italy; 10 Pediatric Gastroenterology, Sapienza University of Rome, Rome, Italy; 11 Pediatric Unit, Maternal and Child Health Department, AOU San Giovanni di Dio e Ruggi d’Aragona, Pole of Cava de’ Tirreni, Salerno, Italy; 12 Pediatric Gastroenterology, University of Bari, Bari, Italy; 13 Center for Prevention and Diagnosis of Celiac Disease Fondazione IRCCS Ca’ Granda Ospedale Maggiore Policlinico, Milan, Italy; 14 Center for Bioinformatics and Computational Biology, University of Maryland, College Park, MD USA

**Keywords:** Microbiota, Celiac disease, Multi-omics analysis, gut microbiome

## Abstract

**Background:**

Celiac disease (CD) is an autoimmune digestive disorder that occurs in genetically susceptible individuals in response to ingesting gluten, a protein found in wheat, rye, and barley. Research shows that genetic predisposition and exposure to gluten are necessary but not sufficient to trigger the development of CD. This suggests that exposure to other environmental stimuli early in life, e.g., cesarean section delivery and exposure to antibiotics or formula feeding, may also play a key role in CD pathogenesis through yet unknown mechanisms. Here, we use multi-omics analysis to investigate how genetic and early environmental risk factors alter the development of the gut microbiota in infants at risk of CD.

**Results:**

Toward this end, we selected 31 infants from a large-scale prospective birth cohort study of infants with a first-degree relative with CD. We then performed rigorous multivariate association, cross-sectional, and longitudinal analyses using metagenomic and metabolomic data collected at birth, 3 months and 6 months of age to explore the impact of genetic predisposition and environmental risk factors on the gut microbiota composition, function, and metabolome prior to the introduction of trigger (gluten). These analyses revealed several microbial species, functional pathways, and metabolites that are associated with each genetic and environmental risk factor or that are differentially abundant between environmentally exposed and non-exposed infants or between time points. Among our significant findings, we found that cesarean section delivery is associated with a decreased abundance of *Bacteroides vulgatus* and *Bacteroides dorei* and of folate biosynthesis pathway and with an increased abundance of hydroxyphenylacetic acid, alterations that are implicated in immune system dysfunction and inflammatory conditions. Additionally, longitudinal analysis revealed that, in infants not exposed to any environmental risk factor, the abundances of *Bacteroides uniformis* and of metabolite 3-3-hydroxyphenylproprionic acid increase over time, while those for lipoic acid and methane metabolism pathways decrease, patterns that are linked to beneficial immunomodulatory and anti-inflammatory effects.

**Conclusions:**

Overall, our study provides unprecedented insights into major taxonomic and functional shifts in the developing gut microbiota of infants at risk of CD linking genetic and environmental risk factors to detrimental immunomodulatory and inflammatory effects.

Video Abstract

## Background

Celiac disease (CD) is an autoimmune enteropathy, which affects three million Americans and 1% of the population worldwide [1]. CD occurs in genetically predisposed individuals that have specific variants of the human leukocyte antigen (HLA) DQ2 and DQ8 genes in response to ingesting gluten, a protein found in wheat, rye, and barley [2]. Notably, CD is the only autoimmune disorder for which the environmental trigger (ingestion of gluten) is known [3]. Given that the timing of exposure to gluten and the dose of gluten ingested can be carefully monitored, and since gluten removal results in the resolution of symptoms and enteropathy for most patients [4–8], CD can serve as a tunable model of chronic immune-based disorders [9]. This allows for insights into its pathogenesis to be applied not only to individuals with CD but those with other autoimmune diseases as well.

Globally, the incidence of autoimmune diseases including CD is expected to triple by 2050 [10, 11], yet the genes associated with CD (HLA DQ2) and DQ8, and the trigger (gluten) have not changed. Research shows that more than 30% of the population carry the predisposing gene(s) and are exposed to the trigger, yet only 2–3% of these individuals develop CD in their lifetime thus suggesting a critical role for environmental factors [12]. Mode of delivery, infant feeding type, timing of gluten introduction into the diet, occurrence of viral infections, and early exposure to antibiotics are just a few of the many environmental factors suggested to influence the development of chronic inflammatory diseases such as CD [13]. When evaluating these factors independently, case-control studies and meta-analyses have found that cesarean section delivery [14, 15], lack of breast-feeding [16, 17], timing of gluten introduction [17, 18], and exposure to antibiotics [19] increase the risk of developing CD. However, two independent double blind placebo controlled prospective studies in Europe involving infants with compatible HLA genetics and a first-degree relative with CD (who are therefore at high risk of developing CD) found that vaginal delivery, breast-feeding, and timing of gluten introduction were not protective against developing CD [20, 21].

Accumulating evidence suggests that the gut microbiota may be involved in several immune-based disorders [13] such as inflammatory bowel disease (IBD) [22], type 1 diabetes (T1D) [23], and multiple sclerosis [24]. A limited number of studies have also started to explore the link between the gut microbiota and CD development [25–30]. Initial studies focused on the contribution of HLA genetics to the developing microbiota. In particular, two studies analyzed exclusively breastmilk-fed infants up to 4 months of age with a first-degree relative with CD and found that *Bacteroides*-*Prevotella* group [25], *Firmicutes*, *Proteobacteria*, and *Bifidobacterium* [26] were more abundant in infants at high genetic risk for CD (those with two copies of HLA DQ2). Additionally, in a preliminary prospective study, we used 16S rRNA amplicon sequencing to examine the microbiota from 16 infants with a first-degree relative with CD and with a compatible HLA type and found a lower abundance of *Bacteroides* and a higher abundance of *Firmicutes* in these subjects compared to controls [27]. Other studies of the gut microbiota and CD have assessed changes, within 1 year of age, in the microbiota composition of individuals who later developed CD compared to controls [29, 30]. For example, Olivares et al. [29] identified increases in the abundances of *Firmicutes*, *Enterococcaceae*, and *Peptostreptococcaceae* in controls from 4 to 6 months but no differences over time were observed in cases [29]. While the link between environmental factors and alterations in the gut microbiota of at-risk subjects has been recently explored for a number of chronic immune-based disorders [31, 32], studies addressing this question for CD are scarce [28]. The only study in this direction is the work of Pozo-Rubio et al. [28], where they found associations between a limited number of pre-selected fecal microbial taxa in subjects at risk of CD and delivery mode, infant feeding type, antibiotic exposure, and rotavirus vaccine administration [28].

While these studies have provided valuable insights into the development of the gut microbiota early in life in subjects at risk of CD, solid food has already been introduced into the infants’ diet in many of these studies without accounting for its impact on the microbiota. In addition, to the best of our knowledge, no microbiome-wide study of the effect of environmental risk factors for CD currently exists. More importantly, existing studies are primarily based on 16S rRNA amplicon sequencing, which is not capable of fully addressing how the functional characterization of the microbiota will affect CD onset. To mitigate these limitations, here, we utilize a large-scale prospective cohort study called the Celiac Disease Genomic, Environmental, Microbiome and Metabolome study (CDGEMM) [33], where we have been following over 400 infants with a first-degree relative with CD who are thus at a high risk of developing CD. In this study, we present multivariate association as well as inter-subject and intra-subject analyses using metagenomic and metabolomic data collected over the first 6 months after birth to investigate the impact of both genetic and environmental risk factors on the development of the gut microbiota of infants at risk of CD prior to the introduction of solid foods.

## Results

**Fig. 1:**
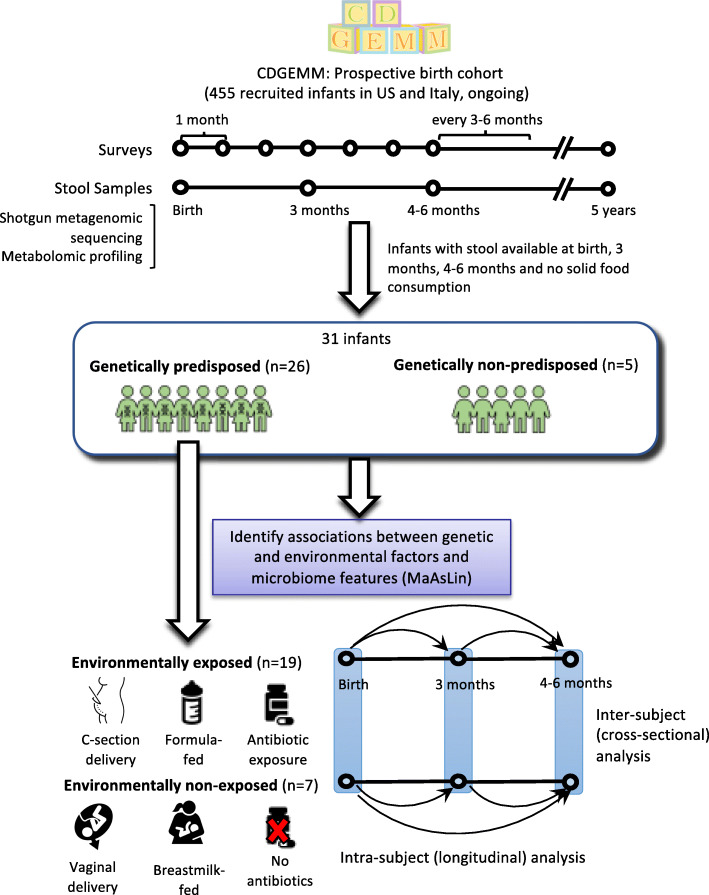
Schematic representing the sample selection and study design. We selected 31 infants from the CDGEMM study [33] with fecal samples available at enrollment, 3 months, and 4–6 months after birth. The sample underwent metagenomic and metabolomic profiling and was next analyzed to identify associations between genetic and environmental risk factors and inter-subject and intra-subject variations

We selected 31 children recruited into the CDGEMM cohort for whom stool samples were available at birth, 3 months, and 4–6 months for this study (see Fig. 1, Table 1, and Additional File 1 for more detailed metadata). None of these infants consumed solid foods before 6 months, which makes them ideal for studying the effect of genetic and environmental risk factors on the gut microbiota in the absence of gluten as a confounder. Twenty-six of these infants were genetically susceptible to developing CD out of which 19 were either heterozygous for DQ2 or DQ8 or carried both DQ2 and DQ8 (referred to as “standard genetic risk” hereafter) and seven were homozygous for DQ2 (referred to as “high genetic risk” hereafter). Additionally, 19 infants who were genetically predisposed to CD and that have been exposed to at least one environmental risk factor are referred to as “environmentally exposed” infants throughout the rest of manuscript. The environmental factors that we considered in this study include delivery model, antibiotic exposure and infant feeding type. Therefore, environmentally exposed infants are the ones who were born via cesarean section or were exposed to antibiotics at or during birth (i.e., antibiotics administered to the mother during delivery) or were not exclusively breastmilk-fed (i.e., formula-fed or both formula- and breastmilk-fed). The choice of these environmental risk factors and their grouping is clinically relevant since cesarean section delivery is often associated with antibiotic administration at birth and formula feeding due to delayed breastmilk production. Seven infants who were genetically susceptible and that were not exposed to any of these environmental risk factors, i.e., those born vaginally and not exposed to antibiotics at or during delivery and exclusively breastmilk-fed, are referred to as “environmentally non-exposed” hereafter (see Fig. 1).

**Table 1:** Study cohort metadata and genotype. This study cohort was extracted from the larger CDGEMM prospective longitudinal birth cohort study [33]

	USA (***n*** = 18)	Italy (***n*** = 13)	Total (***n*** = 31)
Gender (%)			
Male	11 (61.1)	7 (53.8)	18 (58.0)
Female	7 (38.9)	6 (46.2)	13 (42.0)
Mode of delivery (%)			
Vaginal	11 (61.1)	7 (53.8)	18 (58.0)
C-section	7 (38.9)	6 (46.2)	13 (42.0)
Feeding type (4–6 months of age) (%)			
Breastmilk only	12 (66.7)	4 (30.7)	16 (51.6)
Formula only	5 (27.8)	6 (46.2)	11 (35.5)
Both	1 (5.5)	3 (23.1)	4 (12.9)
Antibiotic exposure (%)			
At delivery (mother)	7 (38.9)	2 (15.4)	9 (29.0)
At birth (infant)	2 (11.1)	2 (15.4)	4 (12.9)
Before 6 months of age (infant)	0 (0.0)	4 (30.8)	4 (12.9)
Genotype (%)			
DQ2 homozygous	6 (33.3)	1 (7.7)	7 (22.6)
DQ2 heterozygous	6 (33.3)	6 (46.2)	12 (38.7)
DQ2/DQ8	3 (16.7)	2 (15.4)	5 (16.1)
DQ8	1 (5.5)	1 (7.7)	2 (6.5)
Negative	2 (11.1)	3 (23.1)	5 (16.1)
Relative with CD			
Mother	15 (83.3)	7 (53.8)	22 (70.9)
Father	1 (5.5)	1 (7.7)	2 (6.5)
Sibling	2 (11.1)	5 (38.4)	7 (22.6)

Collected stool samples underwent shotgun metagenomic sequencing and metabolomic profiling. We analyzed metagenomic sequencing reads (see the “Methods” section) to profile microbial taxa at species-level resolution (see Additional File 2 see also Additional File 3 for the taxonomic composition of each sample at the genus and family levels) and functional pathways encoded by metagenomes (see Additional File 4). While we identified non-bacterial species (fungi, viruses, protists) in our taxonomic profiling, in this paper, we focus only on the bacterial species.

Additionally, stool samples underwent metabolomic profiling and were analyzed to identify metabolites present in each stool sample (see Additional File 5). The identified microbial taxa, functional pathways, and metabolites were then analyzed to explore how genetic and environmental risk factors influence the development of the gut microbiota as outlined below.

### Associations between genetic and environmental risk factors and microbiota features

**Fig. 2:**
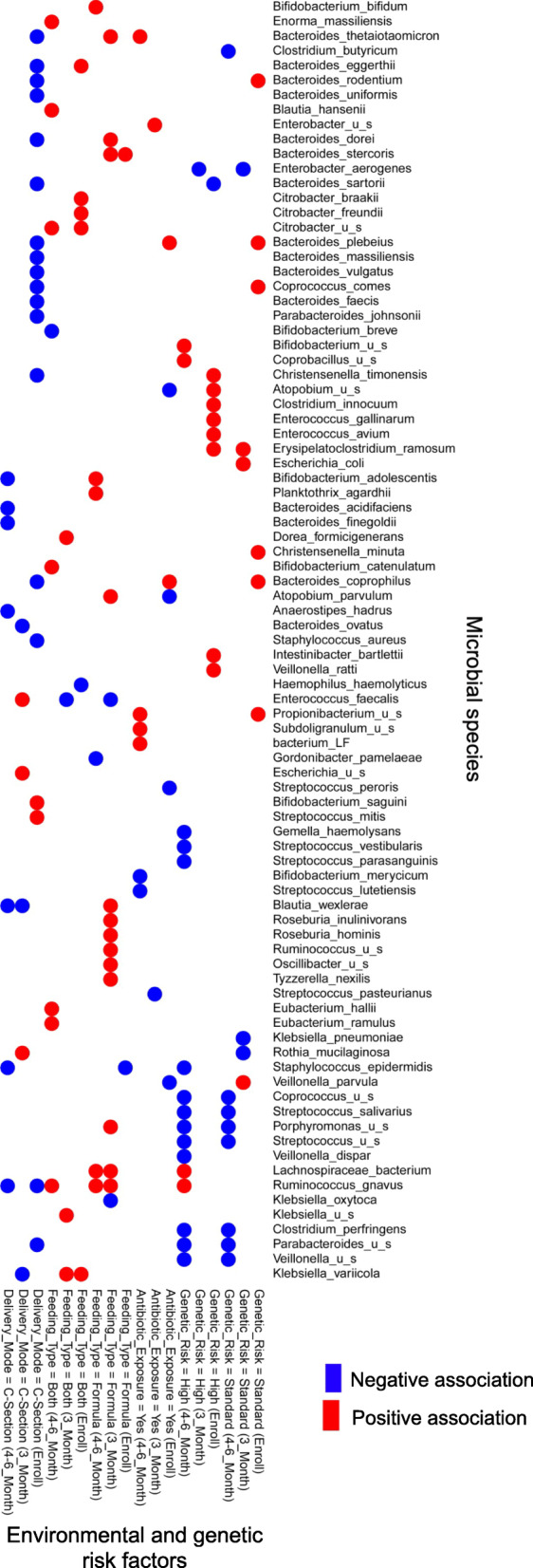
Analysis of associations between genetic and environmental risk factors and microbial species. We used MaAsLin [22], a widely used multivariate statistical framework, to identify statistically significant associations between each genetic and environmental risk factor and microbial species (*p* value < 0.01), No genetic risk, vaginal delivery, exclusive breastmilk feeding, and no exposure to antibiotics were taken as reference for genetic risk, delivery mode, feeding type, and antibiotic exposure, respectively. Microbial species were clustered based on Euclidean distance. Here, “u_s” denotes and unspecified species

**Fig. 3:**
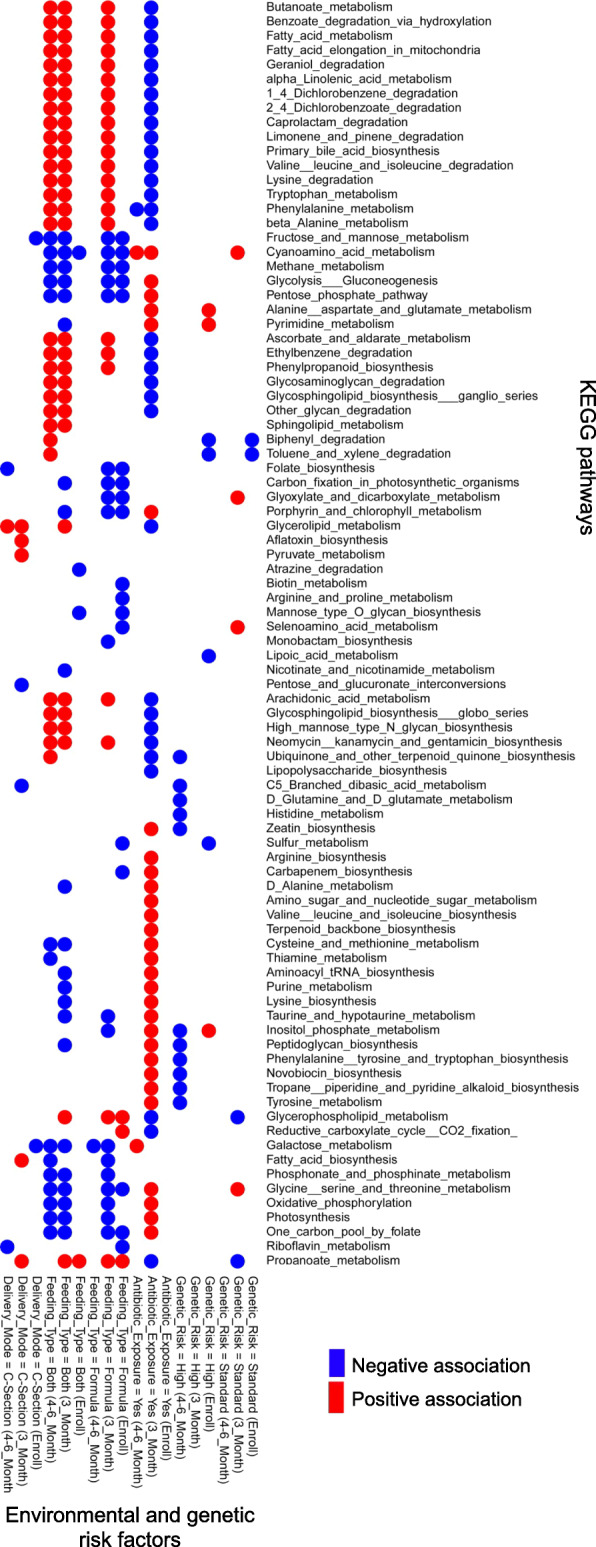
Analysis of associations between genetic and environmental risk factors and functional pathways. We used MaAsLin [22] to identify statistically significant associations between each genetic and environmental risk factor and functional pathways (*p* value < 0.01), Pathways were clustered based on Euclidean distance. Additional File 8 for grouping of these pathways based on KEGG categorizations

**Fig. 4:**
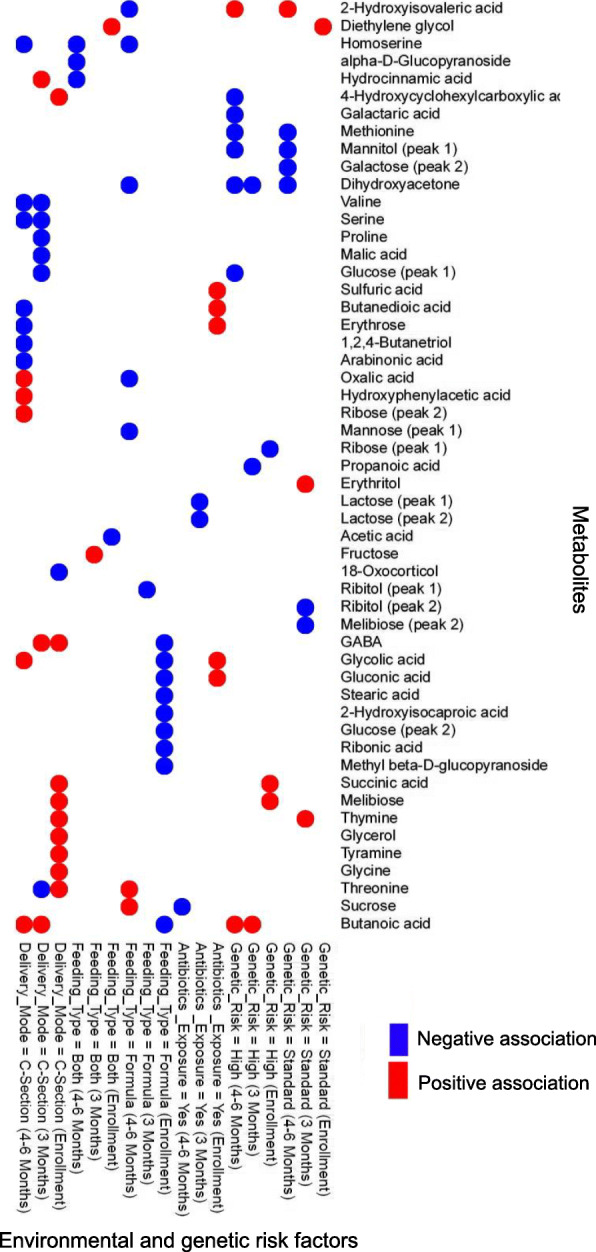
Analysis of associations between genetic and environmental risk factors and metabolites. We used MaAsLin [22] to identify statistically significant associations between each genetic and environmental risk factor and metabolites (*p* value < 0.01). Metabolites were clustered based on Euclidean distance

We used the MaAslin procedure [22] to investigate how various microbiome features including microbial species, functional pathways, and metabolites at each time point are associated with genetic risk for developing CD and three key environmental risk factors including mode of delivery, exposure to antibiotics, and infant feeding type (see Figs. 2, 3, and 4).

#### Genetic risk

We found that both high and standard genetic risk to develop CD are associated with a decreased abundance of several species of *Streptococcus* and *Coprococcus* at 4–6 months of age compared to those lacking genetic compatibility (Fig. 2; *p* value < 0.05). Notably, a decreased abundance of *Coprococcus* has been previously reported in the gut of individuals who carry a genetic risk to develop autoimmune conditions including CD [34]. Standard and high genetic risk for developing CD are also associated with an increased abundance of *Bacteroides* and *Enterococcus* species, respectively, at enrollment compared to no genetic risk. These observations are in agreement with previous studies [25, 26]; however, an association between genetic risk and increased abundance of *Bifidobacterium* or *Proteobacteria*, which were reported before [25, 26] was not observed here. Among other significant associations, we found a decreased abundance of *Veillonella*, *Parabacteroides*, and *Clostridium perfringens* at 4–6 months after birth in infants with high and standard genetic compatibility. This observation is contrary to case-control studies that report an increased abundance of these microbes in autoimmune conditions such as autoimmune liver disease [35], Bechet’s disease [36], and neuromyelitis optica [37].

In addition to association with microbial species, we found that a high genetic risk of developing CD is associated with a decreased abundance of a number of functional pathways at 4–6 months of age (Fig. 3; *p* value < 0.05). These pathways include amino acid metabolism, biosynthesis of secondary metabolites, and metabolism of cofactors including ubiquinone and other terpenoid-quinone biosynthesis. Furthermore, we identified an association between high genetic risk and a number of metabolites, e.g., an increased abundance of butanoic acid and a decreased abundance of dihydroxyacteone at 3 and 4–6 months of age (Fig. 4, *p* value < 0.05).

#### Mode of delivery

We found that cesarean section delivery is associated with a decreased abundance of several species of *Bacteroides* and *Parabacteroides* at all time points and with an increased abundance of *Enterococcus faecalis* (at 3 months after birth) compared to vaginal delivery (Fig. 2; *p* value < 0.05) in agreement with previous work [23, 38–40]. For example, we found associations between cesarean section delivery and a decreased abundance of beneficial species *Bacteroides vulgatus* and *Bacteroides dorei*. An increased abundance of these species has been reported to lead to a decreased gut microbial production of lipopolysaccharide, which will improve immune function through mechanisms such as major histocompatibility production and T cell activation, among others [41]. Analysis of pathways shows also an association between cesarean section delivery and decreased riboflavin metabolism and folate biosynthesis at 4–6 months after birth and an increase in the abundance of glycerolipid metabolism at 3 and 4–6 months (Fig. 3; *p* value < 0.05). Of note, defects in folate biosynthesis have been linked to an impaired immune response to viral infections and reduced natural killer cell response possibly contributing to T1D onset [42]. Finally, metabolites analysis unveiled an association between cesarean section delivery and an increase in the abundance of a number of metabolites such as butanoic acid (at 3 and 4–6 months), glycolic acid, oxalic acid, and hydroxyphenlacetic acid (at 4–6 months) and a decrease in that of valine, serine, and arabinoic acid among others (at 4–6 months) (Fig. 4, *p* value < 0.05). An increased abundance of hydroxyphenlacetic acid in the serum has been associated with ulcerative colitis in a previous study [43]; however, no clear links between the level of metabolites in the gut and those in the serum have been established yet. Additionally, serine, which is decreased in cesarean section delivery, has been reported to be required for effector T cell expansion and thus for modulating the adaptive immune response [44].

#### Infant feeding type

We examined three infant feeding types in this study including exclusive breastmilk feeding, exclusive formula feeding and both breastmilk and formula feeding, the last two of which were considered environmental risk factors. Previous work shows an association between infant feeding type and distinct species of *Bifidobacterium* [23, 45]. Consistent with these reports, we observed that exposure to both breastmilk and formula is associated with a decreased abundance *of Bifidobacterium breve* (at 4–6 months) while exclusive formula feeding is associated with an increased abundance of *Bifidobacterium adolescentis* compared to exclusive breastmilk feeding (Fig. 2; *p* value < 0.05). We also found that exclusive formula feeding is associated with a decreased abundance of *Staphylococcus epidermis* (at enrollment) consistent with previous work [46], and with an increased abundance of *Ruminococcus gnavus* and *Lachnospiraceae bacterium* (at 3 and 4–6 months), which have been linked to allergic disease [47], diabetes [48], and colonic inflammation [49]. Pathway analysis shows that exposure to formula only or both breastmilk and formula is associated with an increased abundance of pathways for lipids, amino acids and terpendoids metabolism, and xenobiotic degradation, and with a decreased abundance of pathways for carbohydrate and energy metabolism (Fig. 3; *p* value < 0.05). Additionally, metabolomic analysis uncovered an association between both breastmilk and formula feeding with a decreased abundance of homoserine, alpha-d-glucopyranoside, and hydrocinnamic acid (at 4–6 months) (Fig. 4; *p* value < 0.05). Exclusive formula feeding is also associated with an increase in sucrose and threonine and a decrease in oxalic acid and dihydroxyacetone abundances, among others (at 4–6 months).

#### Antibiotic use

We found an association between antibiotic exposure (as an environmental risk factor) and an increased abundance of *Bacteroides thetaiotaomicron* (at 4–6 months of age) (Fig. 2; *p* value < 0.05). This is corroborated with previous work suggesting that this species, which is an important metabolizer of polysaccharides, increases in abundance in response to amoxicillin exposure [50]. Other identified associations for antibiotic exposure not previously reported include an increased *Propionibacterium*, *Subdoligranulum* species and a decreased abundance of *Bifidobacterium merycicum* and *Streptococcus lutetiensis* (at 4–6 months). Pathway analysis also revealed an association between antibiotic exposure and a decreased abundance of phenylalanine metabolism and an increased abundance of cyanoamino acid (3 and 4–6 months) and galactose metabolism (4–6 months) (Fig. 3; *p* value < 0.05). Analysis of metabolites showed associations between antibiotic exposure and a number of metabolites including decreased sucrose abundance (at 4–6 months) (Fig. 4; *p* value < 0.05).

### Changes in the microbiota of environmentally exposed vs. non-exposed infants

**Fig. 5:**
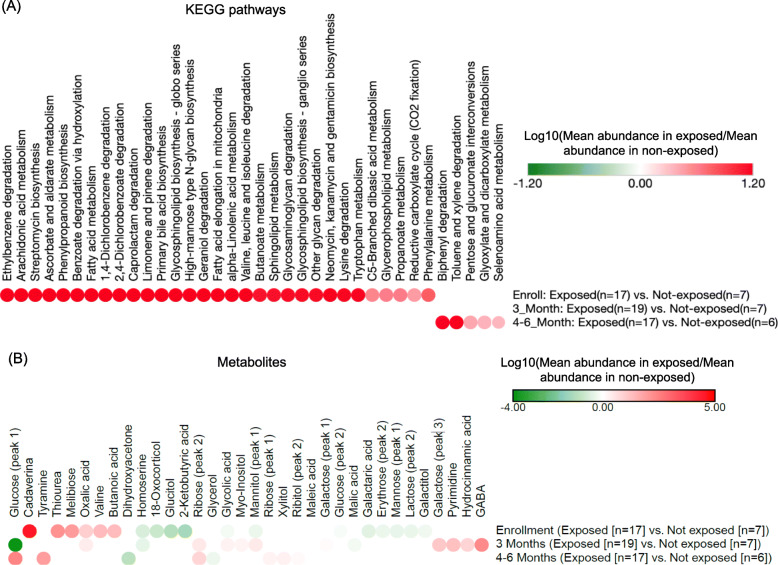
Cross-sectional analysis of microbiota features for genetically predisposed infants. **a** functional pathways (*p* value < 0.05), and **b** metabolites that are differentially abundant between environmentally exposed and non-exposed infants according to Mann-Whitney *U* test (*p* value < 0.05). Additional File 8 for grouping of pathways based on KEGG categorizations. See Additional File 9 for boxplots showing altered abundances for these pathways and metabolites. Brackets show time points at which a significant difference between the exposed and non-exposed groups was observed

Here, we performed a cross-sectional (inter-subject) analysis to explore how various features of the gut microbiota (microbes, pathways, and metabolites) change between genetically predisposed infants who were exposed to at least one environmental risk factor noted before (environmentally exposed infants) vs. those who were not (environmentally non-exposed infants) (Fig. 5). This analysis did not identify any microbial species whose abundance is significantly different between the environmentally exposed and non-exposed infants. Pathways analysis, however, revealed that environmentally exposed infants have a higher abundance of pathways for xenobiotic degradation, fatty acid metabolism, and lipid metabolism among others (at enrollment) and of pathways such as toluene and xylene and biphenyl degradation (at 4–6 months) (Fig. 5a; *p* value < 0.05). Metabolomic analysis identified alterations such as a decreased abundance of homoserine (at enrollment and 3 months) and of 2-ketobutryic acid (at enrollment) as well as an increased abundance of ribose (peak 2) (at 3 and 4–6 months) in environmentally exposed infants compared to non-exposed infants (Fig. 5b; *p* value < 0.05).

### Longitudinal changes in the microbiota of environmentally exposed and non-exposed infants

**Fig. 6:**
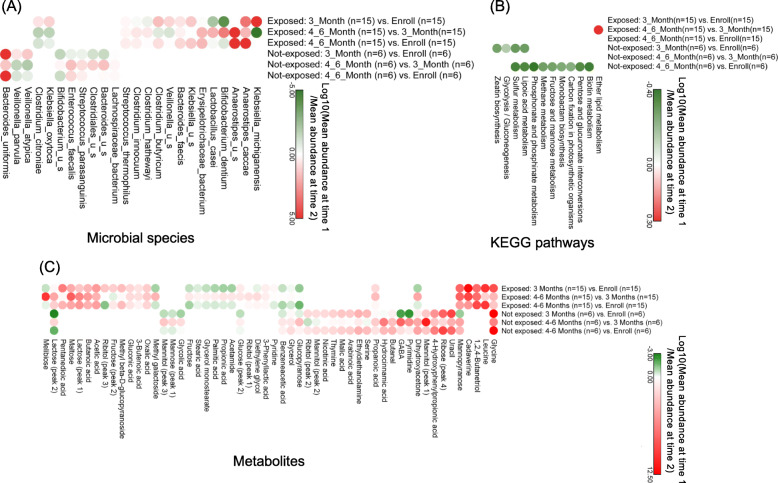
Longitudinal analysis of microbiota features for genetically predisposed infants **a** microbial species, **b** functional pathways, and **c** metabolites that are differentially abundant between each pair of time points (enrollment, 3 months, and 4–6 months) according to a paired Wilcoxon (Wilcoxon signed rank) test (*p* value < 0.05). Here, “Time1” denotes the earlier time point. In this figure, “u_s” denotes and unspecified species. Additional File 8 for grouping of pathways based on KEGG categorizations. See Additional File 9 for boxplots showing altered abundances for these pathways and metabolites

Given the unique prospective study design of our cohort, we were able to perform a longitudinal (intra-subject) analysis to gain additional insights beyond a cross-sectional analysis by identifying dynamic alterations in the gut microbiota composition, function, and metabolome in the first 6 months after birth. To this end, we explored changes in the microbiota features noted above between all pairs of time points that are observed exclusively in environmentally exposed or exclusively in environmentally non-exposed infants (Fig. 6).

By longitudinal analysis of microbial species, we found that the abundance of a number of species increases over time in the environmentally exposed infants (Fig. 6a; *p* value < 0.05). For example, the abundance of *Anaerostipes caccae* monotonically increases during the study period and that of *Klebsiella* species and *Erysipelotrichaceae bacterium* increases from enrollment to 4–6 months. Among these, *Klebsiella* has been associated with the autoimmune condition ankylosing spondylitis [51]. When examining environmentally non-exposed infants, we observe that the abundance of *Bacteroides uniformis* monotonically increases during the first 6 months after birth, a pattern which has previously been reported in breastmilk-fed infants [52]. In addition, work in mice found that *Bacteroides uniformis* improves immune defense mechanisms, which are impaired in obesity, by decreasing TNF-α production and increasing IL-10 production [53]. In our study, we also observed a decrease in the abundance of *Veillonella* species from enrollment to 4–6 months in non-exposed infants. An increased abundance of *Veillonella* species has been associated with autoimmune hepatitis [35].

Longitudinal pathway analysis revealed that the abundance of ether lipid metabolism increases from 3 to 4–6 months of age in environmentally exposed infants (Fig. 6b; *p* value < 0.05). Notably, a decreased abundance of ether lipids in the serum of children with T1D compared to healthy controls has been observed, [54] although the relationship between the abundance of microbial pathways for ether lipid metabolism in the gut and the level of ether lipids in the serum are yet to be explored. For the non-exposed infants, we observe a decrease in the abundance of sulfur metabolism and lipoic acid metabolism at 3 and 4–6 months, and of methane metabolism and biotin metabolism at 4–6 months compared to enrollment. These patterns are consistent with previous reports [22, 55–61]. For example, increased sulfur metabolism is associated with the development of T1D [55] and is linked to IBD [22]. Additionally, lipoic acid is an antioxidant that has been suggested to have beneficial immunomodulatory effects on the innate and adaptive immune systems in autoimmune diseases [56]. Methane has also been shown to have an anti-inflammatory effect, promoting immune tolerance in the intestine when tested in animal models [57, 58]. Furthermore, biotin is known to enhance innate [59] and adaptive immune responses [60] and biotin deficiency has been associated with immune disorders and inflammation [62, 63]. A previous study also found that high dose of biotin may be useful in treating multiple sclerosis [61].

Metabolomic analysis revealed a monotonic increase in erythritol abundances during the study period and a decrease in propionic acid abundance from enrollment to 4–6 months in environmentally exposed infants (Fig. 6c; *p* value < 0.05). Propionic acid produced in the colon via bacterial fermentation of fiber promotes regulatory T cell generation [64]. Additionally, increased serum levels of erythritol have been associated with central obesity and weight gain [65], though the link between metabolite levels in the gut and those in the serum is not clear. In environmentally non-exposed infants, we observed an increased abundance of uracil, 3-3-hydroxyphenylpropionic acid, and dihydroxyacetone from enrollment to 4–6 months. Previous work suggests that 3-hydroxyphenylproprionic acid acts as an anti-inflammatory and antioxidant agent [66].

### Linking microbial species, pathways, and metabolites

In order to link microbial species, pathways and metabolites identified in these analyses, we performed a correlation analysis (using Spearman rank correlation) as detailed in Additional File 6, which resulted in several significant correlations between these features as summarized in Additional File 7. For example, exploring the links between pathways and metabolites with altered abundance in the cross-sectional analysis identified positive associations between ribose (peak 2) and biphenyl degradation and between toluene and xylene degradation in the environmentally exposed infants. In addition, association analysis between significant pathways and metabolites in the longitudinal analysis identified a negative association at 3 and 4–6 months between 3-3-hydroxyphenylpropionic acid and sulfur, lipoic acid, methane, and biotin metabolism in non-exposed infants (Additional File 7).

## Discussion

Several studies have linked exposure to a variety of genetic and environmental risk factors with the onset of non-infective chronic inflammatory diseases [13]. This link has been typically based on the results obtained from either clinical case-control studies [14, 15, 19, 67] or metanalyses [68–71] in which cause-effect relationship cannot always conclusively be determined. Since host genetics and environmental factors are known to influence the gut microbiota composition and function, researchers have started to explore alterations in the gut microbiota of infants at risk of autoimmune conditions such as IBD [31] or T1D [32]. However, to date, there is no systematic study of how protective or detrimental genetic and environmental factors may change the gut microbiota engraftment and its maturation during the first months of life in infants at-risk of CD. In an effort to fill this gap, in this study, we used metagenomic and metabolomic data collected in the first 6 months after birth to associate individual risk factors (HLA DQ2/DQ8 genetics, cesarean section delivery, antibiotic use, and partial or exclusive formula feeding) with microbial species, pathways, and metabolites in the gut. Additionally, we performed cross-sectional analysis to identify microbes, pathways, and metabolites that are differentially abundant between infants exposed to at least one environmental risk factor and infants who were not, as well as longitudinal analysis to identify dynamic changes in the gut microbiota in the first 6 months of life. Notably, we restricted our analysis only to the first 6 months after birth prior to the introduction of solid foods in order to focus exclusively on the effect of the genetic predisposition and early environmental exposures on the development of the gut microbiota in at-risk infants without any noise from differences in infants diets including gluten.

Many microbes, pathways, or metabolites that we identified in these analyses are well supported in the literature to be associated with inflammation, autoimmune disease, or immune system dysfunction, thereby suggesting that they may have similar effects in CD. For example, we found that high-risk HLA genetics and formula feeding are both associated with an increased abundance of *Ruminococcus gnavus* and *Lachnospiraceae bacterium*, which are linked to allergic diseases [47] and diabetes [48], respectively. Among other significant findings are associations between cesarean section delivery and a decreased abundance of *Bacteroides vulgatus* and *Bacteroides dorei* and folate biosynthesis pathway along with an increased abundance of hydroxyphenlacetic acid. All of these patterns have been reported to be associated with impaired immune function [41] and inflammatory conditions such as T1D and ulcerative colitis [42, 43] suggesting that they could also predispose infants to develop CD.

While the cross-sectional analysis did not identify any microbial species whose abundance significantly changes between the environmentally exposed and non-exposed infants at any given time point, our longitudinal analysis yielded significant results further stressing the power of intra-subject analysis. This allows us to prospectively evaluate the impact of risk factors on the dynamics of the gut microbiota development and to link dynamics to increased susceptibility to inflammation. For example, environmentally exposed infants show an increasing abundance over time of *Klebsiella* species, a microbe linked to autoimmune disease [51] and a decreasing abundance over time of propionic acid, a metabolite that promotes innate and adaptive immunity [64]. In contrast, in infants not exposed to environmental risk factors, we observe patterns associated with beneficial immunomodulatory effects and protection against immune system activation and inflammation such as increasing abundance of *Bacteroides uniformis* over time and decreasing abundance of lipoic acid and methane metabolism [53, 56–58, 66]. Notably, during our analyses, we identified a number of metabolites and pathways with altered abundances in the gut (including hydroxyphenlacetic acid, erythritol, and ether lipid metabolism) for which similar variations in the serum are reported to be associated with autoimmune conditions. While the importance of gut-blood axis has been realized fairly recently [72, 73], further investigations are needed to better understand the relationship between different features of the gut microbiota and host- or microbially derived metabolites in the blood.

Unlike previous microbiome studies for CD that are often based on 16S rRNA amplicon sequencing, here, we use shotgun metagenomic sequencing, which is amenable to functional characterization of the microbiota. This is particularly important as previous studies have shown that functional characterization is a more robust descriptor of the status of the microbiota compared to taxonomic composition alone [74, 75]. Furthermore, unlike typical case-control studies, where disease symptoms have already emerged in cases, our prospective birth cohort provides the opportunity to mechanistically link major shifts in the gut microbiota early in life, due to genetic risk factors and environmental exposures, in infants at-risk of CD. Nevertheless, our data should be considered exploratory given the relatively small sample size. This limitation can be mitigated through ongoing recruitment into our CDGEMM cohort, which will allow us to validate our findings using a much larger number of subjects in the future.

## Conclusions

In this paper, we utilized an ongoing prospective study and multi-omics analysis to perform an in-depth analysis of the impact of genetic and environmental risk factors on the longitudinal development of the gut microbiota in infants at risk for CD, before solid foods (including the trigger of CD, gluten) are introduced. These analyses revealed several microbial species, functional pathways and metabolites that have been previously linked to inflammation or immune system dysfunction as well as several new ones that have not been reported before and could be specific to CD. In this study, we restricted our analysis to the first 6 months of life and particularly prior to the introduction of solid foods in order to proactively “regress out” the effect of gluten on the gut microbiota as a major confounder when analyzing the effect of genetic and environmental risk factors. However, while our analysis suggests that the microbiome shifts that we observed during the first 6 months after birth increase the risk of developing autoimmune conditions including CD based on existing literature, it is unclear whether they indeed contribute to the future development of CD. Therefore, further work is required to investigate alterations in the gut microbiota over a longer period of time, including through the onset of CD. Future work should also consider other environmental factors such as viral infections, timing of solid food (gluten) introduction, amount of gluten ingested, and household exposures, e.g. family size and contact with pets, which have been reported to be associated with altered microbiomes [76], or with protection against autoimmune conditions such as asthma [77] and T1D [78]. These investigations warrant future studies, which can utilize this longitudinal study design and multi-omics analysis as a basis to connect alterations in the gut microbiota early in life to the loss of tolerance to gluten and the development of CD.

## Methods

### Subjects, sampling, and factors of interest

Thirty-one healthy infants from the USA (*n* = 18) and Italy (*n* = 13) with a first-degree relative with CD participating in the CDGEMM prospective birth cohort study [33] were included in our analysis. These subjects consist of all infants from CDGEMM with available stool samples collected before the introduction of solid foods at 7–15 days (enrollment), 3 months, and 4–6 months after birth. Parents answered a detailed questionnaire at enrollment that addressed pregnancy, delivery, family history, household factors, and many other factors related to the infants’ environment before birth and at delivery. Parents also filled out monthly diaries, which addressed infant food intake and any exposure to antibiotics. Infant feeding type was determined according to the reported exclusive feeding type for at least two of the three sample time point collections. Infants who received both breastmilk and formula for at least two of three sample collection points were classified as “both breastmilk and formula fed.” HLA genetic type was determined from whole blood at time of birth (cord blood) or 12 months of age using the DQ-CD Typing Plus (BioDiagne, Palermo, Italy) per the manufacturer’s instructions. Written informed consent was obtained from the parents of infants included in the study according to the standards outlined and approved by the Partners Human Research Committee Institutional Review Board.

### DNA extraction

All fecal samples included in the metagenomic analysis were stored and processed centrally in the USA. Total DNA from each sample was extracted using the Qiagen Power soil DNA extraction kit (Qiagen, Hilden, Germany).

### Taxonomic profiling

General sequencing statistics of all samples, as well as mean sequence quality distribution for metagenomics samples were measured by MultiQC [79]. Since the mean quality value across each base position in the trimmed reads obtained with mulitQC was above quality score 17 for at least 80% of the read length (i.e., probability of correct base call ~ 98%), reads were not subjected to additional quality trimming steps before uploading to the CosmosID cloud platform. Metagenomic sequencing reads were then analyzed by using the CosmosID’s (CosmosID Inc., Rockville, MD) commercial metagenomic analysis platform (formerly knowns as GENIUS; https://app.cosmosid.com/login) [80, 81], which is based on an assembly-free kmer-based method, to reveal the underlying microbial community composition up to the species-level resolution (see Additional File 6 for a detailed description of this platform and Additional File 2 for information on the sequencing depth of each sample and the number of reads with a taxon assignment).

### Functional profiling

After trimming the raw sequencing reads using BBDuk (https://jgi.doe.gov/data-and-tools/bbtools/) (with parameters *minlen* = 25, *qtrim* = *rl*, *trimq* = 20), we used the SPAdes tool [82] (with parameter --*only*-*assembler* -*k* 77,99,12*7*) for the assembly of metagenomes and subsequently and after removing short contigs (length threshold = 500 bp), we used Prodigal (v2.6 using -*d* parameter) [83] to identify protein coding sequences in the assembled metagenomes. We then utilized InterProScan [84] (with parameters -*appl Hamap*, *ProDom* -*p* and -*f tsv*) to annotate the identified genes with biochemical functions based on the KEGG pathways [85]. The relative abundance of each gene was computed as $G=\frac{L\ast C}{\left(R-K+1\right)}$, where, *G* is fragments per kilobase per million (FPKM) for each gene, *L* is the length of the gene, *C* is the coverage of contig in which the gene is identified, *R* is the read length and *K* is the *k*-mer size [86]. The relative abundance of each KEGG pathway was then quantified by summing the relative abundances of all the genes associated to that pathway.

### Metabolomic profiling

All stool samples for metabolomics were stored and processed in Italy. The metabolome extraction, purification, and derivatization were carried by the MetaboPrep GC kit (Theoreo, Montecorvino Pugliano, Italy) according to manufacturer instructions. Instrumental analyses were performed with a GC-MS system (GC-2010 Plus gas chromatograph and QP2010 Plus mass spectrometer; Shimadzu Corp., Kyoto, Japan). Sample analysis was performed in triplicate. Additional information related to the extraction, purification, derivatization, GC-MS analysis, and data preprocessing can be found in Additional File 6. The molecular identity of metabolites was determined by analysis of the corresponding mass spectrum in the chromatogram, setting the linear index difference max tolerance to 10. These identified metabolites were further confirmed using external standards according to level 1 Metabolomics Standards Initiative (MSI) [87].

### Identifying associations between genetic and environmental risk factors and microbiome features

We used the widely used multivariate statistical framework, MaAsLin [22], to determine associations between microbial species, functional, pathways or metabolites and genetic and environmental risk factors including HLA genetics, delivery mode, infant feeding type, and antibiotic exposure at each time point. No genetic risk, vaginal delivery, exclusive breastmilk feeding, and no antibiotic exposure were considered the reference levels for HLA genetics, delivery mode, infant feeding type, and antibiotic exposure, respectively. All metadata variables were forced simultaneously to control for confounders. Significant results were reported using a *p* value threshold of 0.05.

### Cross-sectional and longitudinal analysis

For the cross-sectional analysis, we performed the Mann-Whitney *U* (Wilcoxon Rank Sum) test to compare the abundance of microbial species, pathways, and metabolites at each time point between the environmentally exposed and non-exposed groups (using a *p* value threshold of 0.05 to report significant results). For the longitudinal analysis, we performed the paired Wilcoxon (Wilcoxon Signed Rank) test to compare the abundances of microbial species, pathways, and metabolites between each pair of time points using the same *p* value threshold noted above to report the significant results. Analyses of microbial species and pathways were performed in Python (using scipy.stats.mannwhitneyu and scipy.stats.wilcoxon functions) and those for metabolites were performed in R (using Ttest.Anal function of the MetaboAnalyst package [88] using parameters *nonpar=TRUE* and *paired=FALSE* for the cross-sectional and *paired=TRUE* for the longitudinal analysis).

## Supplementary information

**Additional file 1:** Clinical metadata for the subjects in this study.

**Additional file 2:** Results of the taxonomic profiling of metagenomic samples.

**Additional file 3:** Taxonomic composition at the genus and family level for metagenomes.

**Additional file 4:** Results of the functional profiling of metagenomic samples.

**Additional file 5:** Results of the metabolomic profiling of stool samples.

**Additional file 6:** Supplementary text describing details of data analysis methods.

**Additional file 7:** The results of association studies between significant features (microbes, pathways and metabolites).

**Additional file 8:** Functional categorization of pathways with significantly altered abundances.

**Additional file 9:** Boxplots for significant features in the cross-sectional and longitudinal analysis

## Data Availability

The datasets supporting the conclusions of this article are submitted to the NCBI Short Read Archive (SRA) repository, under BioProjectID PRJNA486782 and SRA accession number SRP158417. Additional data from the analyses presented in this paper are available in the Supplementary Material.

## Abbreviations

CD: Celiac disease
HLA: Human leukocyte antigen
IBD: Inflammatory bowel disease
T1D: Type 1 diabetes

## References

[1] Lionetti E, Gatti S, Pulvirenti A, Catassi C (2015). Celiac disease from a global perspective. Best Pract Res Clin Gastroenterol.

[2] Schuppan D (2000). Current concepts of celiac disease pathogenesis. Gastroenterology.

[3] Green PH, Cellier C (2007). Celiac disease. N Engl J Med.

[4] Vecsei E, Steinwendner S, Kogler H, Innerhofer A, Hammer K, Haas OA (2014). Follow-up of pediatric celiac disease: value of antibodies in predicting mucosal healing, a prospective cohort study. BMC Gastroenterol.

[5] Leonard MM, Weir DC, DeGroote M, Mitchell PD, Singh P, Silvester JA (2017). Value of IgA tTG in predicting mucosal recovery in children with celiac disease on a gluten-free diet. J Pediatr Gastroenterol Nutr.

[6] Ciacci C, Cirillo M, Cavallaro R, Mazzacca G (2002). Long-term follow-up of celiac adults on gluten-free diet: prevalence and correlates of intestinal damage. Digestion.

[7] Rubio-Tapia A, Rahim MW, See JA, Lahr BD, Wu TT, Murray JA (2010). Mucosal recovery and mortality in adults with celiac disease after treatment with a gluten-free diet. Am J Gastroenterol.

[8] Valitutti F, Trovato CM, Montuori M, Cucchiara S (2017). Pediatric celiac disease: follow-up in the spotlight. Adv Nutr.

[9] Valitutti F, Fasano A (2019). Breaking down barriers: how understanding celiac disease pathogenesis informed the development of novel treatments. Dig Dis Sci.

[10] West J, Fleming KM, Tata LJ, Card TR, Crooks CJ (2014). Incidence and prevalence of celiac disease and dermatitis herpetiformis in the UK over two decades: population-based study. Am J Gastroenterol.

[11] Catassi C, Kryszak D, Bhatti B, Sturgeon C, Helzlsouer K, Clipp SL (2010). Natural history of celiac disease autoimmunity in a USA cohort followed since 1974. Ann Med.

[12] Ricano-Ponce I, Wijmenga C, Gutierrez-Achury J (2015). Genetics of celiac disease. Best Pract Res Clin Gastroenterol.

[13] Tamburini S, Shen N, Wu HC, Clemente JC (2016). The microbiome in early life: implications for health outcomes. Nat Med.

[14] Decker E, Engelmann G, Findeisen A, Gerner P, Laass M, Ney D (2010). Cesarean delivery is associated with celiac disease but not inflammatory bowel disease in children. Pediatrics.

[15] Marild K, Stephansson O, Montgomery S, Murray JA, Ludvigsson JF (2012). Pregnancy outcome and risk of celiac disease in offspring: a nationwide case-control study. Gastroenterology.

[16] Akobeng AK, Ramanan AV, Buchan I, Heller RF (2006). Effect of breast feeding on risk of coeliac disease: a systematic review and meta-analysis of observational studies. Arch Dis Child.

[17] Szajewska H, Chmielewska A, Piescik-Lech M, Ivarsson A, Kolacek S, Koletzko S (2012). Systematic review: early infant feeding and the prevention of coeliac disease. Aliment Pharmacol Ther.

[18] Norris JM, Barriga K, Hoffenberg EJ, Taki I, Miao D, Haas JE (2005). Risk of celiac disease autoimmunity and timing of gluten introduction in the diet of infants at increased risk of disease. JAMA.

[19] Marild K, Ye W, Lebwohl B, Green PH, Blaser MJ, Card T (2013). Antibiotic exposure and the development of coeliac disease: a nationwide case-control study. BMC Gastroenterol.

[20] Lionetti E, Castellaneta S, Francavilla R, Pulvirenti A, Tonutti E, Amarri S (2014). Introduction of gluten, HLA status, and the risk of celiac disease in children. N Engl J Med.

[21] Vriezinga SL, Auricchio R, Bravi E, Castillejo G, Chmielewska A, Crespo Escobar P (2014). Randomized feeding intervention in infants at high risk for celiac disease. N Engl J Med.

[22] Morgan XC, Tickle TL, Sokol H, Gevers D, Devaney KL, Ward DV (2012). Dysfunction of the intestinal microbiome in inflammatory bowel disease and treatment. Genome Biol.

[23] Stewart CJ, Ajami NJ, O’Brien JL, Hutchinson DS, Smith DP, Wong MC (2018). Temporal development of the gut microbiome in early childhood from the TEDDY study. Nature.

[24] Jangi S, Gandhi R, Cox LM, Li N, Von Glehn F, Yan R (2016). Alterations of the human gut microbiome in multiple sclerosis. Nat Commun.

[25] De Palma G, Capilla A, Nadal I, Nova E, Pozo T, Varea V (2010). Interplay between human leukocyte antigen genes and the microbial colonization process of the newborn intestine. Curr Issues Mol Biol.

[26] Olivares M, Neef A, Castillejo G, Palma GD, Varea V, Capilla A (2015). The HLA-DQ2 genotype selects for early intestinal microbiota composition in infants at high risk of developing coeliac disease. Gut.

[27] Sellitto M, Bai G, Serena G, Fricke WF, Sturgeon C, Gajer P (2012). Proof of concept of microbiome-metabolome analysis and delayed gluten exposure on celiac disease autoimmunity in genetically at-risk infants. PLoS One.

[28] Pozo-Rubio T, de Palma G, Mujico JR, Olivares M, Marcos A, Acuna MD (2013). Influence of early environmental factors on lymphocyte subsets and gut microbiota in infants at risk of celiac disease; the PROFICEL study. Nutr Hosp.

[29] Olivares M, Walker AW, Capilla A, Benitez-Paez A, Palau F, Parkhill J (2018). Gut microbiota trajectory in early life may predict development of celiac disease. Microbiome.

[30] Rintala A, Riikonen I, Toivonen A, Pietila S, Munukka E, Pursiheimo JP, et al. Early fecal microbiota composition in children who later develop celiac disease and associated autoimmunity. Scand J Gastroenterol. 2018:1–7. 10.1080/00365521.2018.1444788 29504486

[31] Torres J, Hu J, Seki A, Eisele C, Nair N, Huang R (2020). Infants born to mothers with IBD present with altered gut microbiome that transfers abnormalities of the adaptive immune system to germ-free mice. Gut.

[32] Vatanen T, Franzosa EA, Schwager R, Tripathi S, Arthur TD, Vehik K (2018). The human gut microbiome in early-onset type 1 diabetes from the TEDDY study. Nature.

[33] Leonard MM, Camhi S, Huedo-Medina TB, Fasano A (2015). Celiac disease genomic, environmental, microbiome, and Metabolomic (CDGEMM) study design: approach to the future of personalized prevention of celiac disease. Nutrients.

[34] Hov JR, Zhong H, Qin B, Anmarkrud JA, Holm K, Franke A, Lie BA, Karlsen TH: The influence of the autoimmunity-associated ancestral HLA haplotype AH8. 1 on the human gut microbiota: a cross-sectional study. PLoS One 2015, 10(7):e0133804. 10.1371/journal.pone.0133804 PMC4514645 26207384

[35] Wei Y, Li Y, Yan L, Sun C, Miao Q, Wang Q, et al. Alterations of gut microbiome in autoimmune hepatitis. Gut. 2019. 10.1136/gutjnl-2018-317836 31201284

[36] Ye Z, Zhang N, Wu C, Zhang X, Wang Q, Huang X (2018). A metagenomic study of the gut microbiome in Behcet’s disease. Microbiome.

[37] Cree BA, Spencer CM, Varrin-Doyer M, Baranzini SE, Zamvil SS (2016). Gut microbiome analysis in neuromyelitis optica reveals overabundance of Clostridium perfringens. Ann Neurol.

[38] Shao Y, Forster SC, Tsaliki E, Vervier K, Strang A, Simpson N, et al. Stunted microbiota and opportunistic pathogen colonization in caesarean-section birth. Nature. 2019:1–5. 10.1038/s41586-019-1560-1 PMC6894937 31534227

[39] Bokulich NA, Chung J, Battaglia T, Henderson N, Jay M, Li H, Lieber AD, Wu F, Perez-Perez GI, Chen Y: Antibiotics, birth mode, and diet shape microbiome maturation during early life. Science translational medicine 2016, 8(343):343ra382-343ra382. 10.1126/scitranslmed.aad7121 PMC5308924 27306664

[40] Wampach L, Heintz-Buschart A, Fritz JV, Ramiro-Garcia J, Habier J, Herold M (2018). Birth mode is associated with earliest strain-conferred gut microbiome functions and immunostimulatory potential. Nat Commun.

[41] Yoshida N, Emoto T, Yamashita T, Watanabe H, Hayashi T, Tabata T (2018). Bacteroides vulgatus and Bacteroides dorei reduce gut microbial lipopolysaccharide production and inhibit atherosclerosis. Circulation.

[42] Bayer AL, Fraker CA (2017). The folate cycle as a cause of natural killer cell dysfunction and viral etiology in type 1 diabetes. Front Endocrinol (Lausanne).

[43] Sitkin SI, Tkachenko EI, Vakhitov T, Oreshko LS (2013). Zhigalova TN: [serum metabolome by gas chromatography-mass spectrometry (GC-MS) in patients with ulcerative colitis and celiac disease]. Eksp Klin Gastroenterol.

[44] Ma EH, Bantug G, Griss T, Condotta S, Johnson RM, Samborska B (2017). Serine is an essential metabolite for effector T cell expansion. Cell Metab.

[45] Bäckhed F, Roswall J, Peng Y, Feng Q, Jia H, Kovatcheva-Datchary P (2015). Dynamics and stabilization of the human gut microbiome during the first year of life. Cell Host Microbe.

[46] Lundequist B, Nord CE, Winberg J (1985). The composition of the faecal microflora in breastfed and bottle fed infants from birth to eight weeks. Acta Paediatr Scand.

[47] Chua HH, Chou HC, Tung YL, Chiang BL, Liao CC, Liu HH (2018). Intestinal dysbiosis featuring abundance of Ruminococcus gnavus associates with allergic diseases in infants. Gastroenterology.

[48] Kameyama K, Itoh K: Intestinal colonization by a Lachnospiraceae bacterium contributes to the development of diabetes in obese mice. Microbes and environments 2014:ME14054. 10.1264/jsme2.ME14054 PMC4262368 25283478

[49] Zeng H, Ishaq SL, Zhao F-Q, Wright A-DG (2016). Colonic inflammation accompanies an increase of β-catenin signaling and Lachnospiraceae/Streptococcaceae bacteria in the hind gut of high-fat diet-fed mice. J Nutr Biochem.

[50] Cabral DJ, Penumutchu S, Reinhart EM, Zhang C, Korry BJ, Wurster JI, Nilson R, Guang A, Sano WH, Rowan-Nash AD: Microbial metabolism modulates antibiotic susceptibility within the murine gut microbiome. Cell metabolism 2019, 30(4):800-823. e807. 10.1016/j.cmet.2019.08.020 PMC6948150 31523007

[51] Wilson C, Tiwana H, Ebringer A (2000). Molecular mimicry between HLA-DR alleles associated with rheumatoid arthritis and Proteus mirabilis as the aetiological basis for autoimmunity. Microbes Infect.

[52] Sanchez E, De Palma G, Capilla A, Nova E, Pozo T, Castillejo G (2011). Influence of environmental and genetic factors linked to celiac disease risk on infant gut colonization by Bacteroides species. Appl Environ Microbiol.

[53] Cano PG, Santacruz A, Moya Á, Sanz Y (2012). Bacteroides uniformis CECT 7771 ameliorates metabolic and immunological dysfunction in mice with high-fat-diet induced obesity. PLoS One.

[54] Orešič M, Simell S, Sysi-Aho M, Näntö-Salonen K, Seppänen-Laakso T, Parikka V (2008). Dysregulation of lipid and amino acid metabolism precedes islet autoimmunity in children who later progress to type 1 diabetes. J Exp Med.

[55] Brown CT, Davis-Richardson AG, Giongo A, Gano KA, Crabb DB, Mukherjee N (2011). Gut microbiome metagenomics analysis suggests a functional model for the development of autoimmunity for type 1 diabetes. PLoS One.

[56] Liu W, Shi L-J, Li S-G. The immunomodulatory effect of alpha-lipoic acid in autoimmune diseases. BioMed Res Int. 2019;2019. 10.1155/2019/8086257 PMC6446120 31016198

[57] Boros M, Ghyczy M, Érces D, Varga G, Tokés T, Kupai K (2012). The anti-inflammatory effects of methane. Crit Care Med.

[58] Zhang X, Li N, Shao H, Meng Y, Wang L, Wu Q (2016). Methane limit LPS-induced NF-κB/MAPKs signal in macrophages and suppress immune response in mice by enhancing PI3K/AKT/GSK-3β-mediated IL-10 expression. Sci Rep.

[59] Agrawal S, Agrawal A, Said HM (2016). Biotin deficiency enhances the inflammatory response of human dendritic cells. Am J Phys Cell Phys.

[60] Kung JT, Mackenzie CG, Talmage DW (1979). The requirement for biotin and fatty acids in the cytotoxic T-cell response. Cell Immunol.

[61] Sedel F, Bernard D, Mock DM, Tourbah A (2016). Targeting demyelination and virtual hypoxia with high-dose biotin as a treatment for progressive multiple sclerosis. Neuropharmacology.

[62] Abad-Lacruz A, Fernandez-Banares F, Cabre E, Gil A, Esteve M, Gonzalez-Huix F (1988). The effect of total enteral tube feeding on the vitamin status of malnourished patients with inflammatory bowel disease. International journal for vitamin and nutrition research Internationale Zeitschrift fur Vitamin-und Ernahrungsforschung Journal international de vitaminologie et de nutrition.

[63] Fernandez-Banares F, Abad-Lacruz A, Xiol X, Gine J, Dolz C, Cabre E, Esteve M, Gonzalez-Huix F, Gassull M. Vitamin status in patients with inflammatory bowel disease. Am J Gastroenterol. 1989:84(7). 2500847

[64] Arpaia N, Campbell C, Fan X, Dikiy S, van der Veeken J, de Roos P, Liu H, Cross JR, Pfeffer K, Coffer PJ (2013). Metabolites produced by commensal bacteria promote peripheral regulatory T-cell generation. Nature.

[65] Hootman KC, Trezzi J-P, Kraemer L, Burwell LS, Dong X, Guertin KA (2017). Erythritol is a pentose-phosphate pathway metabolite and associated with adiposity gain in young adults. Proc Natl Acad Sci.

[66] Fan FY, Sang LX, Jiang M. Catechins and their therapeutic benefits to inflammatory bowel disease. Molecules. 2017:22(3). 10.3390/molecules22030484 PMC6155401 28335502

[67] Baron S, Turck D, Leplat C, Merle V, Gower-Rousseau C, Marti R (2005). Environmental risk factors in paediatric inflammatory bowel diseases: a population based case control study. Gut.

[68] Xu L, Lochhead P, Ko Y, Claggett B, Leong RW, Ananthakrishnan AN (2017). Systematic review with meta-analysis: breastfeeding and the risk of Crohn’s disease and ulcerative colitis. Aliment Pharmacol Ther.

[69] Ungaro R, Bernstein CN, Gearry R, Hviid A, Kolho KL, Kronman MP (2014). Antibiotics associated with increased risk of new-onset Crohn’s disease but not ulcerative colitis: a meta-analysis. Am J Gastroenterol.

[70] Costenbader KH, Kim DJ, Peerzada J, Lockman S, Nobles-Knight D, Petri M (2004). Cigarette smoking and the risk of systemic lupus erythematosus: a meta-analysis. Arthritis Rheum.

[71] McCormic ZD, Khuder SS, Aryal BK, Ames AL, Khuder SA (2010). Occupational silica exposure as a risk factor for scleroderma: a meta-analysis. Int Arch Occup Environ Health.

[72] Wilmanski T, Rappaport N, Earls JC, Magis AT, Manor O, Lovejoy J (2019). Blood metabolome predicts gut microbiome α-diversity in humans. Nat Biotechnol.

[73] Analysis of blood and fecal microbiome profile in patients with celiac disease. Human Microbiome Journal 2019, 11.

[74] Abubucker S, Segata N, Goll J, Schubert AM, Izard J, Cantarel BL (2012). Metabolic reconstruction for metagenomic data and its application to the human microbiome. PLoS Comput Biol.

[75] Human Microbiome Project C (2012). Structure, function and diversity of the healthy human microbiome. Nature.

[76] Sjogren YM, Jenmalm MC, Bottcher MF, Bjorksten B, Sverremark-Ekstrom E (2009). Altered early infant gut microbiota in children developing allergy up to 5 years of age. Clin Exp Allergy.

[77] Ownby DR, Johnson CC, Peterson EL (2002). Exposure to dogs and cats in the first year of life and risk of allergic sensitization at 6 to 7 years of age. JAMA.

[78] Virtanen SM, Takkinen HM, Nwaru BI, Kaila M, Ahonen S, Nevalainen J (2014). Microbial exposure in infancy and subsequent appearance of type 1 diabetes mellitus-associated autoantibodies: a cohort study. JAMA Pediatr.

[79] Ewels P, Magnusson M, Lundin S, Kaller M (2016). MultiQC: summarize analysis results for multiple tools and samples in a single report. Bioinformatics.

[80] Hasan NA, Young BA, Minard-Smith AT, Saeed K, Li H, Heizer EM (2014). Microbial community profiling of human saliva using shotgun metagenomic sequencing. PLoS One.

[81] Ponnusamy D, Kozlova EV, Sha J, Erova TE, Azar SR, Fitts EC (2016). Cross-talk among flesh-eating Aeromonas hydrophila strains in mixed infection leading to necrotizing fasciitis. Proc Natl Acad Sci U S A.

[82] Bankevich A, Nurk S, Antipov D, Gurevich AA, Dvorkin M, Kulikov AS (2012). SPAdes: a new genome assembly algorithm and its applications to single-cell sequencing. J Comput Biol.

[83] Hyatt D, Chen G-L, LoCascio PF, Land ML, Larimer FW, Hauser LJ (2010). Prodigal: prokaryotic gene recognition and translation initiation site identification. BMC bioinformatics.

[84] Jones P, Binns D, Chang H-Y, Fraser M, Li W, McAnulla C (2014). InterProScan 5: genome-scale protein function classification. Bioinformatics.

[85] Kanehisa M, Goto S (2000). KEGG: Kyoto encyclopedia of genes and genomes. Nucleic Acids Res.

[86] Zerbino DR, Birney E. Velvet: algorithms for de novo short read assembly using de Bruijn. Genome Res. 2004. 10.1101/gr.074492.107 PMC2336801 18349386

[87] Sumner LW, Amberg A, Barrett D, Beale MH, Beger R, Daykin CA (2007). Proposed minimum reporting standards for chemical analysis. Metabolomics.

[88] Chong J, Soufan O, Li C, Caraus I, Li S, Bourque G (2018). MetaboAnalyst 4.0: towards more transparent and integrative metabolomics analysis. Nucleic Acids Res.

