# Supplementary material for: Multi-omics analysis reveals the influence of genetic and environmental risk factors on developing gut microbiota in infants at risk of celiac disease
Source: Microbiome. 2020 Sep 11;8:130. doi: 10.1186/s40168-020-00906-w (PMC7488762; doi:10.1186/s40168-020-00906-w)
Supplement: Supplementary file 4 — Additional file 3: Taxonomic composition at the genus and family level for metagenomes. [file 40168_2020_906_MOESM3_ESM.pdf]

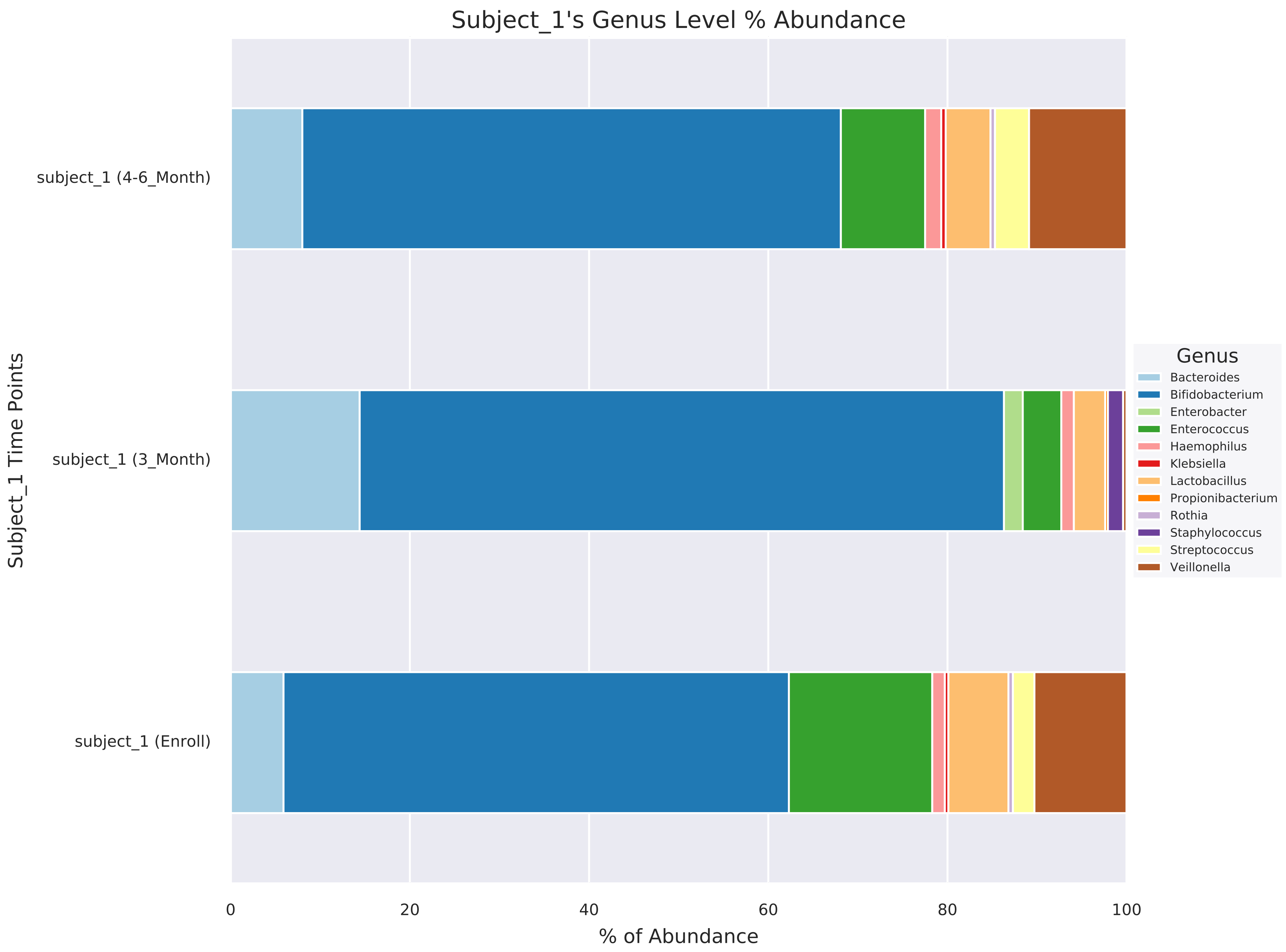
Subject_1's Genus Level % Abundance
subject_1 (4-6_Month)
subject_1 (3_Month)
subject_1 (Enroll)
Subject_1 Time Points
0
20
40
60
80
100
% of Abundance
Genus
Bacteroides
Bifidobacterium
Enterobacter
Enterococcus
Haemophilus
Klebsiella
Lactobacillus
Propionibacterium
Rothia
Staphylococcus
Streptococcus
Veillonella

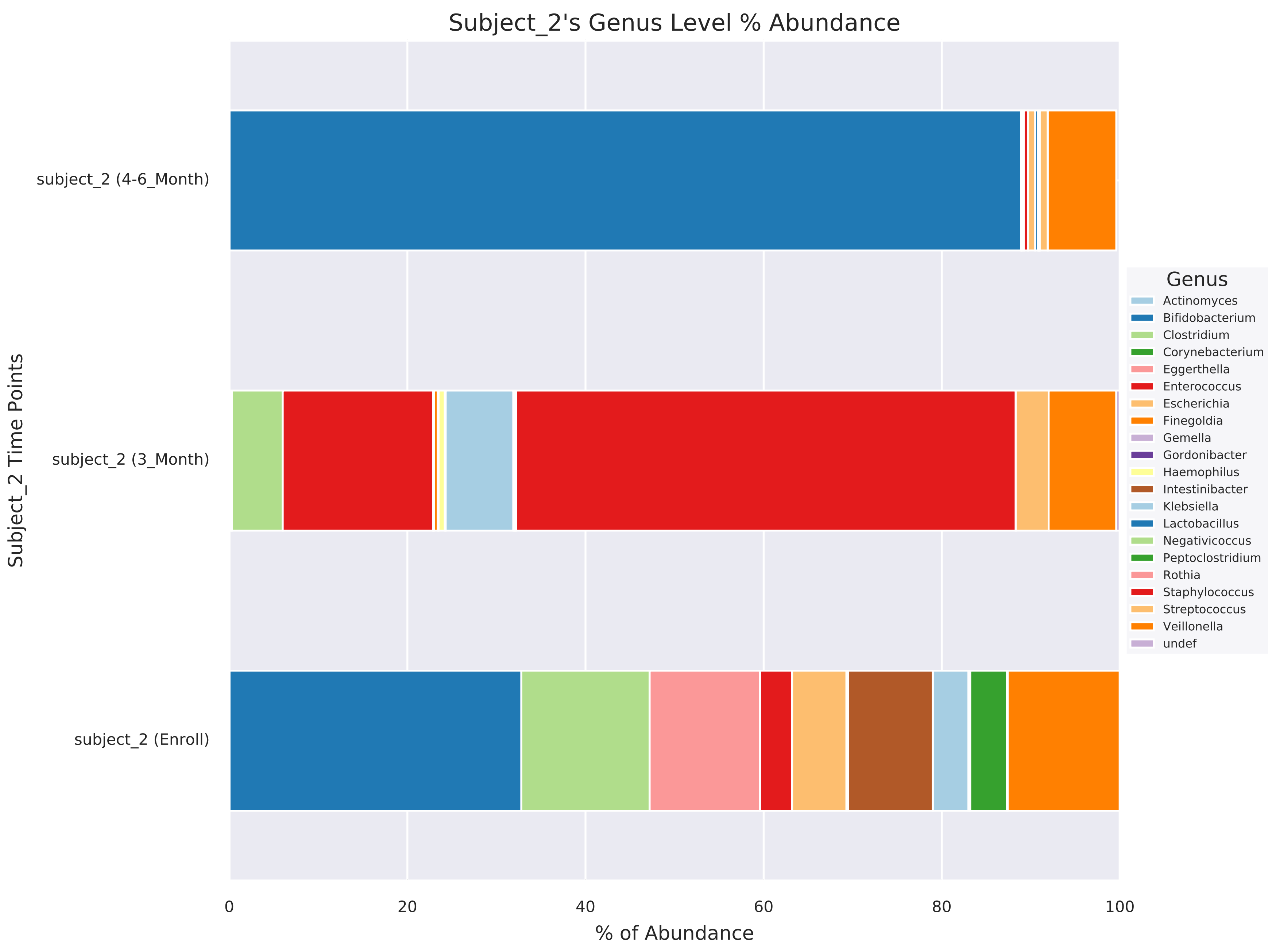

Subject_2's Genus Level % Abundance
Subject_2 Time Points
subject_2 (4-6_Month)
subject_2 (3_Month)
subject_2 (Enroll)
% of Abundance
0
20
40
60
80
100
Genus
Actinomyces
Bifidobacterium
Clostridium
Corynebacterium
Eggerthella
Enterococcus
Escherichia
Finegoldia
Gemella
Gordonibacter
Haemophilus
Intestinibacter
Klebsiella
Lactobacillus
Negativicoccus
Peptoclostridium
Rothia
Staphylococcus
Streptococcus
Veillonella
undef

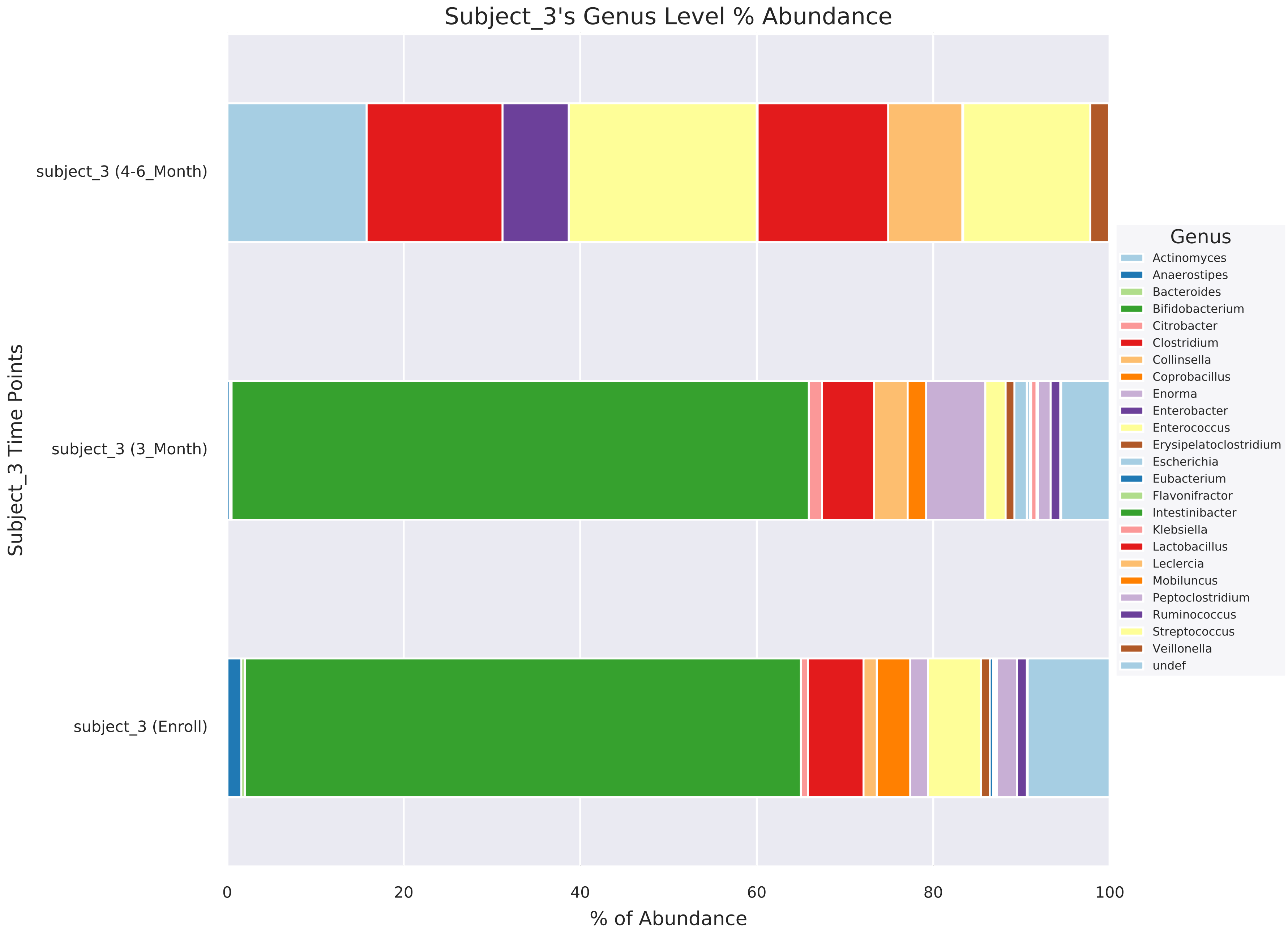
Subject_3's Genus Level % Abundance
Subject_3 Time Points
subject_3 (4-6_Month)
subject_3 (3_Month)
subject_3 (Enroll)
0
20
40
60
80
100
% of Abundance
Genus
Actinomyces
Anaerostipes
Bacteroides
Bifidobacterium
Citrobacter
Clostridium
Collinsella
Coprobacillus
Enorma
Enterobacter
Enterococcus
Erysipelatoclostridium
Escherichia
Eubacterium
Flavonifractor
Intestinibacter
Klebsiella
Lactobacillus
Leclercia
Mobiluncus
Peptoclostridium
Ruminococcus
Streptococcus
Veillonella
undef

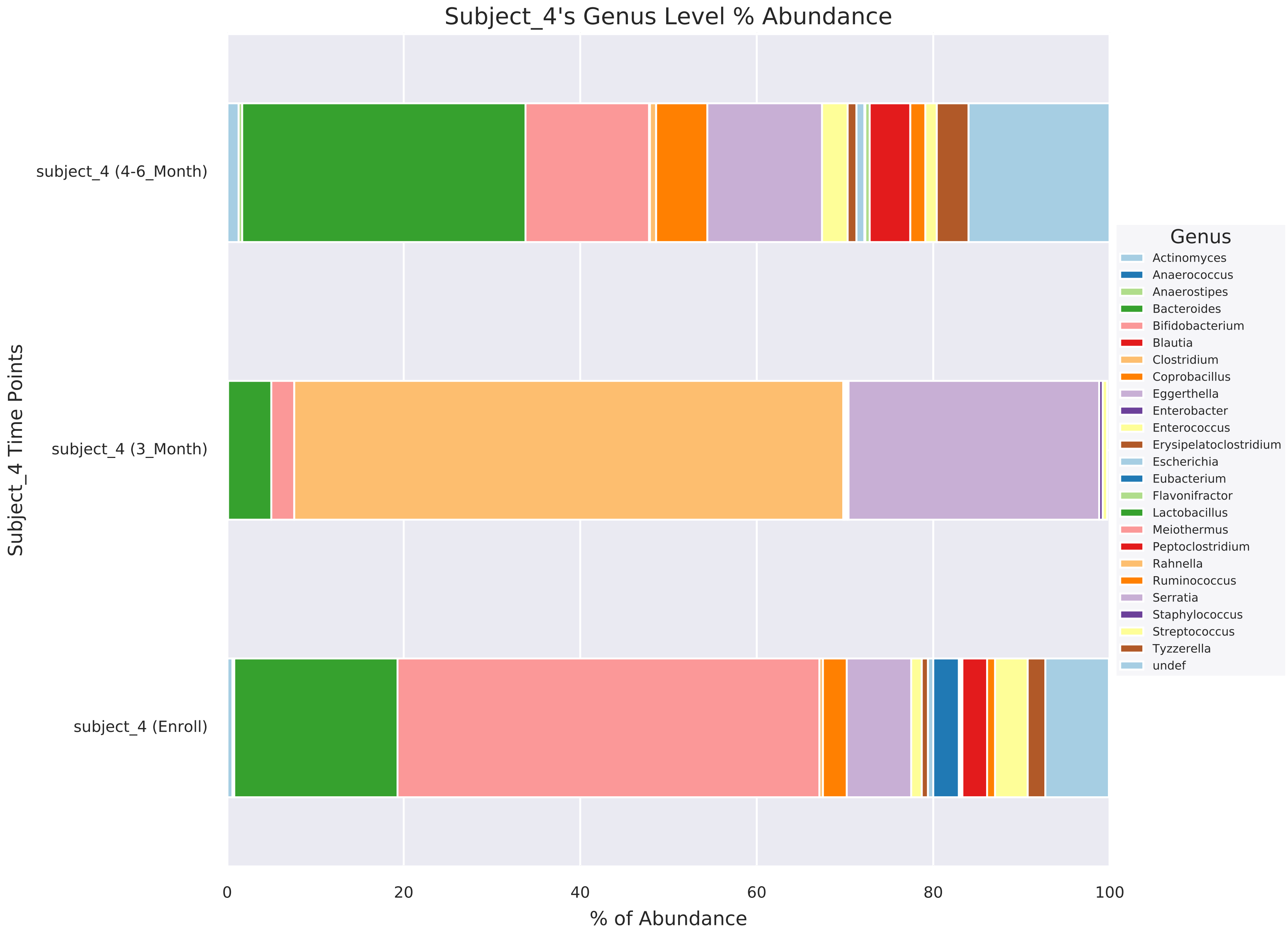
Subject_4's Genus Level % Abundance
Subject_4 Time Points
subject_4 (4-6_Month)
subject_4 (3_Month)
subject_4 (Enroll)
0
20
40
60
80
100
% of Abundance
Genus
Actinomyces
Anaerococcus
Anaerostipes
Bacteroides
Bifidobacterium
Blautia
Clostridium
Coprobacillus
Eggerthella
Enterobacter
Enterococcus
Erysipelatoclostridium
Escherichia
Eubacterium
Flavonifractor
Lactobacillus
Meiothermus
Peptoclostridium
Rahnella
Ruminococcus
Serratia
Staphylococcus
Streptococcus
Tyzzerella
undef

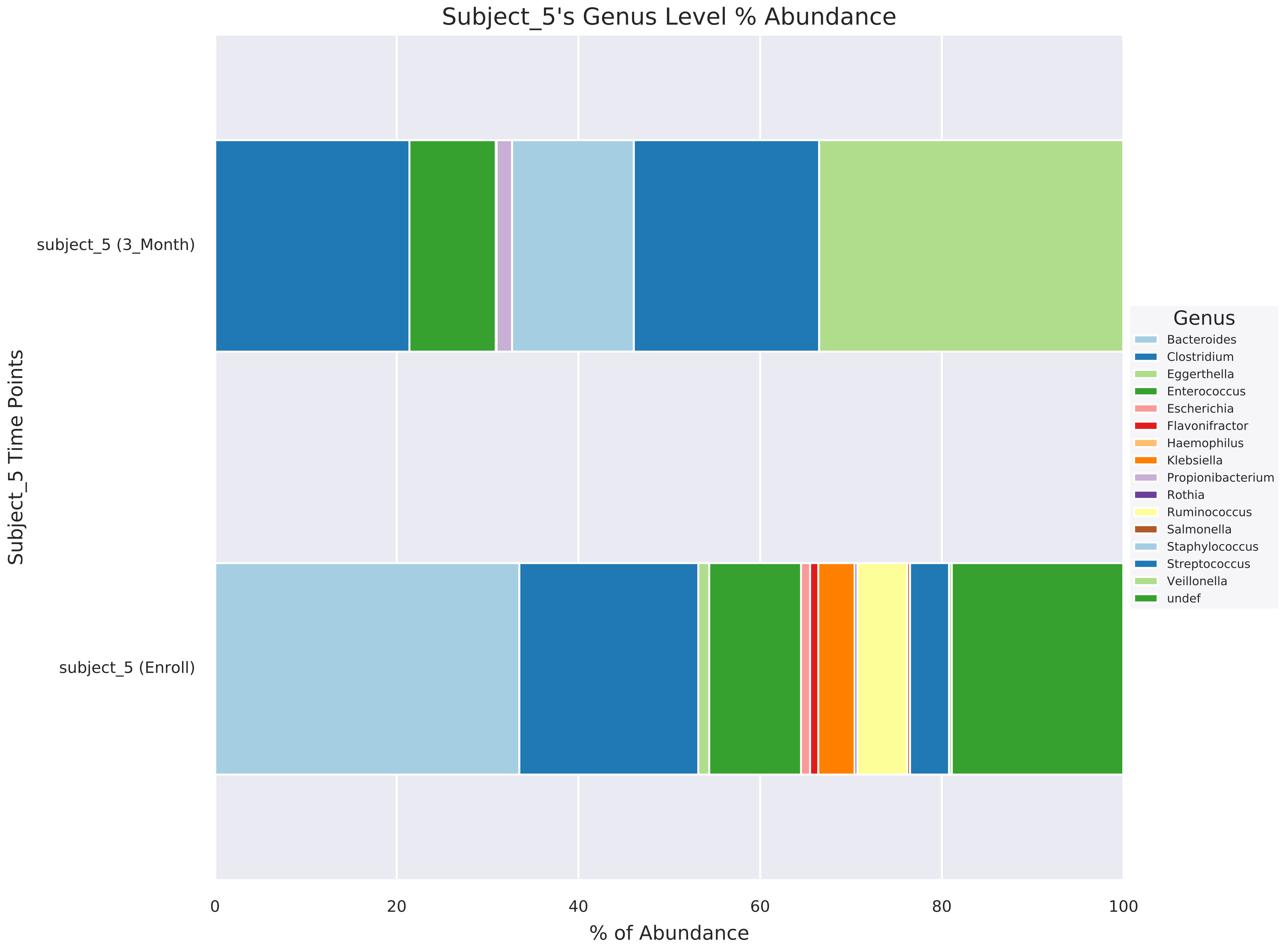

Subject_5's Genus Level % Abundance
subject_5 (3_Month)
subject_5 (Enroll)
Subject_5 Time Points
0
20
40
60
80
100
% of Abundance
Genus
Bacteroides
Clostridium
Eggerthella
Enterococcus
Escherichia
Flavonifractor
Haemophilus
Klebsiella
Propionibacterium
Rothia
Ruminococcus
Salmonella
Staphylococcus
Streptococcus
Veillonella
undef

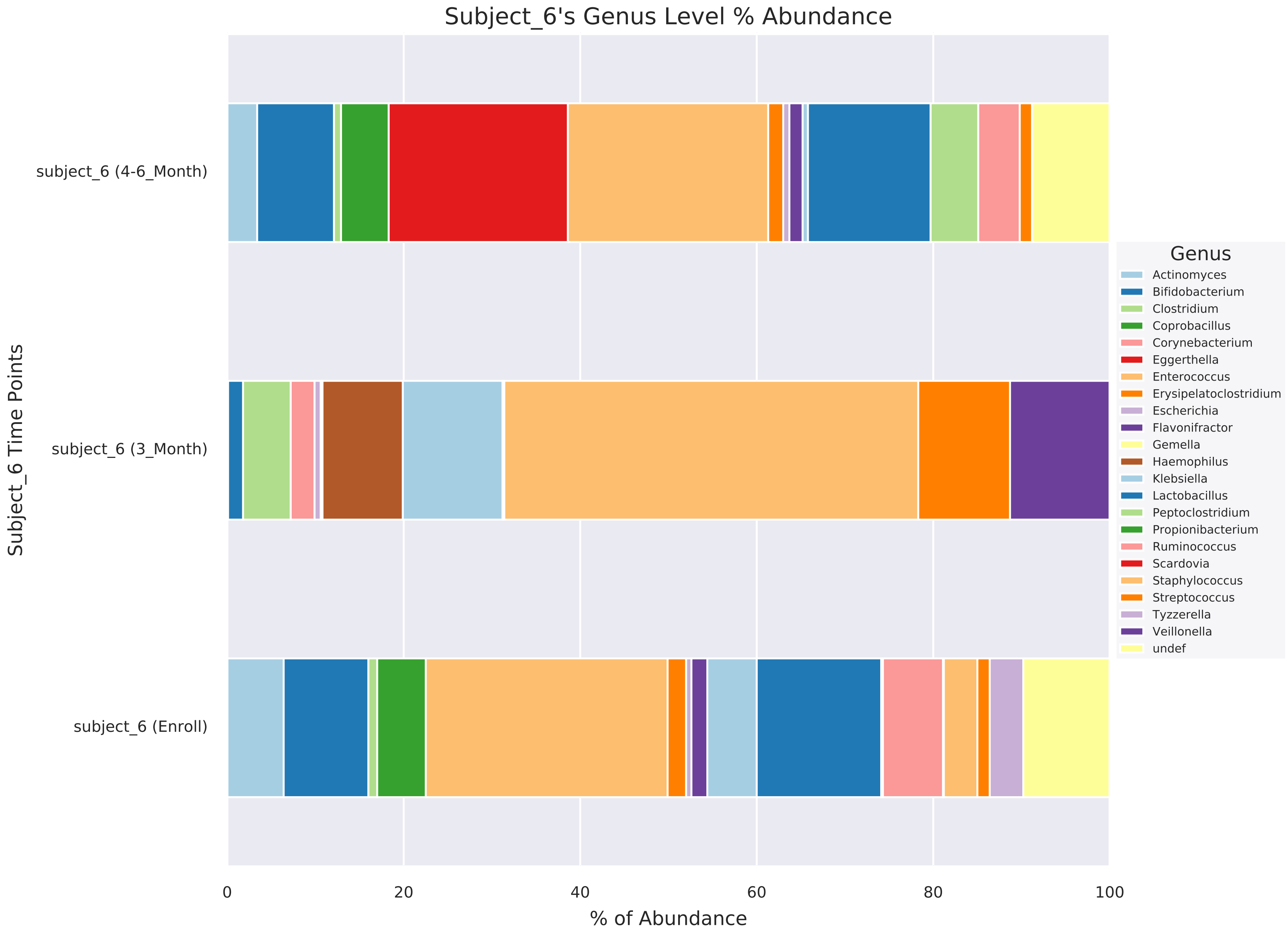

Subject_6's Genus Level % Abundance
subject_6 (4-6_Month)
subject_6 (3_Month)
subject_6 (Enroll)
Subject_6 Time Points
0
20
40
60
80
100
% of Abundance
Genus
Actinomyces
Bifidobacterium
Clostridium
Coprobacillus
Corynebacterium
Eggerthella
Enterococcus
Erysipelatoclostridium
Escherichia
Flavonifractor
Gemella
Haemophilus
Klebsiella
Lactobacillus
Peptoclostridium
Propionibacterium
Ruminococcus
Scardovia
Staphylococcus
Streptococcus
Tyzzerella
Veillonella
undef

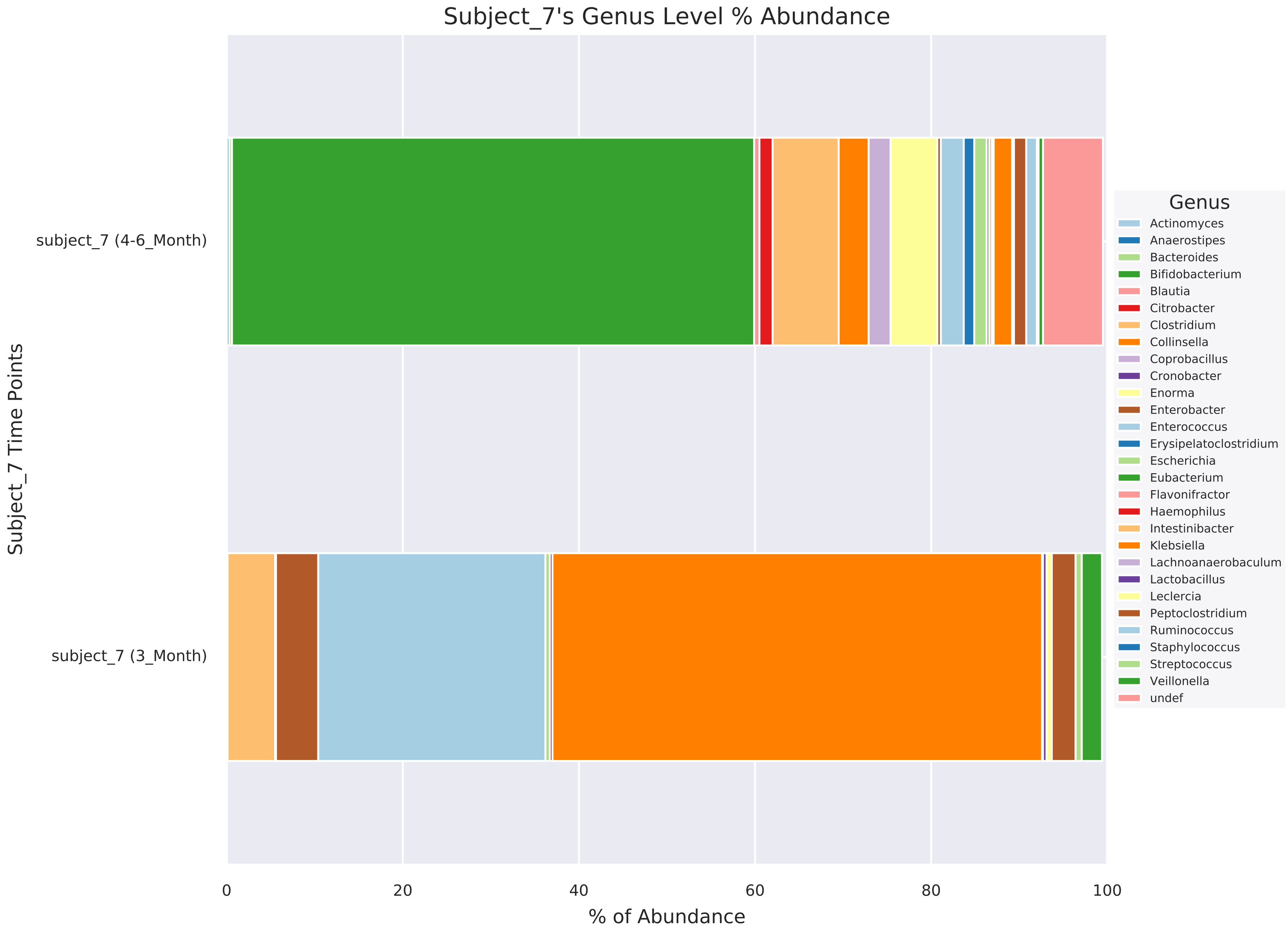

Subject_7's Genus Level % Abundance
subject_7 (4-6_Month)
subject_7 (3_Month)
Subject_7 Time Points
0
20
40
60
80
100
% of Abundance
Genus
Actinomyces
Anaerostipes
Bacteroides
Bifidobacterium
Blautia
Citrobacter
Clostridium
Collinsella
Coprobacillus
Cronobacter
Enorma
Enterobacter
Enterococcus
Erysipelatoclostridium
Escherichia
Eubacterium
Flavonifractor
Haemophilus
Intestinibacter
Klebsiella
Lachnoanaerobaculum
Lactobacillus
Leclercia
Peptoclostridium
Ruminococcus
Staphylococcus
Streptococcus
Veillonella
undef

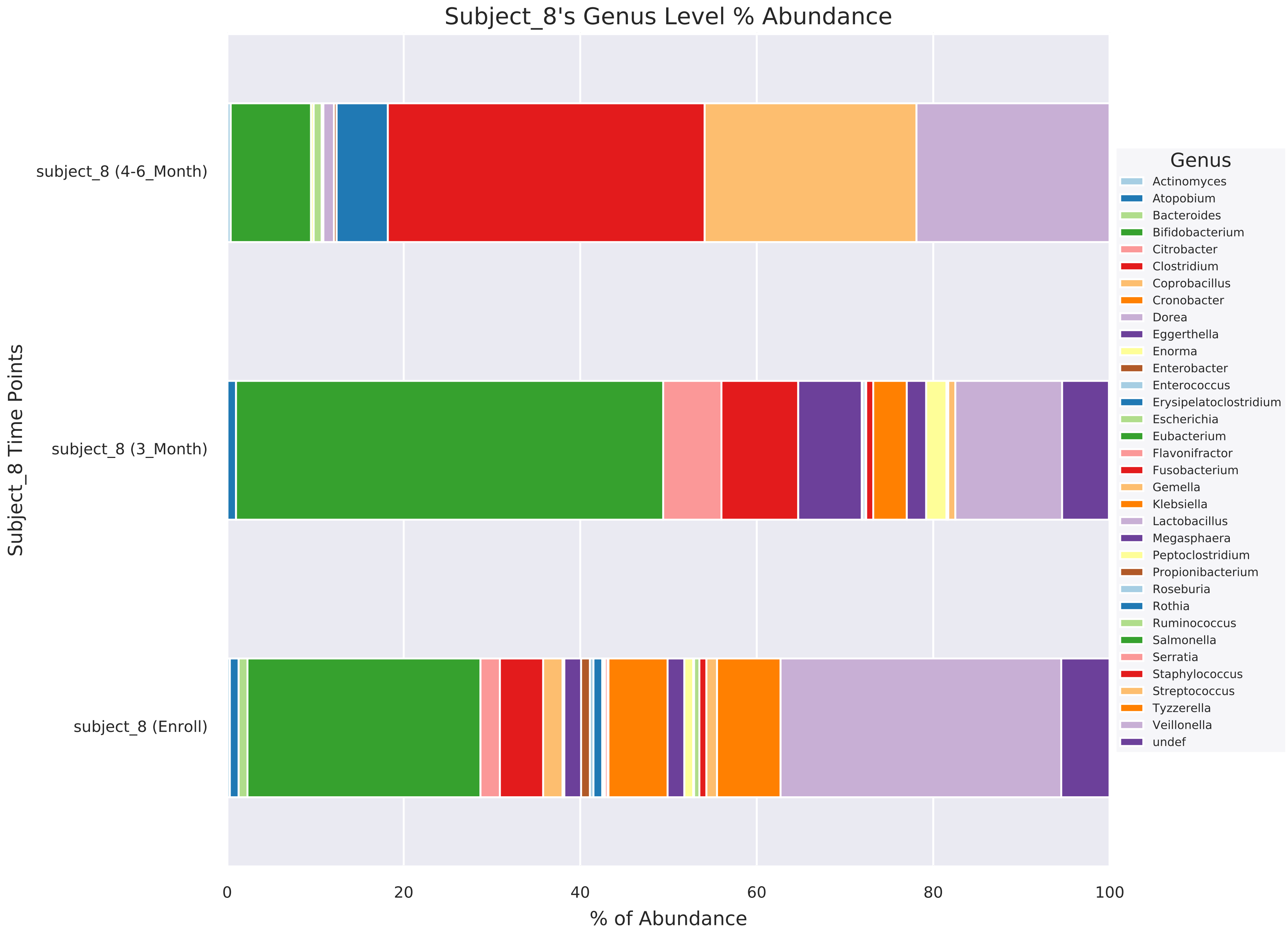
Subject_8's Genus Level % Abundance
subject_8 (4-6_Month)
subject_8 (3_Month)
subject_8 (Enroll)
Subject_8 Time Points
0
20
40
60
80
100
% of Abundance
Genus
Actinomyces
Atopobium
Bacteroides
Bifidobacterium
Citrobacter
Clostridium
Coprobacillus
Cronobacter
Dorea
Eggerthella
Enorma
Enterobacter
Enterococcus
Erysipelatoclostridium
Escherichia
Eubacterium
Flavonifractor
Fusobacterium
Gemella
Klebsiella
Lactobacillus
Megasphaera
Peptoclostridium
Propionibacterium
Roseburia
Rothia
Ruminococcus
Salmonella
Serratia
Staphylococcus
Streptococcus
Tyzzerella
Veillonella
undef

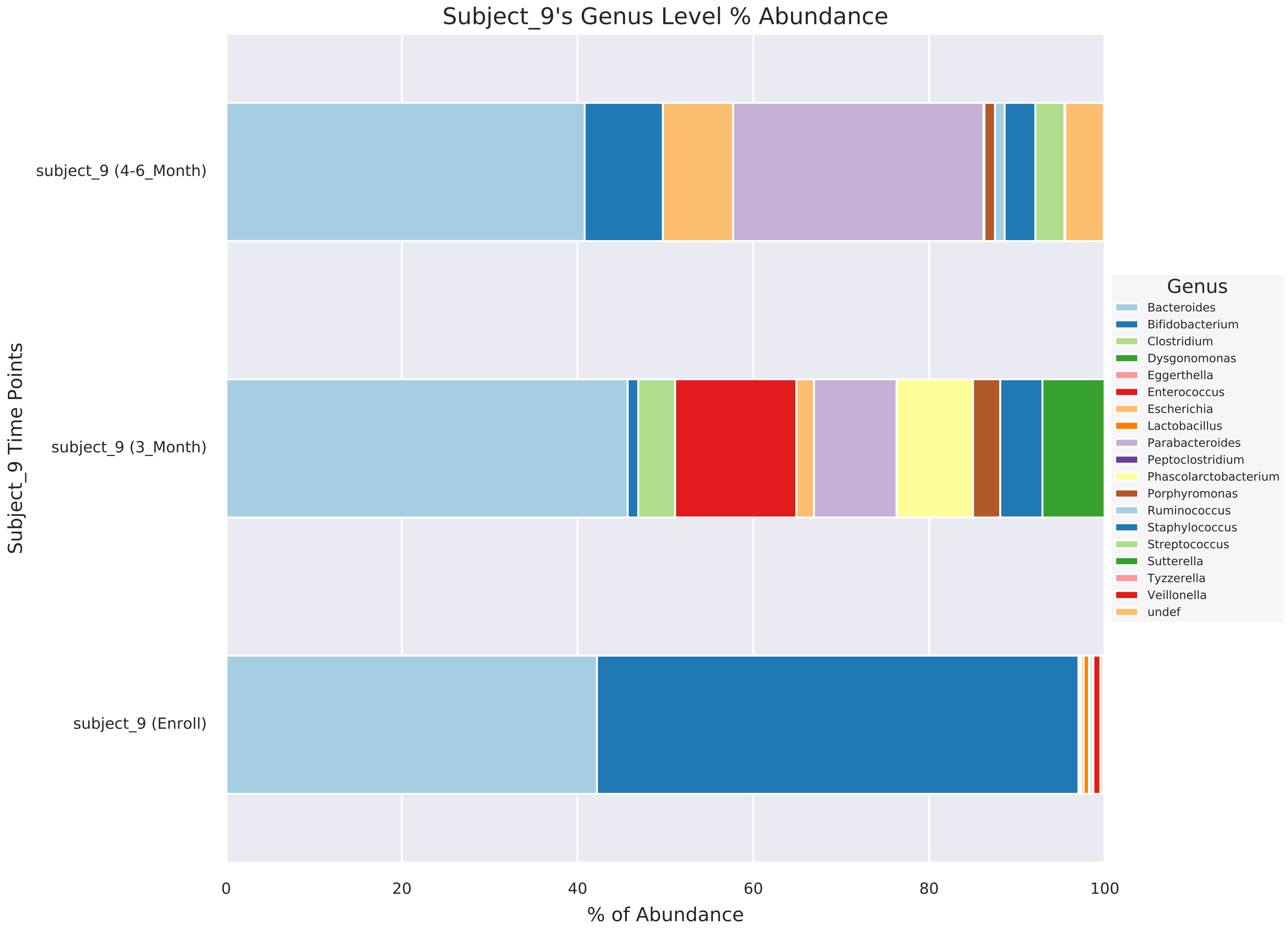
Subject_9's Genus Level % Abundance
Subject_9 Time Points
subject_9 (4-6_Month)
subject_9 (3_Month)
subject_9 (Enroll)
0
20
40
60
80
100
% of Abundance
Genus
Bacteroides
Bifidobacterium
Clostridium
Dysgonomonas
Eggerthella
Enterococcus
Escherichia
Lactobacillus
Parabacteroides
Peptoclostridium
Phascolarctobacterium
Porphyromonas
Ruminococcus
Staphylococcus
Streptococcus
Sutterella
Tyzzerella
Veillonella
undef

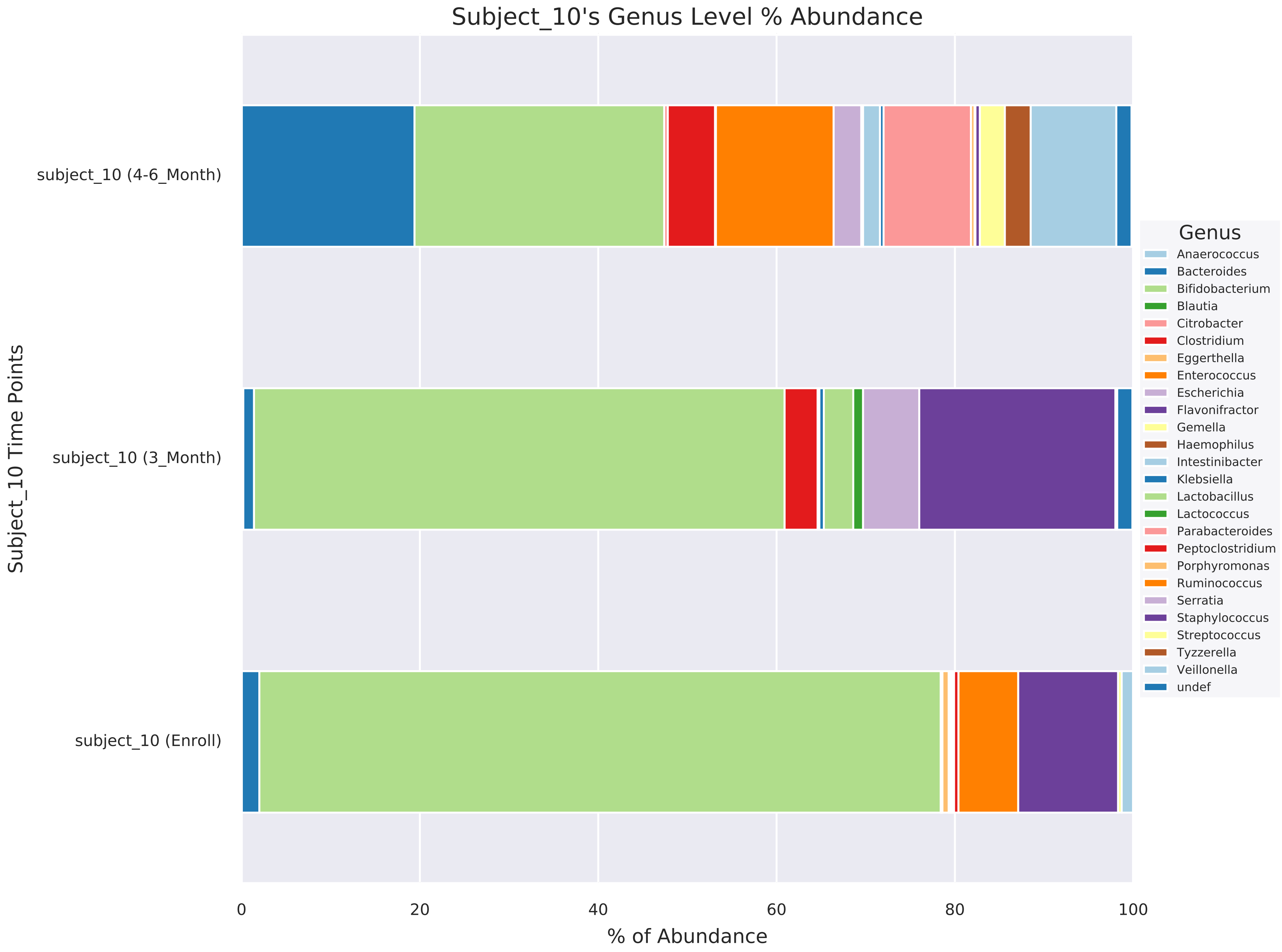
Subject_10's Genus Level % Abundance
subject_10 (4-6_Month)
subject_10 (3_Month)
subject_10 (Enroll)
Subject_10 Time Points
0
20
40
60
80
100
% of Abundance
Genus
Anaerococcus
Bacteroides
Bifidobacterium
Blautia
Citrobacter
Clostridium
Eggerthella
Enterococcus
Escherichia
Flavonifractor
Gemella
Haemophilus
Intestinibacter
Klebsiella
Lactobacillus
Lactococcus
Parabacteroides
Peptoclostridium
Porphyromonas
Ruminococcus
Serratia
Staphylococcus
Streptococcus
Tyzzerella
Veillonella
undef

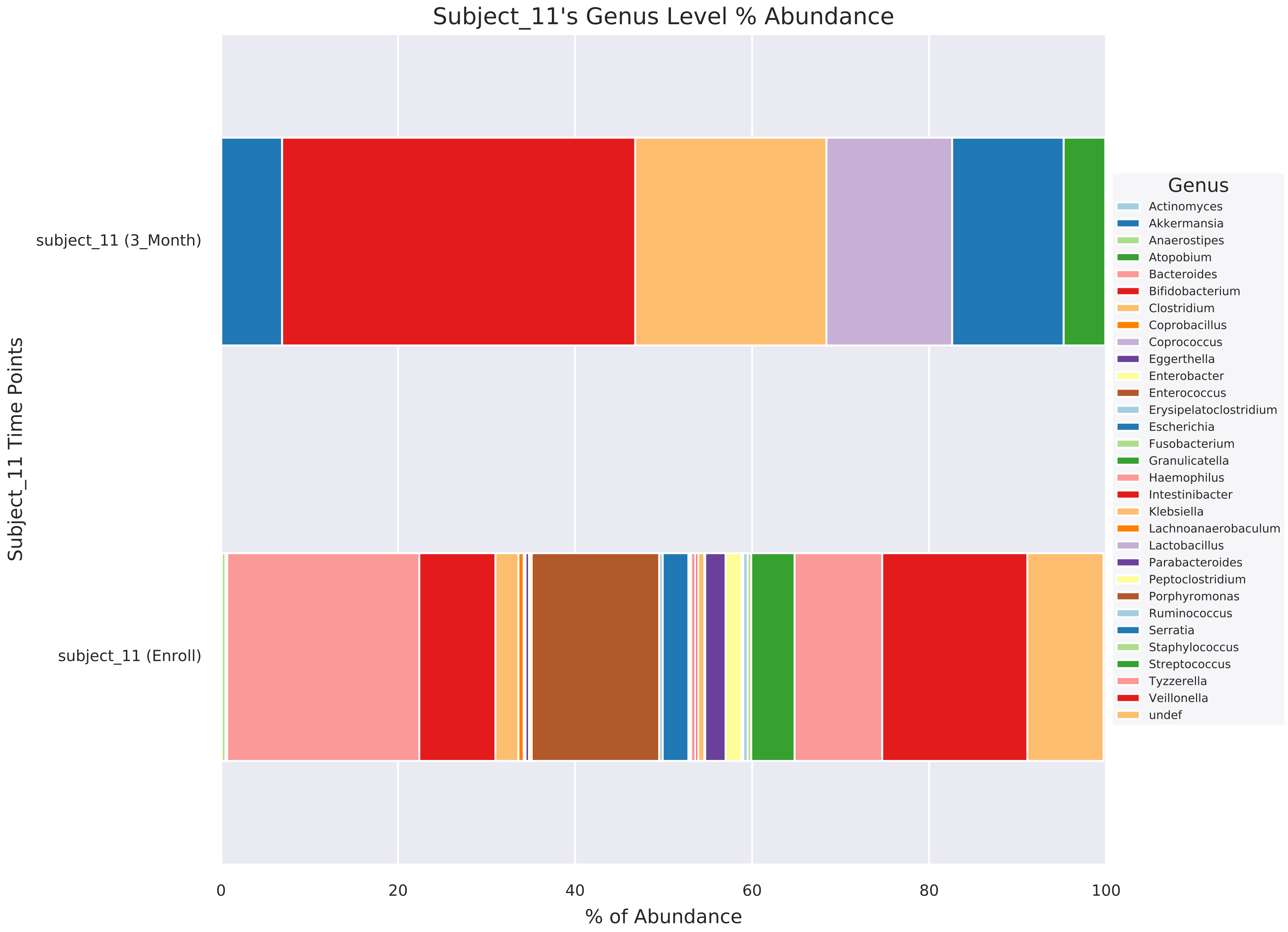
Subject_11's Genus Level % Abundance
Subject_11 Time Points
subject_11 (3_Month)
subject_11 (Enroll)
0
20
40
60
80
100
% of Abundance
Genus
Actinomyces
Akkermansia
Anaerostipes
Atopobium
Bacteroides
Bifidobacterium
Clostridium
Coprobacillus
Coprococcus
Eggerthella
Enterobacter
Enterococcus
Erysipelatoclostridium
Escherichia
Fusobacterium
Granulicatella
Haemophilus
Intestinibacter
Klebsiella
Lachnoanaerobaculum
Lactobacillus
Parabacteroides
Peptoclostridium
Porphyromonas
Ruminococcus
Serratia
Staphylococcus
Streptococcus
Tyzzerella
Veillonella
undef

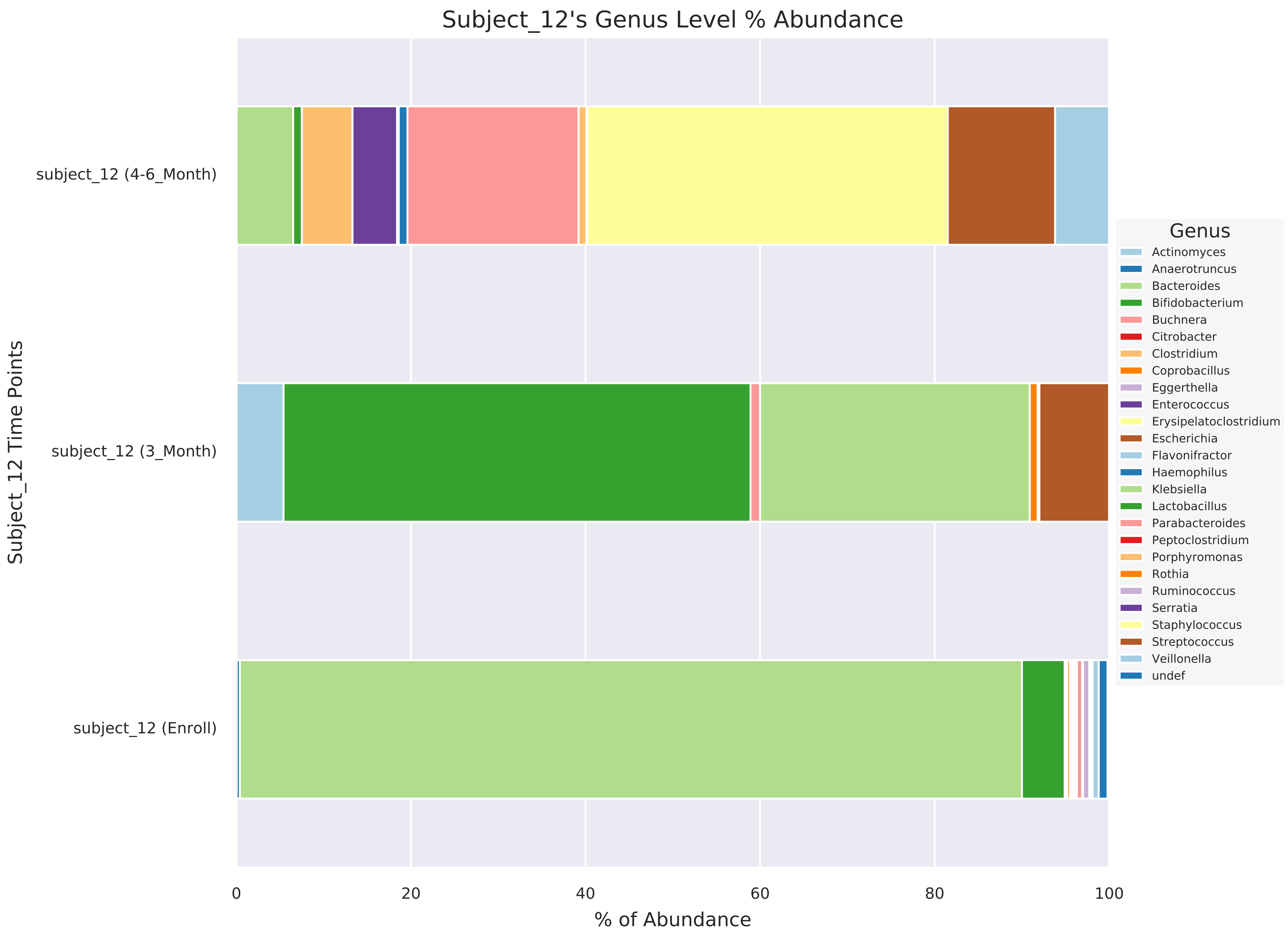
Subject_12's Genus Level % Abundance
subject_12 (4-6_Month)
subject_12 (3_Month)
subject_12 (Enroll)
Subject_12 Time Points
0
20
40
60
80
100
% of Abundance
Genus
Actinomyces
Anaerotruncus
Bacteroides
Bifidobacterium
Buchnera
Citrobacter
Clostridium
Coprobacillus
Eggerthella
Enterococcus
Erysipelatoclostridium
Escherichia
Flavonifractor
Haemophilus
Klebsiella
Lactobacillus
Parabacteroides
Peptoclostridium
Porphyromonas
Rothia
Ruminococcus
Serratia
Staphylococcus
Streptococcus
Veillonella
undef

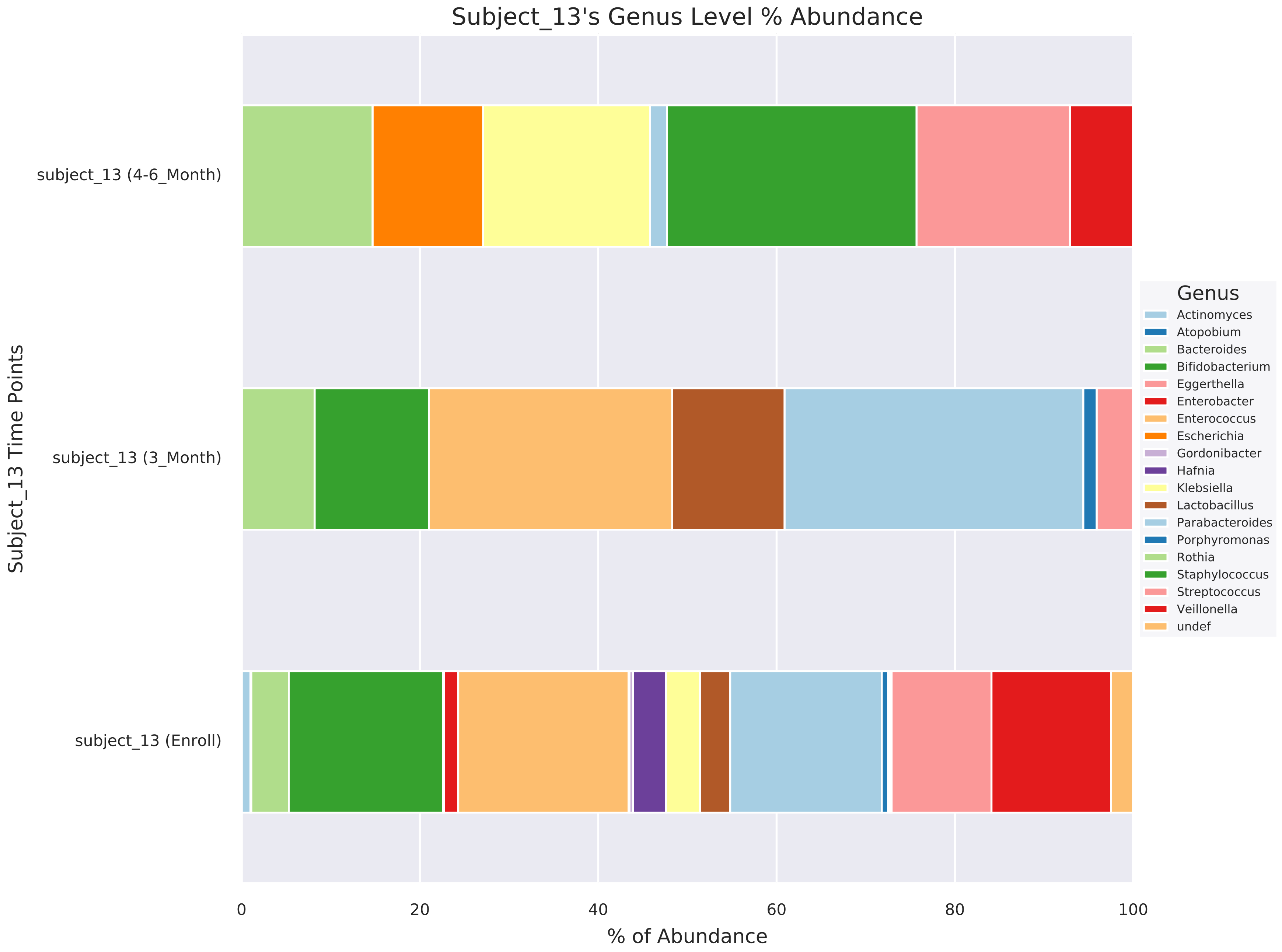
Subject_13's Genus Level % Abundance
subject_13 (4-6_Month)
subject_13 (3_Month)
subject_13 (Enroll)
Subject_13 Time Points
0
20
40
60
80
100
% of Abundance
Genus
Actinomyces
Atopobium
Bacteroides
Bifidobacterium
Eggerthella
Enterobacter
Enterococcus
Escherichia
Gordonibacter
Hafnia
Klebsiella
Lactobacillus
Parabacteroides
Porphyromonas
Rothia
Staphylococcus
Streptococcus
Veillonella
undef

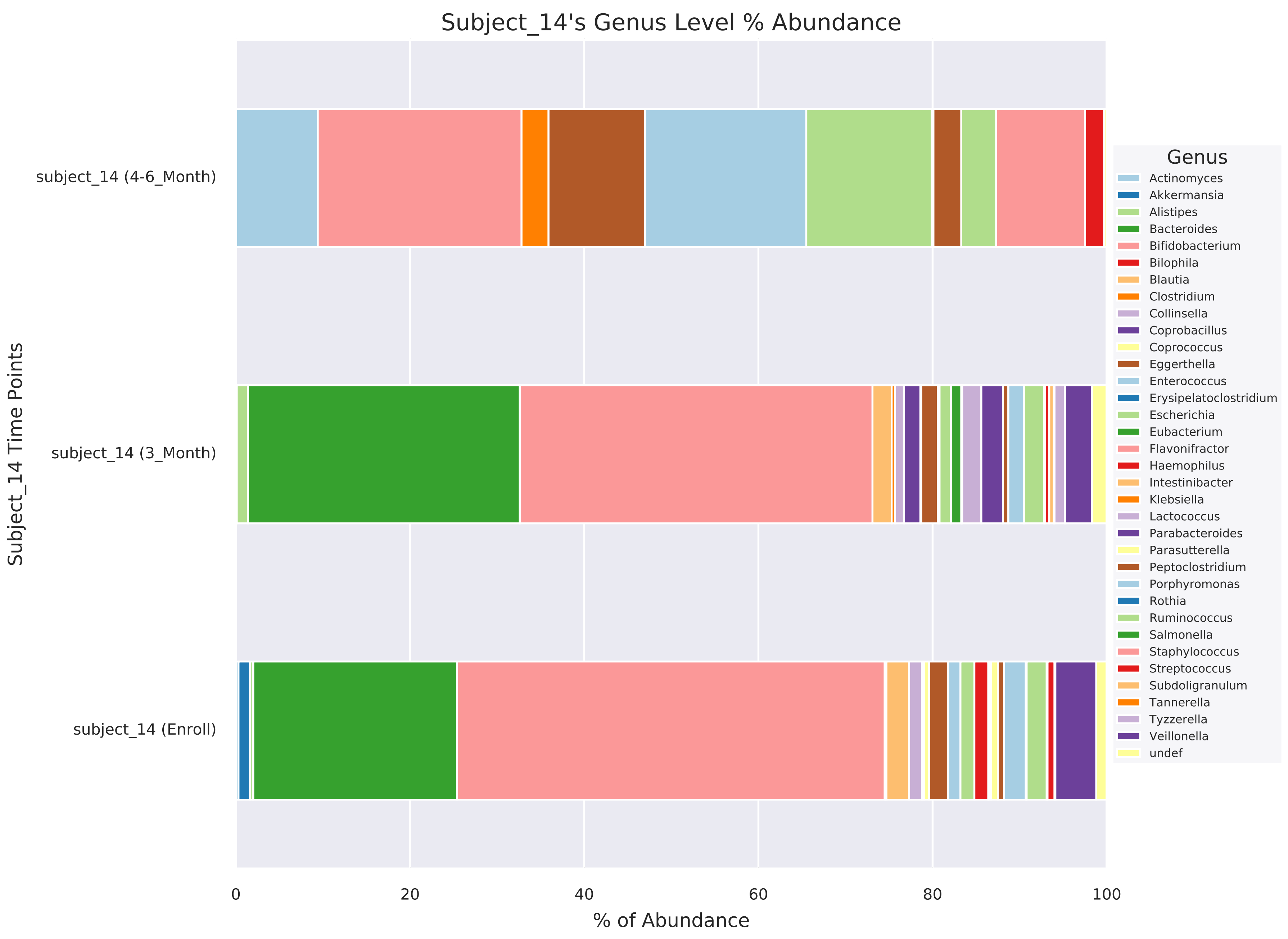

Subject_14's Genus Level % Abundance
subject_14 (4-6_Month)
subject_14 (3_Month)
subject_14 (Enroll)
Subject_14 Time Points
0
20
40
60
80
100
% of Abundance
Genus
Actinomyces
Akkermansia
Alistipes
Bacteroides
Bifidobacterium
Bilophila
Blautia
Clostridium
Collinsella
Coprobacillus
Coprococcus
Eggerthella
Enterococcus
Erysipelatoclostridium
Escherichia
Eubacterium
Flavonifractor
Haemophilus
Intestinibacter
Klebsiella
Lactococcus
Parabacteroides
Parasutterella
Peptoclostridium
Porphyromonas
Rothia
Ruminococcus
Salmonella
Staphylococcus
Streptococcus
Subdoligranulum
Tannerella
Tyzzerella
Veillonella
undef

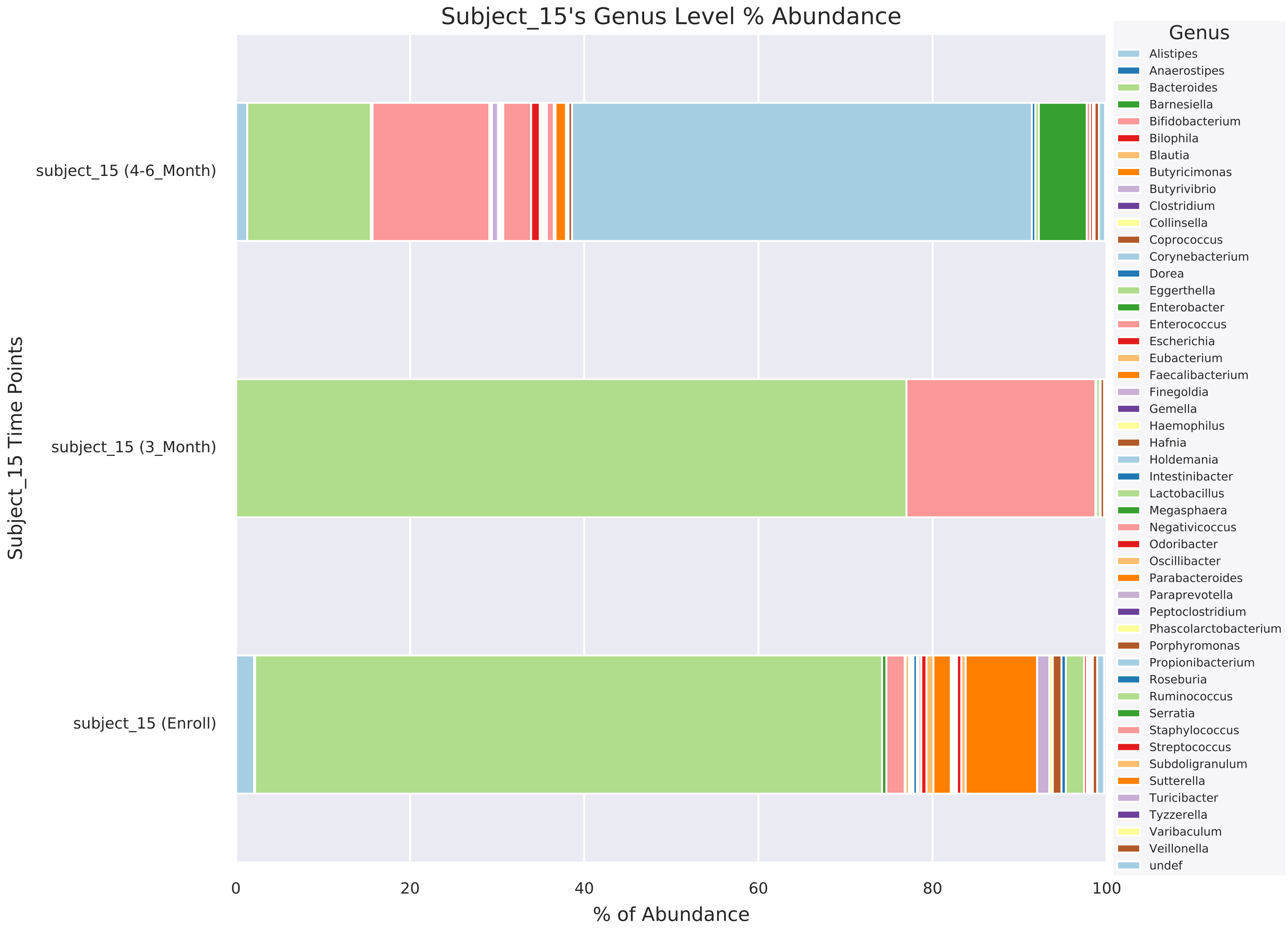

Subject_15's Genus Level % Abundance
Subject_15 Time Points
subject_15 (4-6_Month)
subject_15 (3_Month)
subject_15 (Enroll)
0
20
40
60
80
100
% of Abundance
Genus
Alistipes
Anaerostipes
Bacteroides
Barnesiella
Bifidobacterium
Bilophila
Blautia
Butyricimonas
Butyrivibrio
Clostridium
Collinsella
Coprococcus
Corynebacterium
Dorea
Eggerthella
Enterobacter
Enterococcus
Escherichia
Eubacterium
Faecalibacterium
Finegoldia
Gemella
Haemophilus
Hafnia
Holdemania
Intestinibacter
Lactobacillus
Megasphaera
Negativicoccus
Odoribacter
Oscillibacter
Parabacteroides
Paraprevotella
Peptoclostridium
Phascolarctobacterium
Porphyromonas
Propionibacterium
Roseburia
Ruminococcus
Serratia
Staphylococcus
Streptococcus
Subdoligranulum
Sutterella
Turicibacter
Tyzzerella
Varibaculum
Veillonella
undef

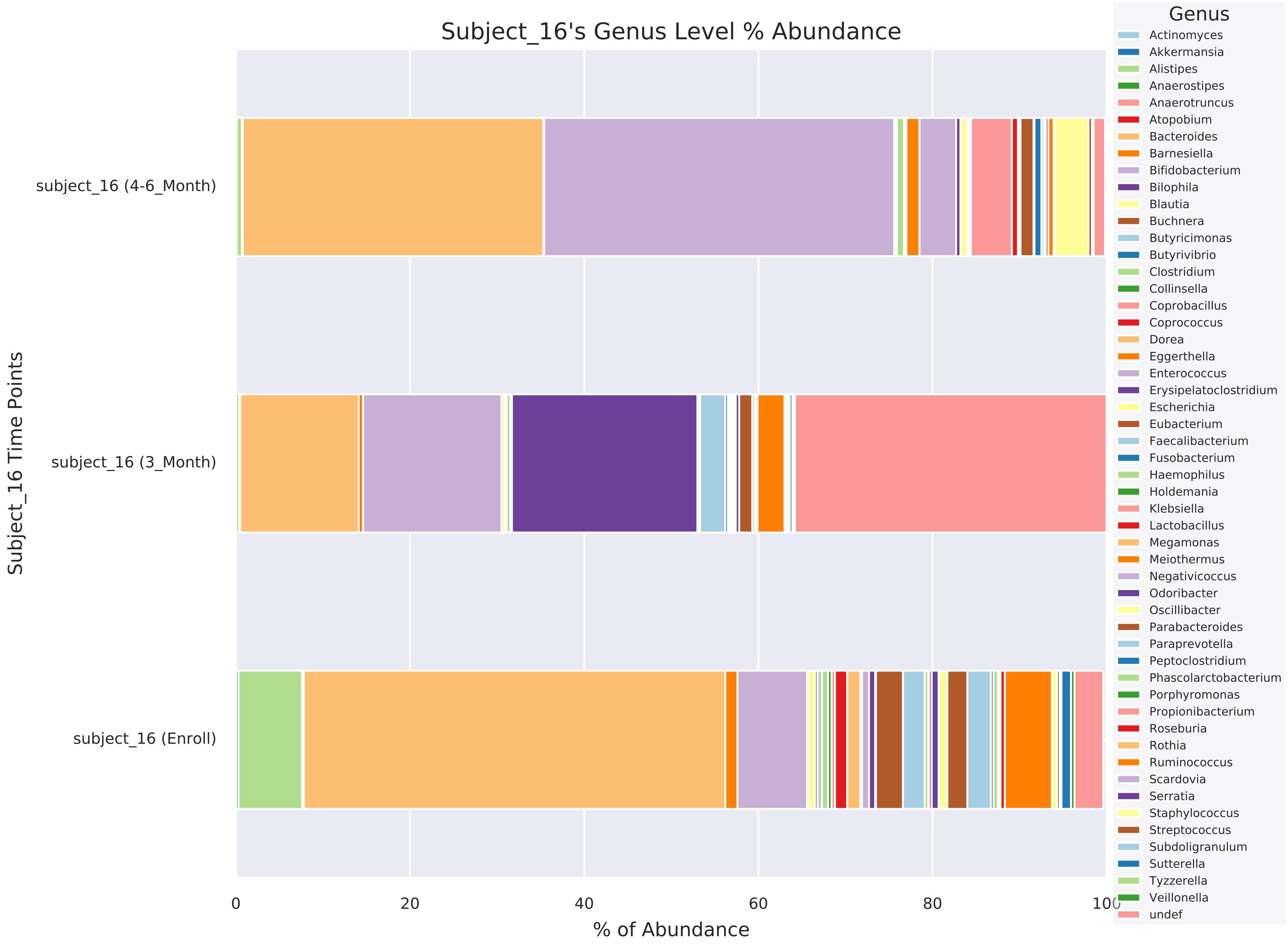
Subject_16's Genus Level % Abundance
Subject_16 Time Points
subject_16 (4-6_Month)
subject_16 (3_Month)
subject_16 (Enroll)
0
20
40
60
80
100
% of Abundance
Genus
Actinomyces
Akkermansia
Alistipes
Anaerostipes
Anaerotruncus
Atopobium
Bacteroides
Barnesiella
Bifidobacterium
Bilophila
Blautia
Buchnera
Butyricimonas
Butyrivibrio
Clostridium
Collinsella
Coprobacillus
Coprococcus
Dorea
Eggerthella
Enterococcus
Erysipelatoclostridium
Escherichia
Eubacterium
Faecalibacterium
Fusobacterium
Haemophilus
Holdemania
Klebsiella
Lactobacillus
Megamonas
Meiothermus
Negativicoccus
Odoribacter
Oscillibacter
Parabacteroides
Paraprevotella
Peptoclostridium
Phascolarctobacterium
Porphyromonas
Propionibacterium
Roseburia
Rothia
Ruminococcus
Scardovia
Serratia
Staphylococcus
Streptococcus
Subdoligranulum
Sutterella
Tyzzerella
Veillonella
undef

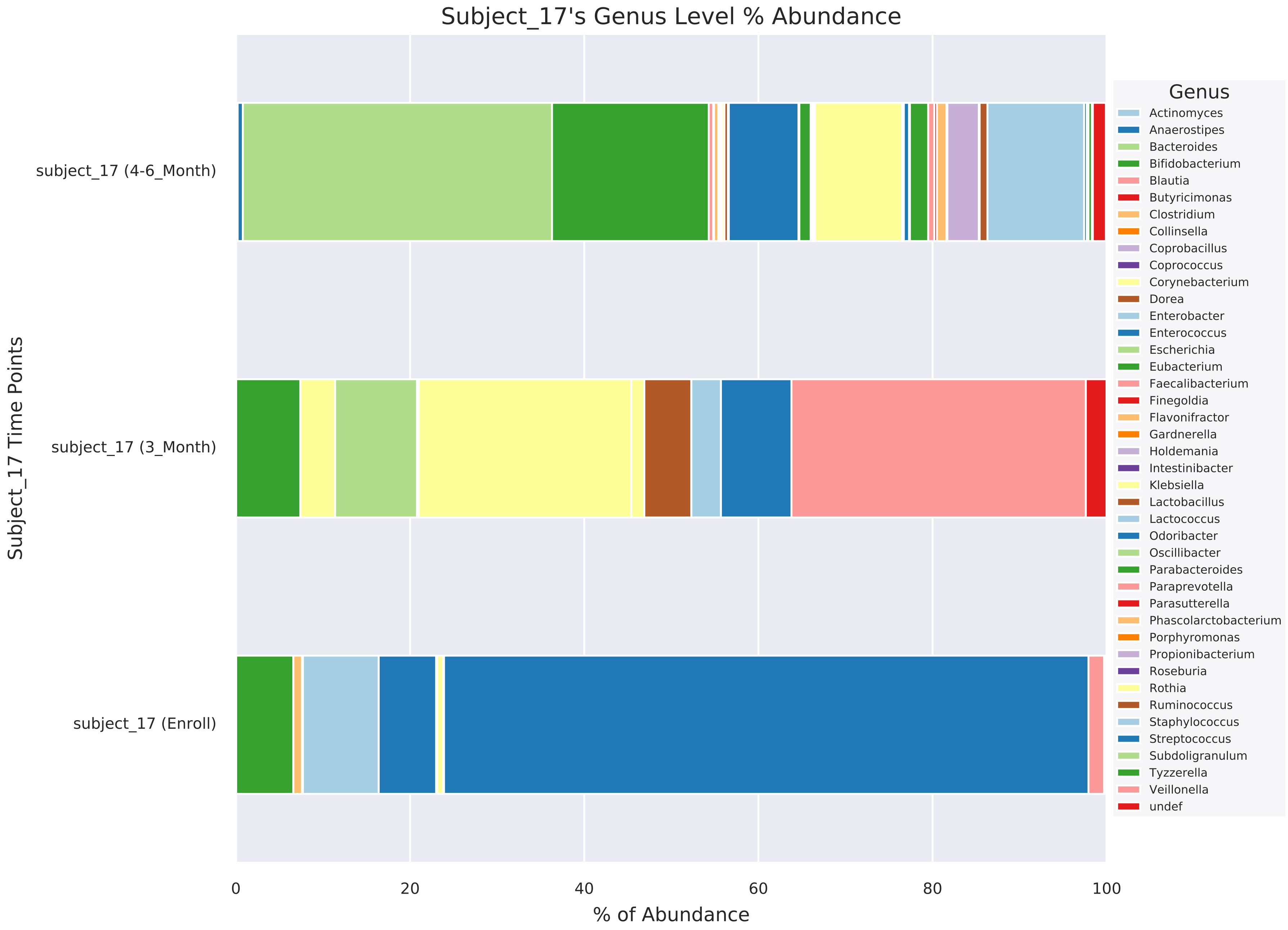
Subject_17's Genus Level % Abundance
Subject_17 Time Points
subject_17 (4-6_Month)
subject_17 (3_Month)
subject_17 (Enroll)
0
20
40
60
80
100
% of Abundance
Genus
Actinomyces
Anaerostipes
Bacteroides
Bifidobacterium
Blautia
Butyricimonas
Clostridium
Collinsella
Coprobacillus
Coprococcus
Corynebacterium
Dorea
Enterobacter
Enterococcus
Escherichia
Eubacterium
Faecalibacterium
Finegoldia
Flavonifractor
Gardnerella
Holdemania
Intestinibacter
Klebsiella
Lactobacillus
Lactococcus
Odoribacter
Oscillibacter
Parabacteroides
Paraprevotella
Parasutterella
Phascolarctobacterium
Porphyromonas
Propionibacterium
Roseburia
Rothia
Ruminococcus
Staphylococcus
Streptococcus
Subdoligranulum
Tyzzerella
Veillonella
undef

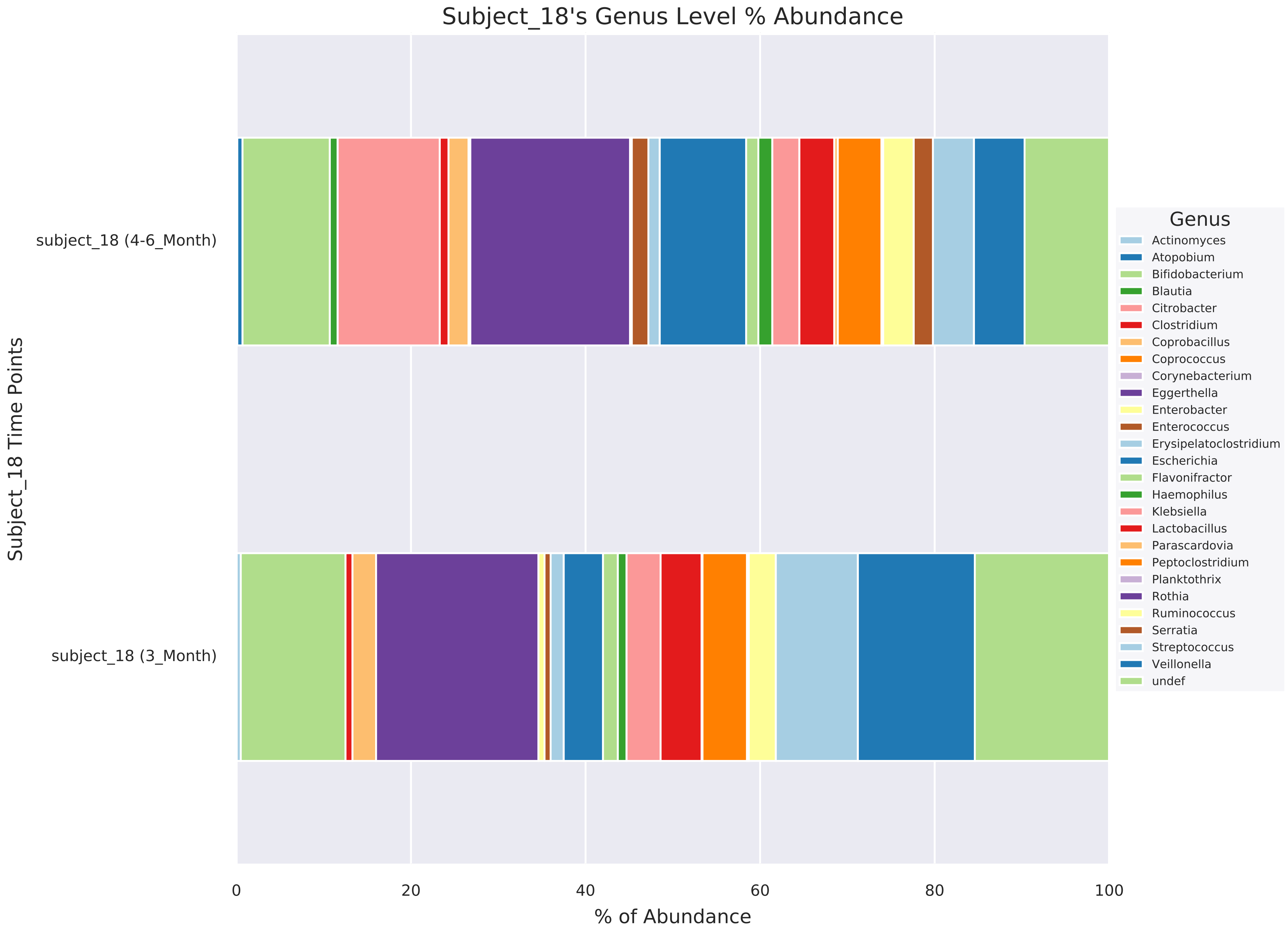

Subject_18's Genus Level % Abundance
Subject_18 Time Points
subject_18 (4-6_Month)
subject_18 (3_Month)
0
20
40
60
80
100
% of Abundance
Genus
Actinomyces
Atopobium
Bifidobacterium
Blautia
Citrobacter
Clostridium
Coprobacillus
Coprococcus
Corynebacterium
Eggerthella
Enterobacter
Enterococcus
Erysipelatoclostridium
Escherichia
Flavonifractor
Haemophilus
Klebsiella
Lactobacillus
Parascardovia
Peptoclostridium
Planktothrix
Rothia
Ruminococcus
Serratia
Streptococcus
Veillonella
undef

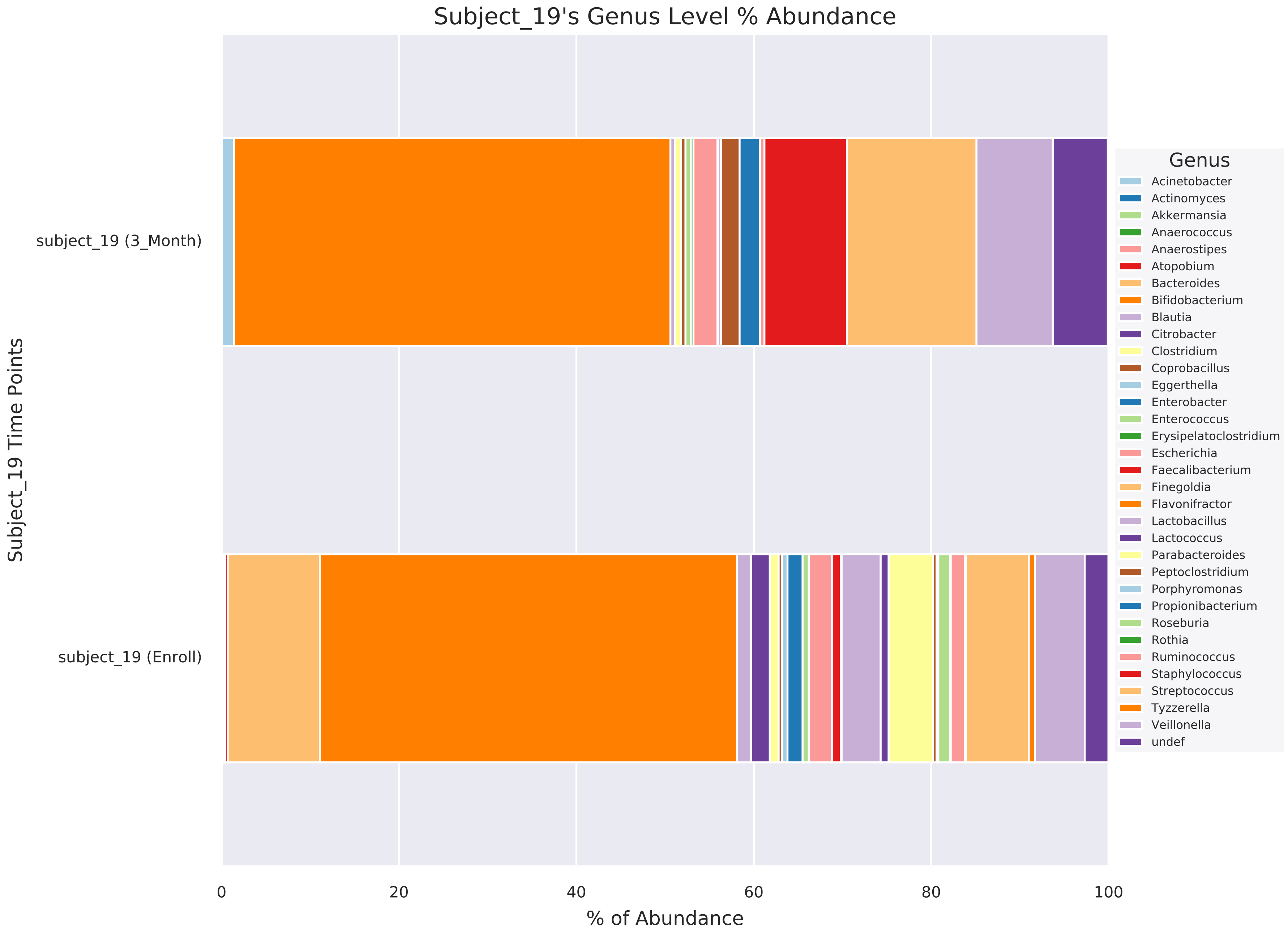

Subject_19's Genus Level % Abundance
subject_19 (3_Month)
subject_19 (Enroll)
Subject_19 Time Points
0
20
40
60
80
100
% of Abundance
Genus
Acinetobacter
Actinomyces
Akkermansia
Anaerococcus
Anaerostipes
Atopobium
Bacteroides
Bifidobacterium
Blautia
Citrobacter
Clostridium
Coprobacillus
Eggerthella
Enterobacter
Enterococcus
Erysipelatoclostridium
Escherichia
Faecalibacterium
Finegoldia
Flavonifractor
Lactobacillus
Lactococcus
Parabacteroides
Peptoclostridium
Porphyromonas
Propionibacterium
Roseburia
Rothia
Ruminococcus
Staphylococcus
Streptococcus
Tyzzerella
Veillonella
undef

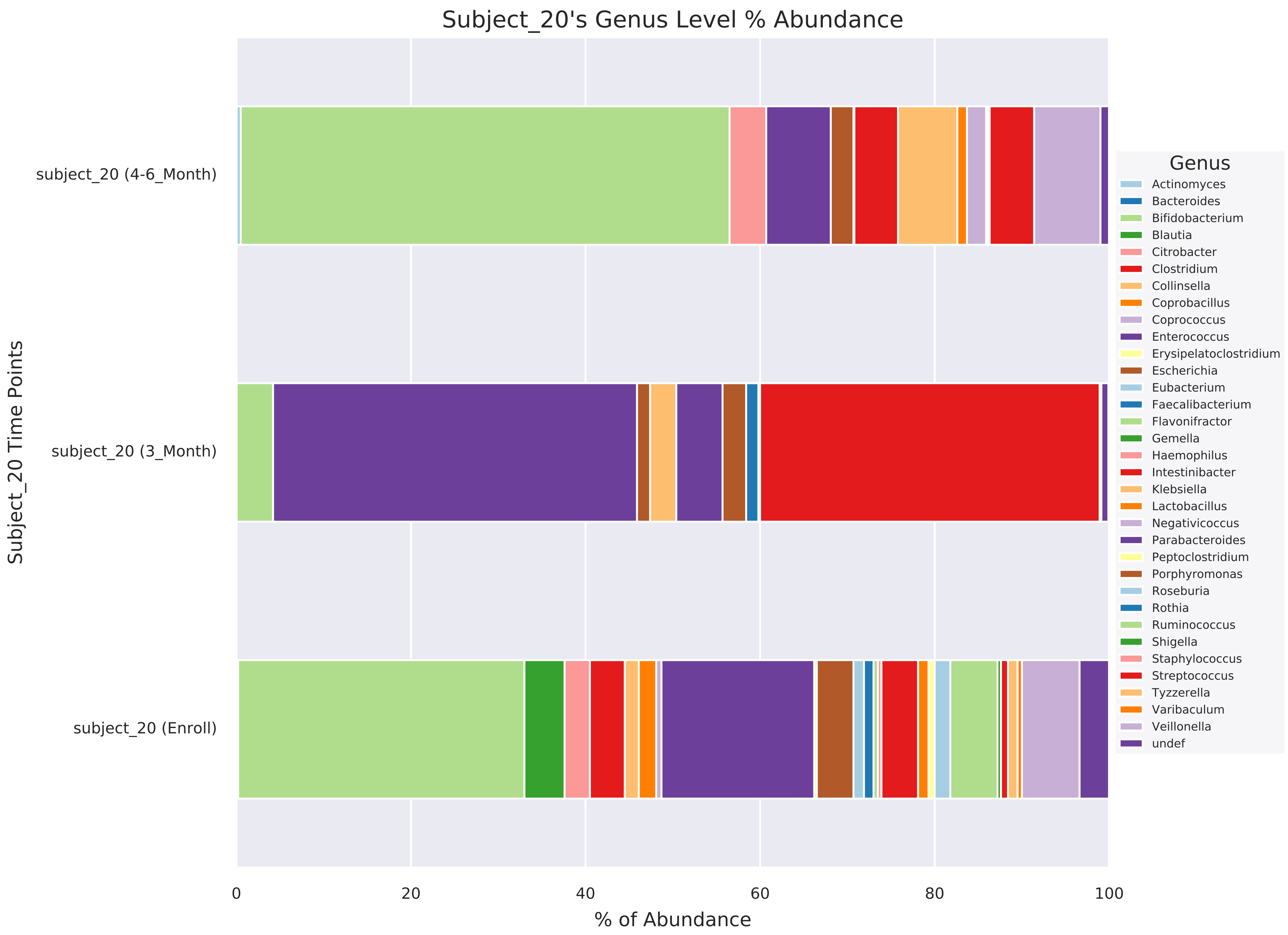
Subject_20's Genus Level % Abundance
Subject_20 Time Points
subject_20 (4-6_Month)
subject_20 (3_Month)
subject_20 (Enroll)
0
20
40
60
80
100
% of Abundance
Genus
Actinomyces
Bacteroides
Bifidobacterium
Blautia
Citrobacter
Clostridium
Collinsella
Coprobacillus
Coprococcus
Enterococcus
Erysipelatoclostridium
Escherichia
Eubacterium
Faecalibacterium
Flavonifractor
Gemella
Haemophilus
Intestinibacter
Klebsiella
Lactobacillus
Negativicoccus
Parabacteroides
Peptoclostridium
Porphyromonas
Roseburia
Rothia
Ruminococcus
Shigella
Staphylococcus
Streptococcus
Tyzzerella
Varibaculum
Veillonella
undef

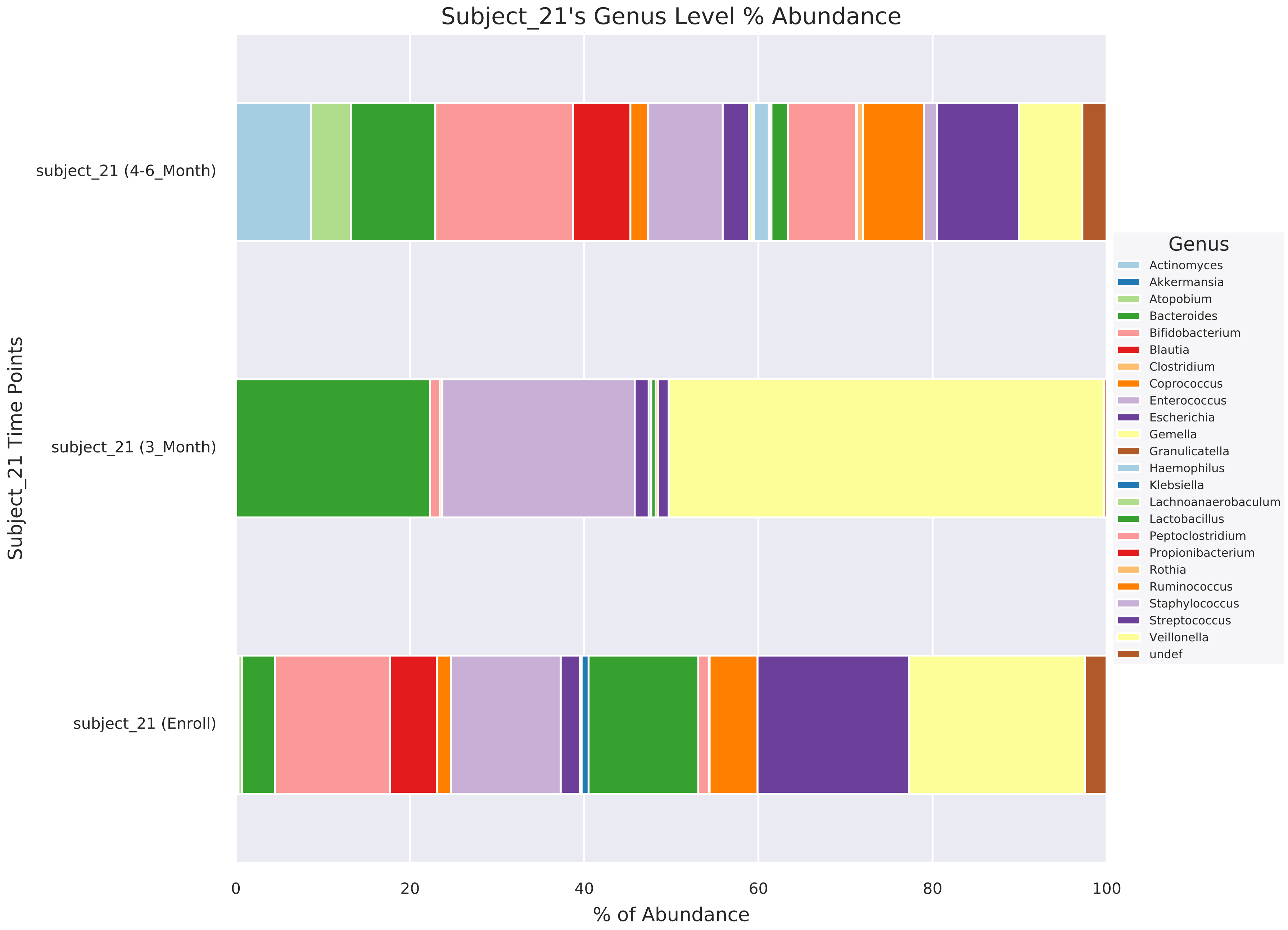

Subject_21's Genus Level % Abundance
Subject_21 Time Points
subject_21 (4-6_Month)
subject_21 (3_Month)
subject_21 (Enroll)
0
20
40
60
80
100
% of Abundance
Genus
Actinomyces
Akkermansia
Atopobium
Bacteroides
Bifidobacterium
Blautia
Clostridium
Coprococcus
Enterococcus
Escherichia
Gemella
Granulicatella
Haemophilus
Klebsiella
Lachnoanaerobaculum
Lactobacillus
Peptoclostridium
Propionibacterium
Rothia
Ruminococcus
Staphylococcus
Streptococcus
Veillonella
undef

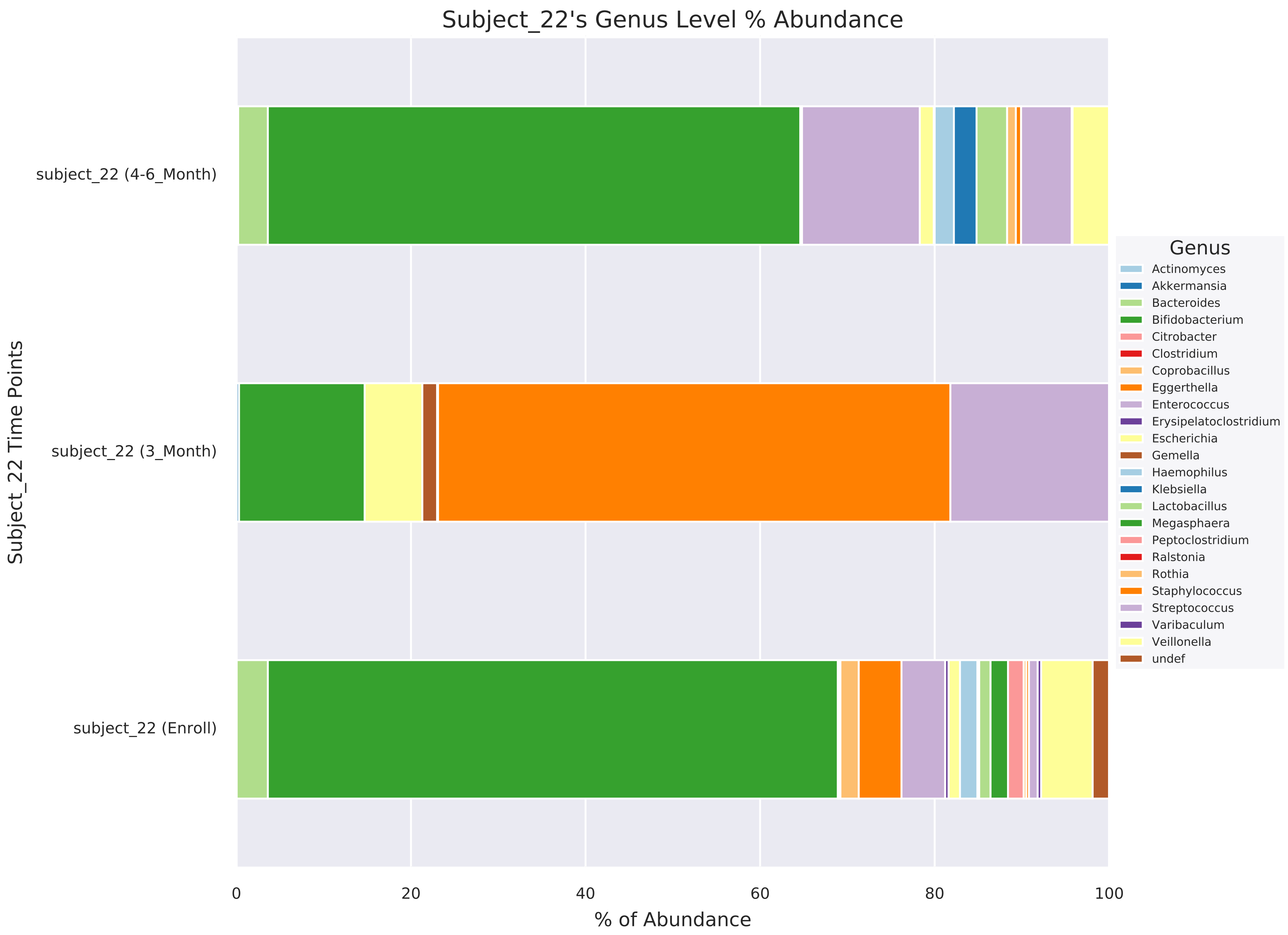
Subject_22's Genus Level % Abundance
subject_22 (4-6_Month)
subject_22 (3_Month)
subject_22 (Enroll)
Subject_22 Time Points
0
20
40
60
80
100
% of Abundance
Genus
Actinomyces
Akkermansia
Bacteroides
Bifidobacterium
Citrobacter
Clostridium
Coprobacillus
Eggerthella
Enterococcus
Erysipelatoclostridium
Escherichia
Gemella
Haemophilus
Klebsiella
Lactobacillus
Megasphaera
Peptoclostridium
Ralstonia
Rothia
Staphylococcus
Streptococcus
Varibaculum
Veillonella
undef

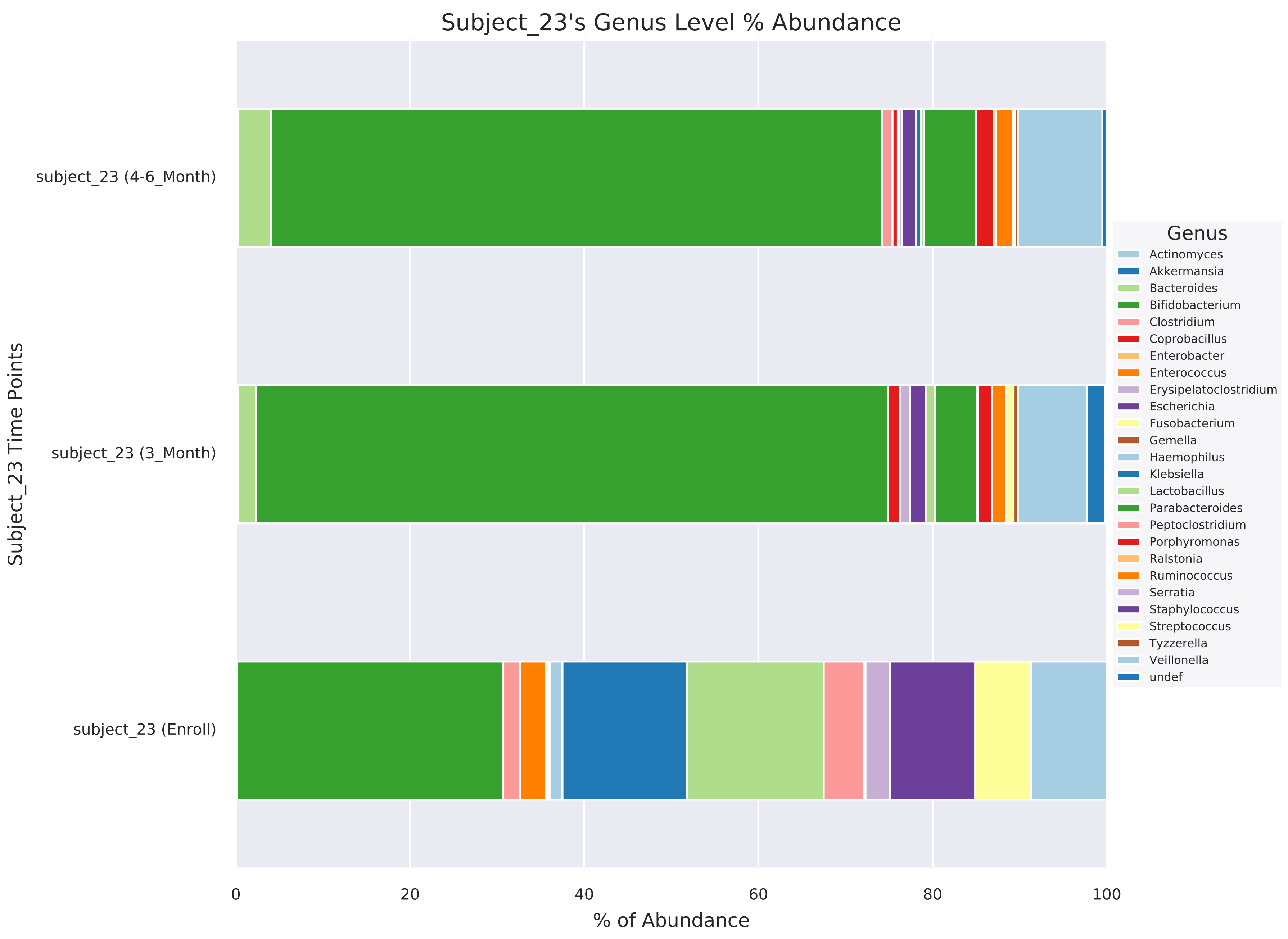
Subject_23's Genus Level % Abundance
subject_23 (4-6_Month)
subject_23 (3_Month)
subject_23 (Enroll)
Subject_23 Time Points
0
20
40
60
80
100
% of Abundance
Genus
Actinomyces
Akkermansia
Bacteroides
Bifidobacterium
Clostridium
Coprobacillus
Enterobacter
Enterococcus
Erysipelatoclostridium
Escherichia
Fusobacterium
Gemella
Haemophilus
Klebsiella
Lactobacillus
Parabacteroides
Peptoclostridium
Porphyromonas
Ralstonia
Ruminococcus
Serratia
Staphylococcus
Streptococcus
Tyzzerella
Veillonella
undef

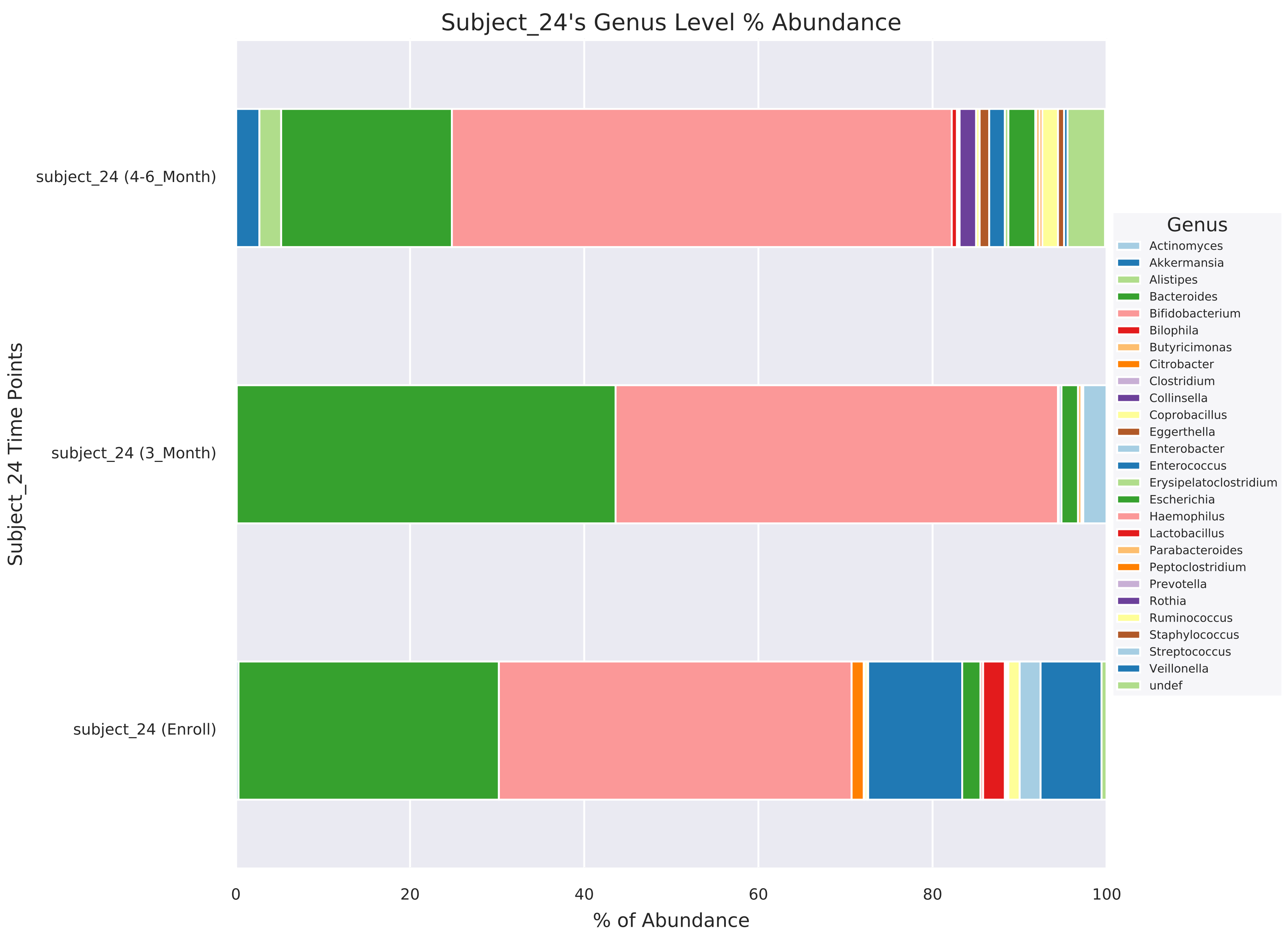
Subject_24's Genus Level % Abundance
subject_24 (4-6_Month)
subject_24 (3_Month)
subject_24 (Enroll)
Subject_24 Time Points
0
20
40
60
80
100
% of Abundance
Genus
Actinomyces
Akkermansia
Alistipes
Bacteroides
Bifidobacterium
Bilophila
Butyricimonas
Citrobacter
Clostridium
Collinsella
Coprobacillus
Eggerthella
Enterobacter
Enterococcus
Erysipelatoclostridium
Escherichia
Haemophilus
Lactobacillus
Parabacteroides
Peptoclostridium
Prevotella
Rothia
Ruminococcus
Staphylococcus
Streptococcus
Veillonella
undef

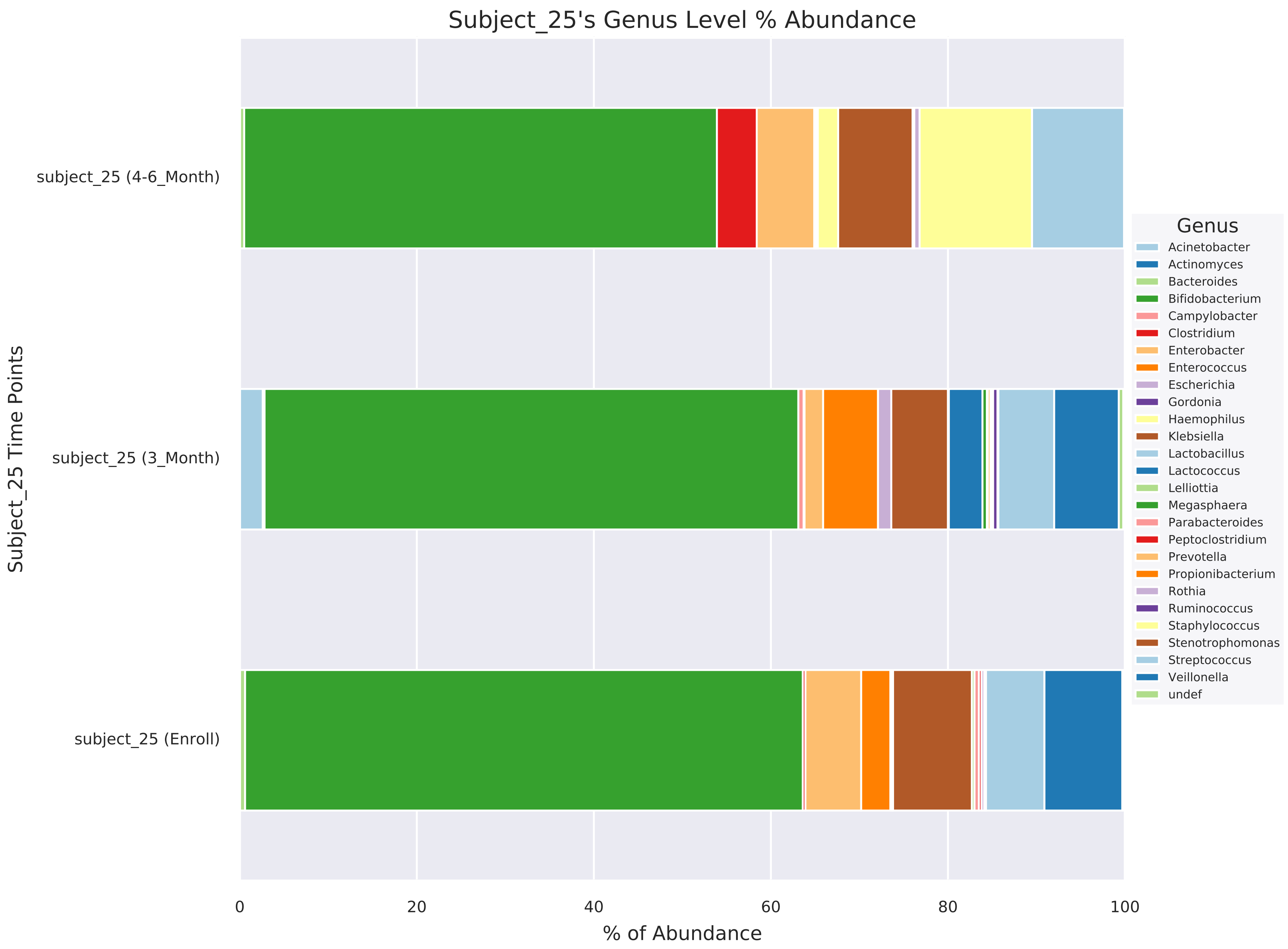
Subject_25's Genus Level % Abundance
Subject_25 Time Points
subject_25 (4-6_Month)
subject_25 (3_Month)
subject_25 (Enroll)
0
20
40
60
80
100
% of Abundance
Genus
Acinetobacter
Actinomyces
Bacteroides
Bifidobacterium
Campylobacter
Clostridium
Enterobacter
Enterococcus
Escherichia
Gordonia
Haemophilus
Klebsiella
Lactobacillus
Lactococcus
Lelliottia
Megasphaera
Parabacteroides
Peptoclostridium
Prevotella
Propionibacterium
Rothia
Ruminococcus
Staphylococcus
Stenotrophomonas
Streptococcus
Veillonella
undef

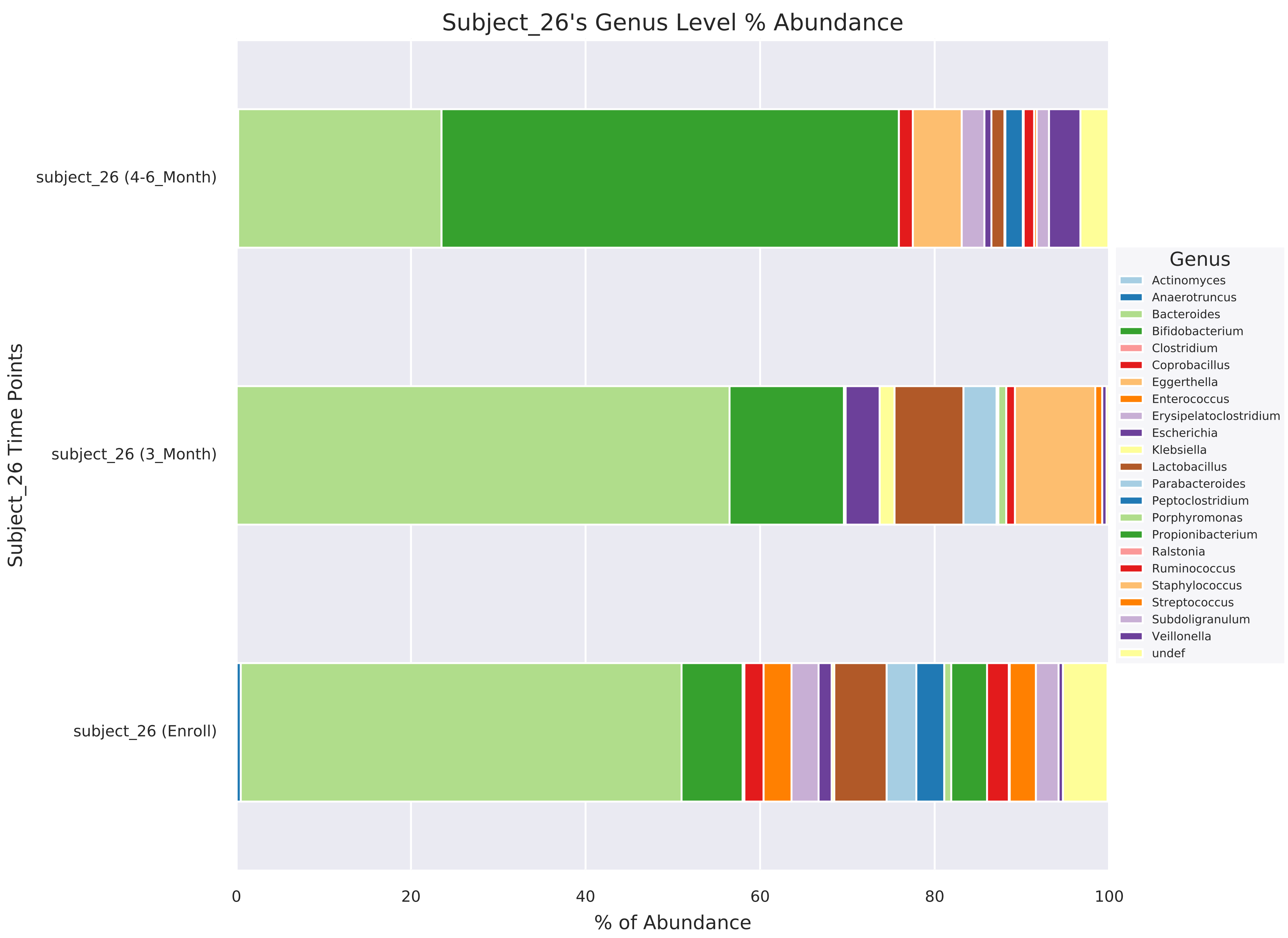

Subject_26's Genus Level % Abundance
subject_26 (4-6_Month)
subject_26 (3_Month)
subject_26 (Enroll)
Subject_26 Time Points
0
20
40
60
80
100
% of Abundance
Genus
Actinomyces
Anaerotruncus
Bacteroides
Bifidobacterium
Clostridium
Coprobacillus
Eggerthella
Enterococcus
Erysipelatoclostridium
Escherichia
Klebsiella
Lactobacillus
Parabacteroides
Peptoclostridium
Porphyromonas
Propionibacterium
Ralstonia
Ruminococcus
Staphylococcus
Streptococcus
Subdoligranulum
Veillonella
undef

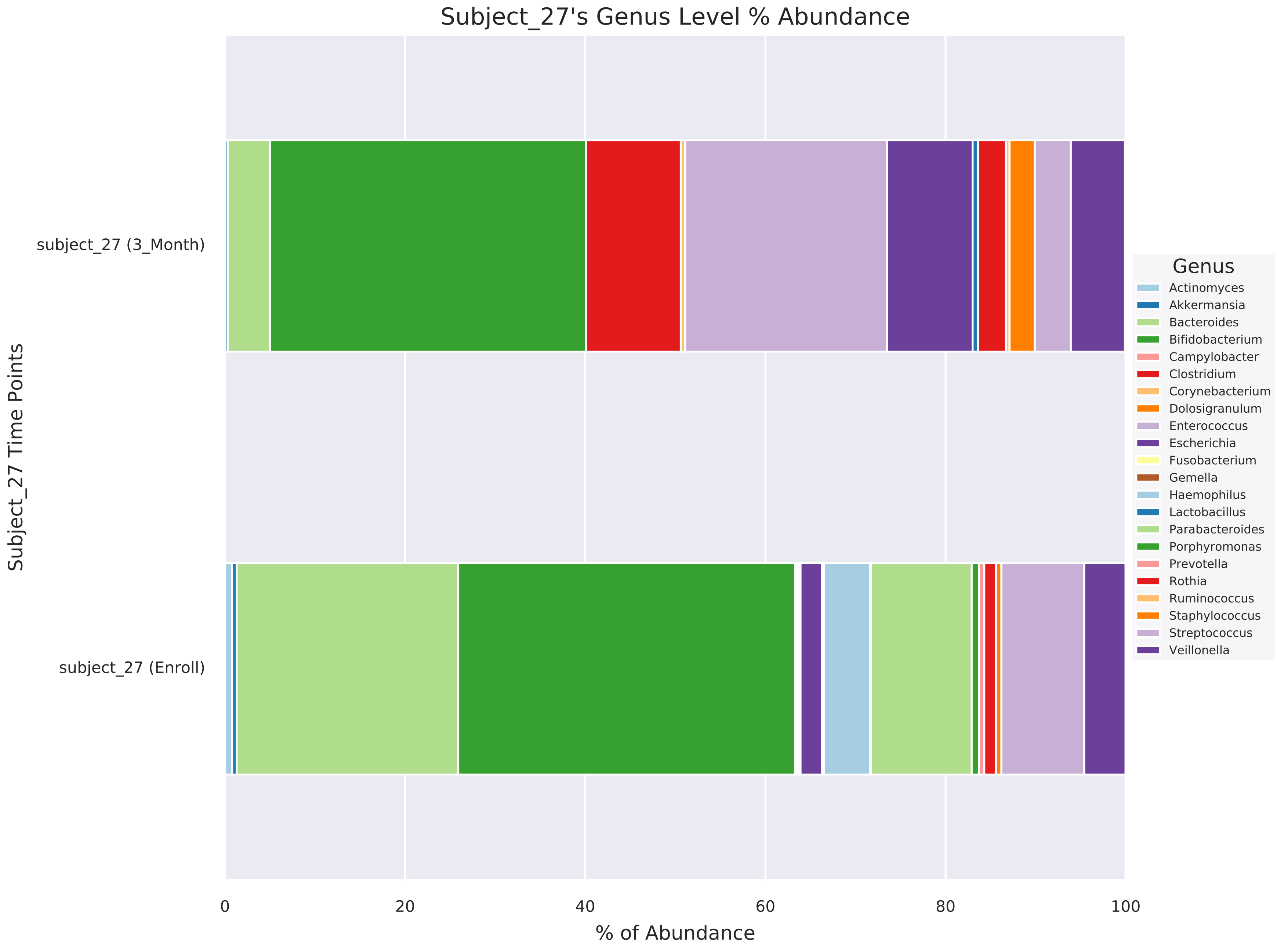
Subject_27's Genus Level % Abundance
subject_27 (3_Month)
subject_27 (Enroll)
Subject_27 Time Points
0
20
40
60
80
100
% of Abundance
Genus
Actinomyces
Akkermansia
Bacteroides
Bifidobacterium
Campylobacter
Clostridium
Corynebacterium
Dolosigranulum
Enterococcus
Escherichia
Fusobacterium
Gemella
Haemophilus
Lactobacillus
Parabacteroides
Porphyromonas
Prevotella
Rothia
Ruminococcus
Staphylococcus
Streptococcus
Veillonella

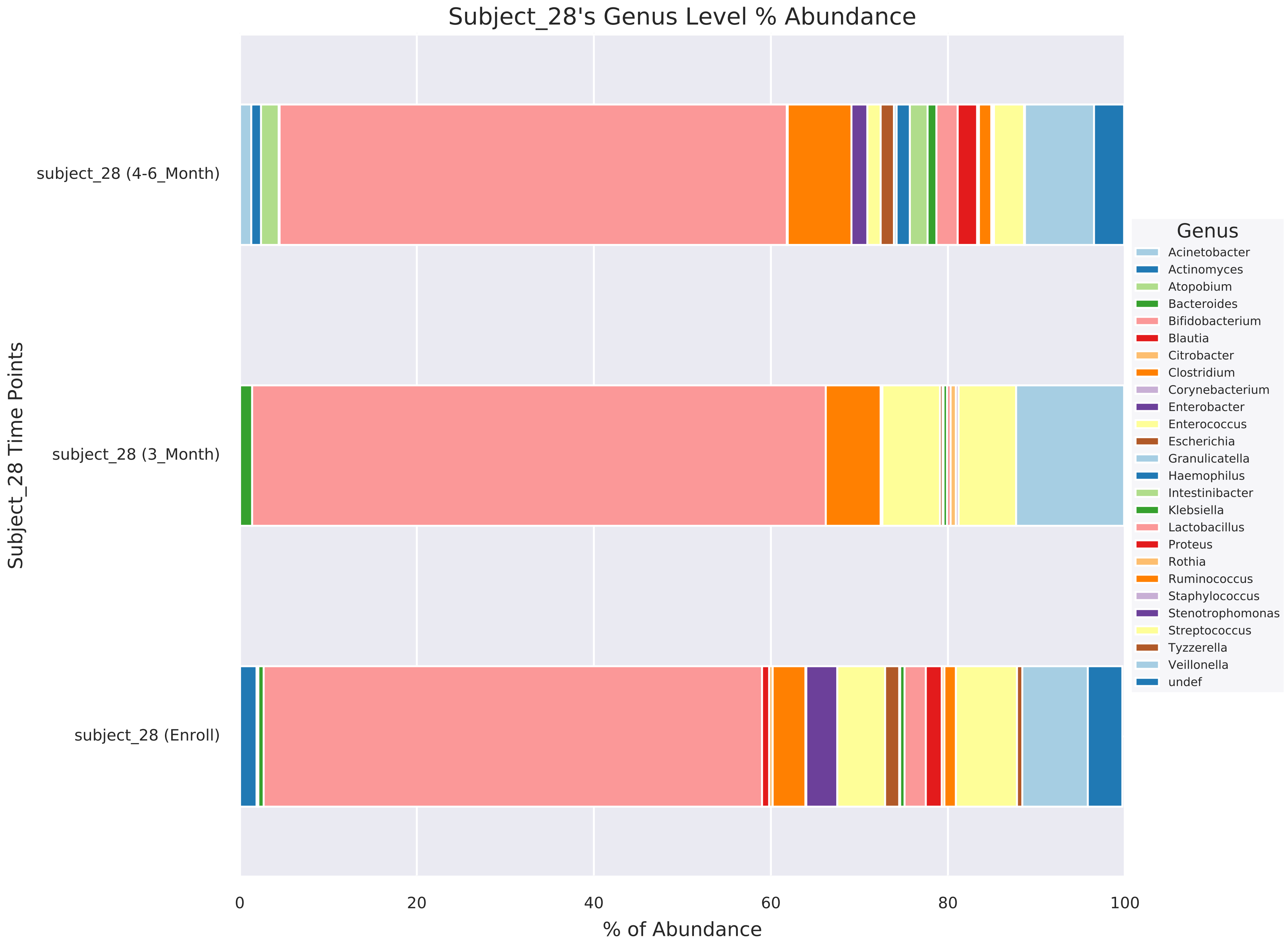

Subject_28's Genus Level % Abundance
subject_28 (4-6_Month)
subject_28 (3_Month)
subject_28 (Enroll)
Subject_28 Time Points
0
20
40
60
80
100
% of Abundance
Genus
Acinetobacter
Actinomyces
Atopobium
Bacteroides
Bifidobacterium
Blautia
Citrobacter
Clostridium
Corynebacterium
Enterobacter
Enterococcus
Escherichia
Granulicatella
Haemophilus
Intestinibacter
Klebsiella
Lactobacillus
Proteus
Rothia
Ruminococcus
Staphylococcus
Stenotrophomonas
Streptococcus
Tyzzerella
Veillonella
undef

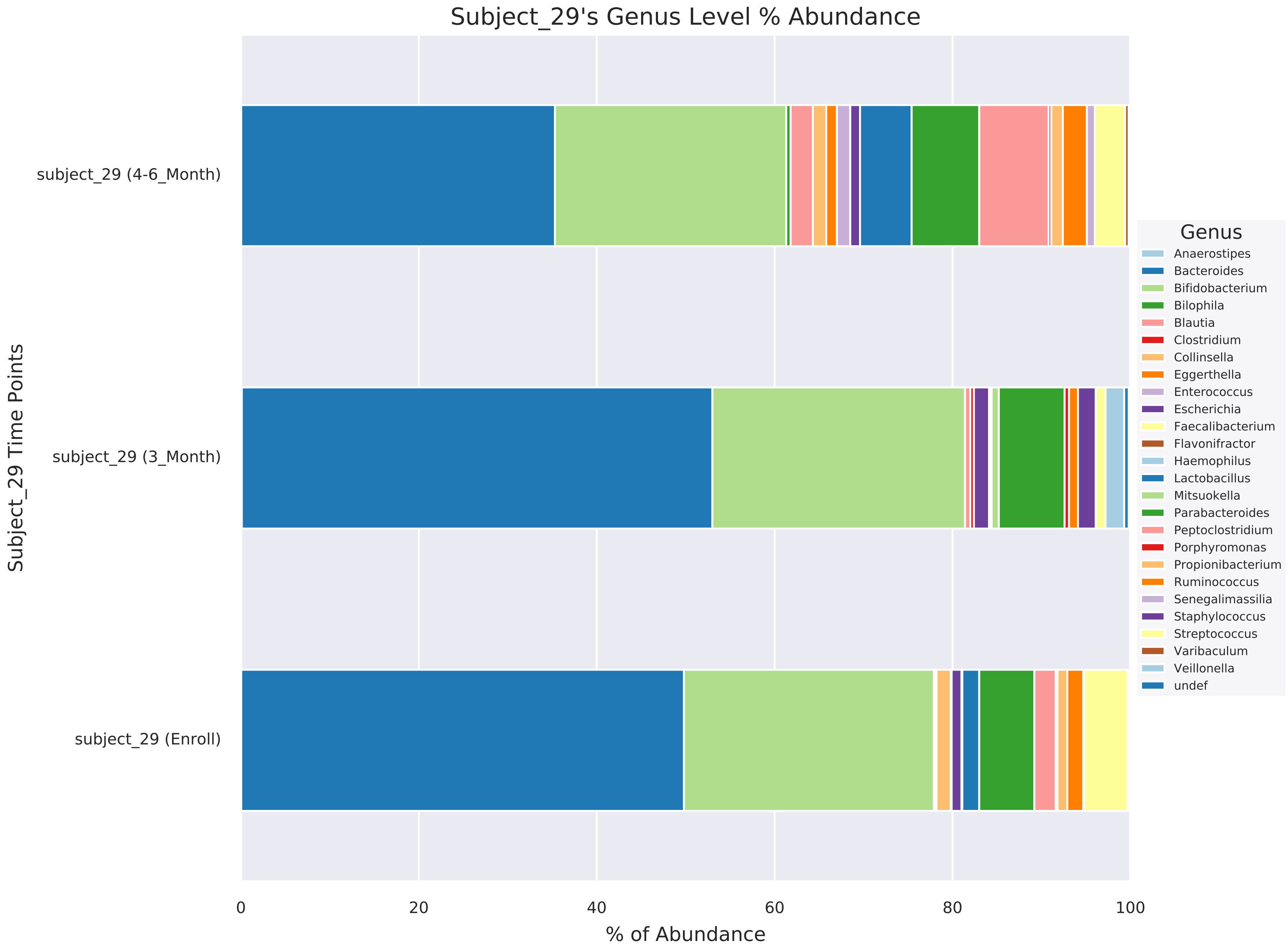
Subject_29's Genus Level % Abundance
subject_29 (4-6_Month)
subject_29 (3_Month)
subject_29 (Enroll)
Subject_29 Time Points
0
20
40
60
80
100
% of Abundance
Genus
Anaerostipes
Bacteroides
Bifidobacterium
Bilophila
Blautia
Clostridium
Collinsella
Eggerthella
Enterococcus
Escherichia
Faecalibacterium
Flavonifractor
Haemophilus
Lactobacillus
Mitsuokella
Parabacteroides
Peptoclostridium
Porphyromonas
Propionibacterium
Ruminococcus
Senegalimassilia
Staphylococcus
Streptococcus
Varibaculum
Veillonella
undef

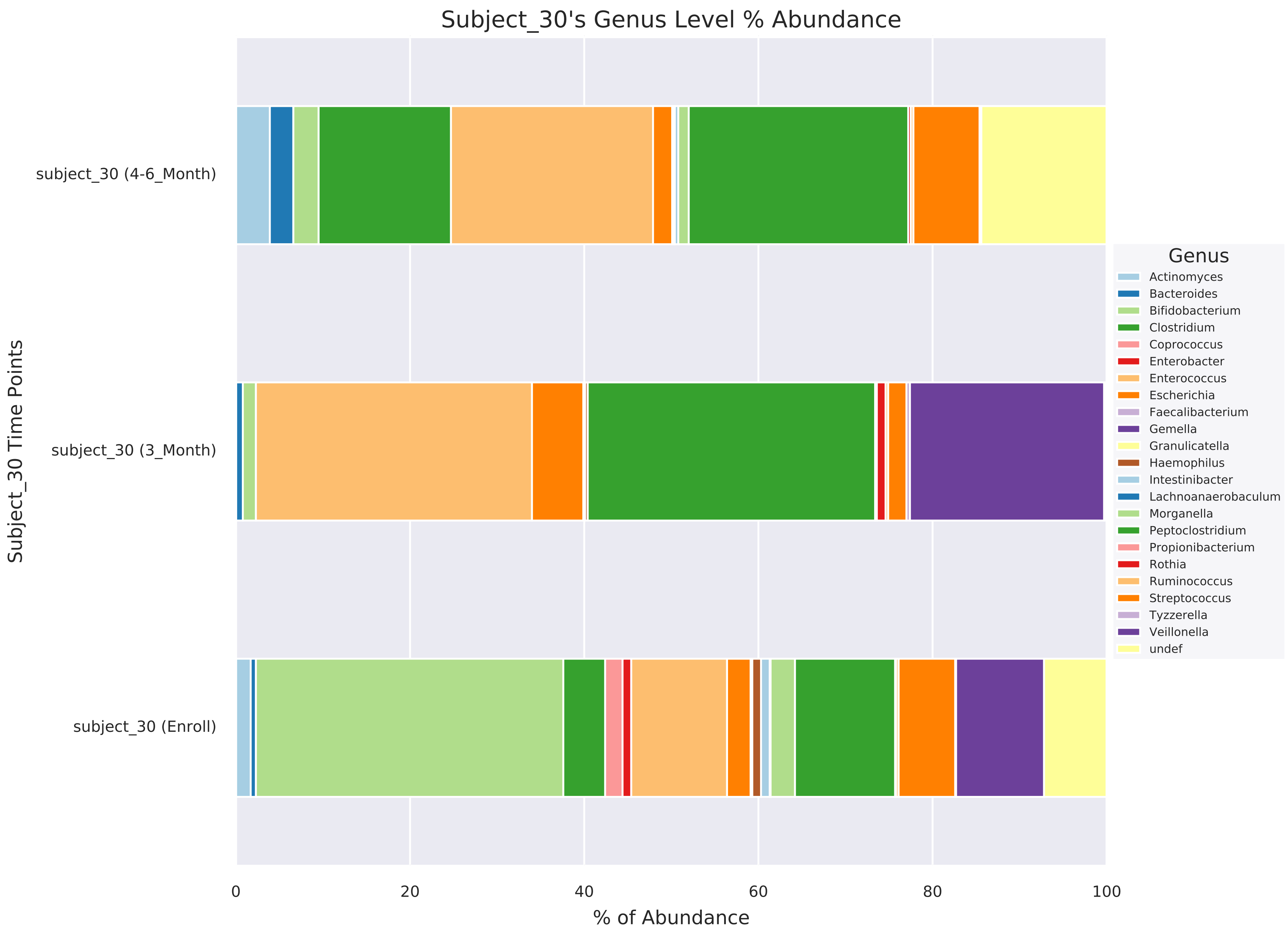

Subject_30's Genus Level % Abundance
subject_30 (4-6_Month)
subject_30 (3_Month)
subject_30 (Enroll)
Subject_30 Time Points
0
20
40
60
80
100
% of Abundance
Genus
Actinomyces
Bacteroides
Bifidobacterium
Clostridium
Coprococcus
Enterobacter
Enterococcus
Escherichia
Faecalibacterium
Gemella
Granulicatella
Haemophilus
Intestinibacter
Lachnoanaerobaculum
Morganella
Peptoclostridium
Propionibacterium
Rothia
Ruminococcus
Streptococcus
Tyzzerella
Veillonella
undef

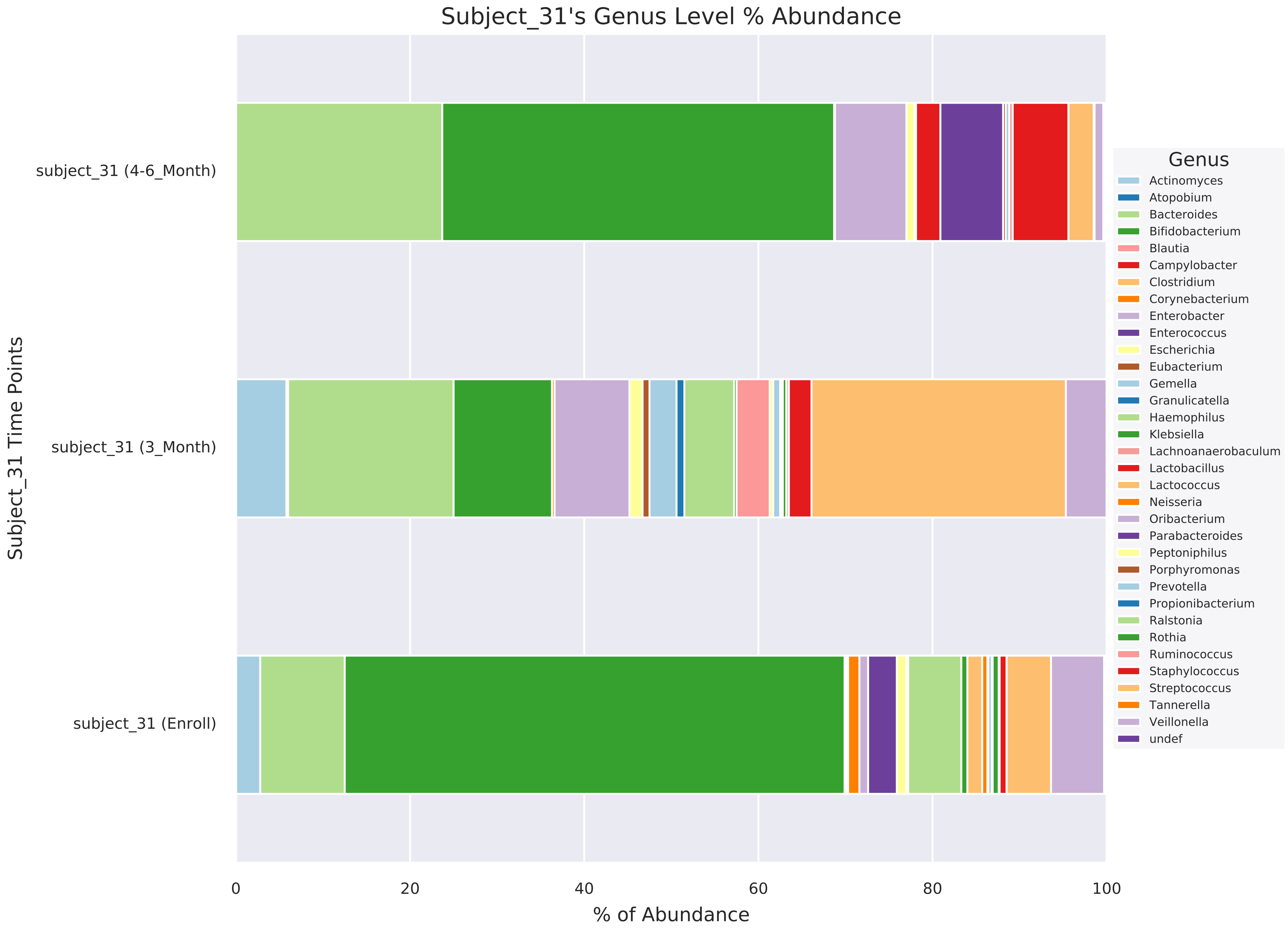

Subject_31's Genus Level % Abundance
Subject_31 Time Points
subject_31 (4-6_Month)
subject_31 (3_Month)
subject_31 (Enroll)
0
20
40
60
80
100
% of Abundance
Genus
Actinomyces
Atopobium
Bacteroides
Bifidobacterium
Blautia
Campylobacter
Clostridium
Corynebacterium
Enterobacter
Enterococcus
Escherichia
Eubacterium
Gemella
Granulicatella
Haemophilus
Klebsiella
Lachnoanaerobaculum
Lactobacillus
Lactococcus
Neisseria
Oribacterium
Parabacteroides
Peptoniphilus
Porphyromonas
Prevotella
Propionibacterium
Ralstonia
Rothia
Ruminococcus
Staphylococcus
Streptococcus
Tannerella
Veillonella
undef

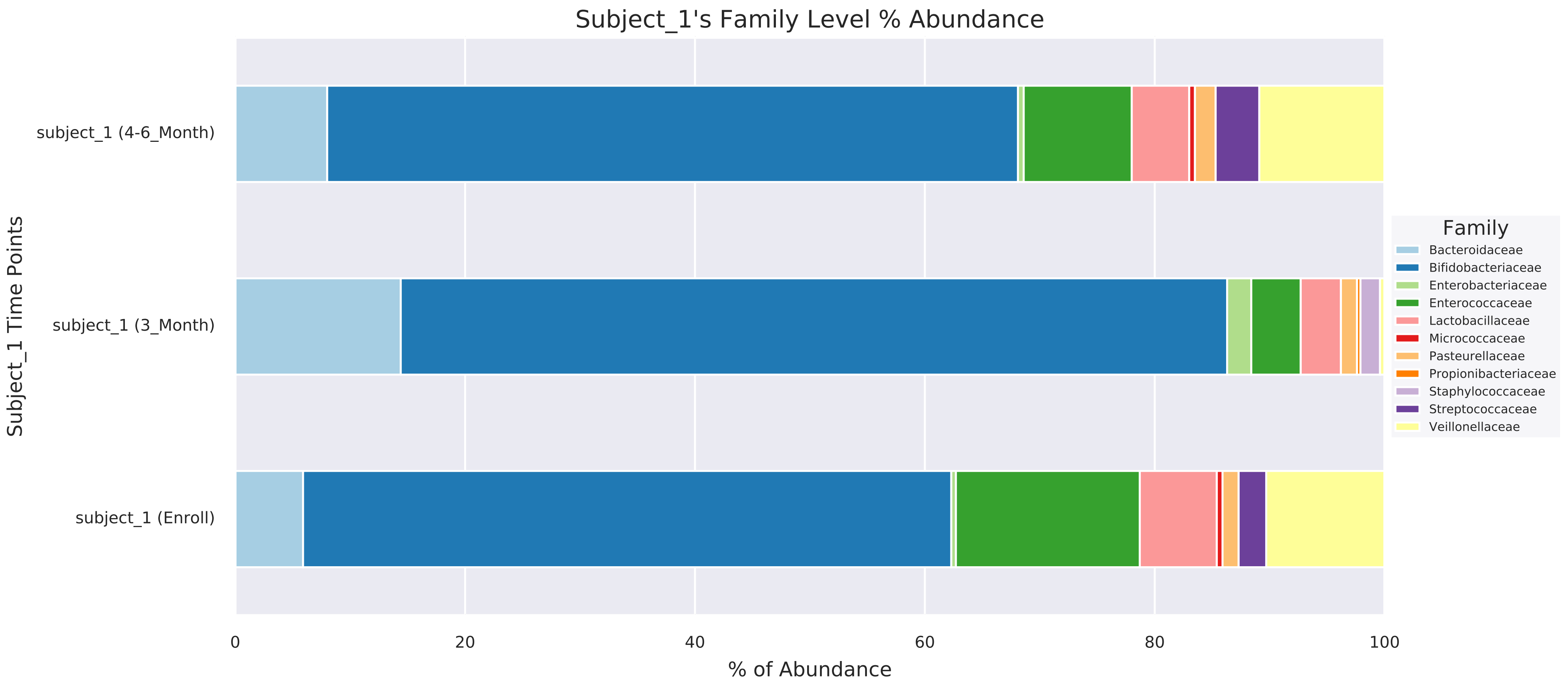
Subject_1's Family Level % Abundance
Subject_1 Time Points
subject_1 (4-6_Month)
subject_1 (3_Month)
subject_1 (Enroll)
0
20
40
60
80
100
% of Abundance
Family
Bacteroidaceae
Bifidobacteriaceae
Enterobacteriaceae
Enterococcaceae
Lactobacillaceae
Micrococcaceae
Pasteurellaceae
Propionibacteriaceae
Staphylococcaceae
Streptococcaceae
Veillonellaceae

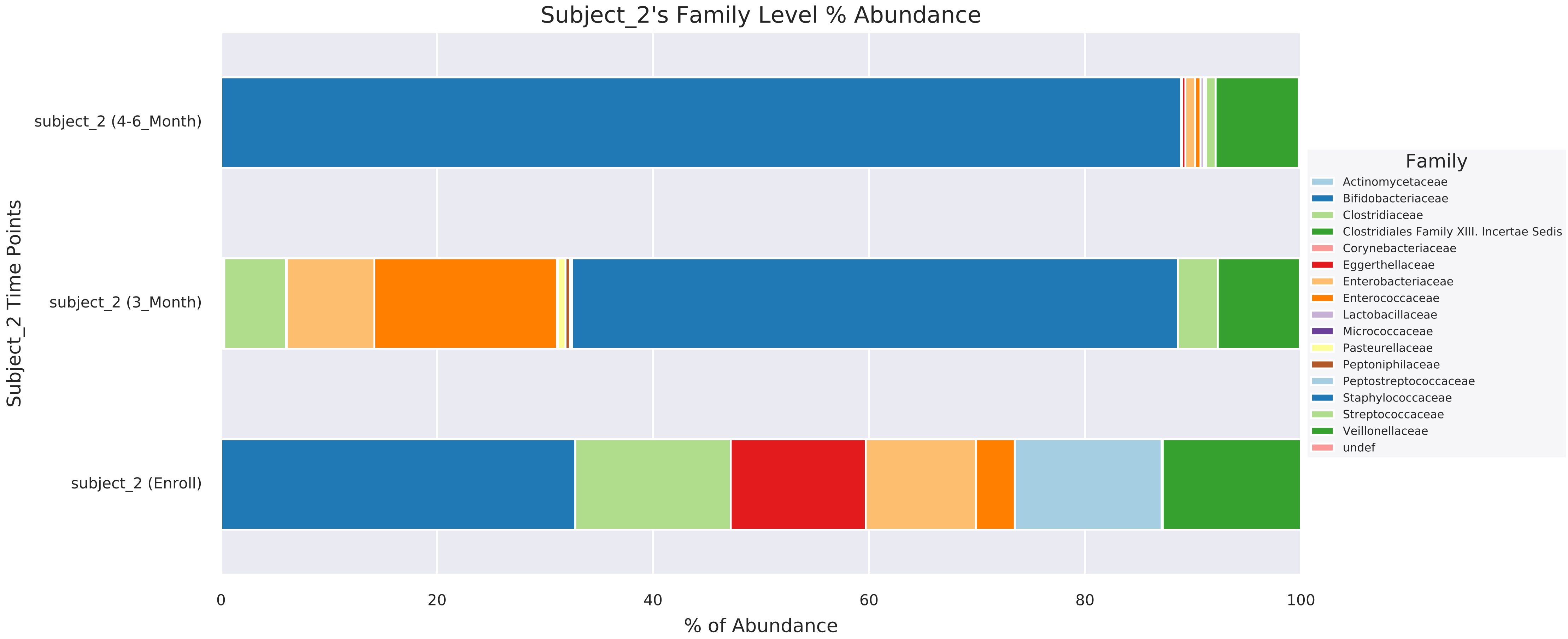

Subject_2's Family Level % Abundance
Subject_2 Time Points
subject_2 (4-6_Month)
subject_2 (3_Month)
subject_2 (Enroll)
0
20
40
60
80
100
% of Abundance
Family
Actinomycetaceae
Bifidobacteriaceae
Clostridiaceae
Clostridiales Family XIII. Incertae Sedis
Corynebacteriaceae
Eggerthellaceae
Enterobacteriaceae
Enterococcaceae
Lactobacillaceae
Micrococcaceae
Pasteurellaceae
Peptoniphilaceae
Peptostreptococcaceae
Staphylococcaceae
Streptococcaceae
Veillonellaceae
undef

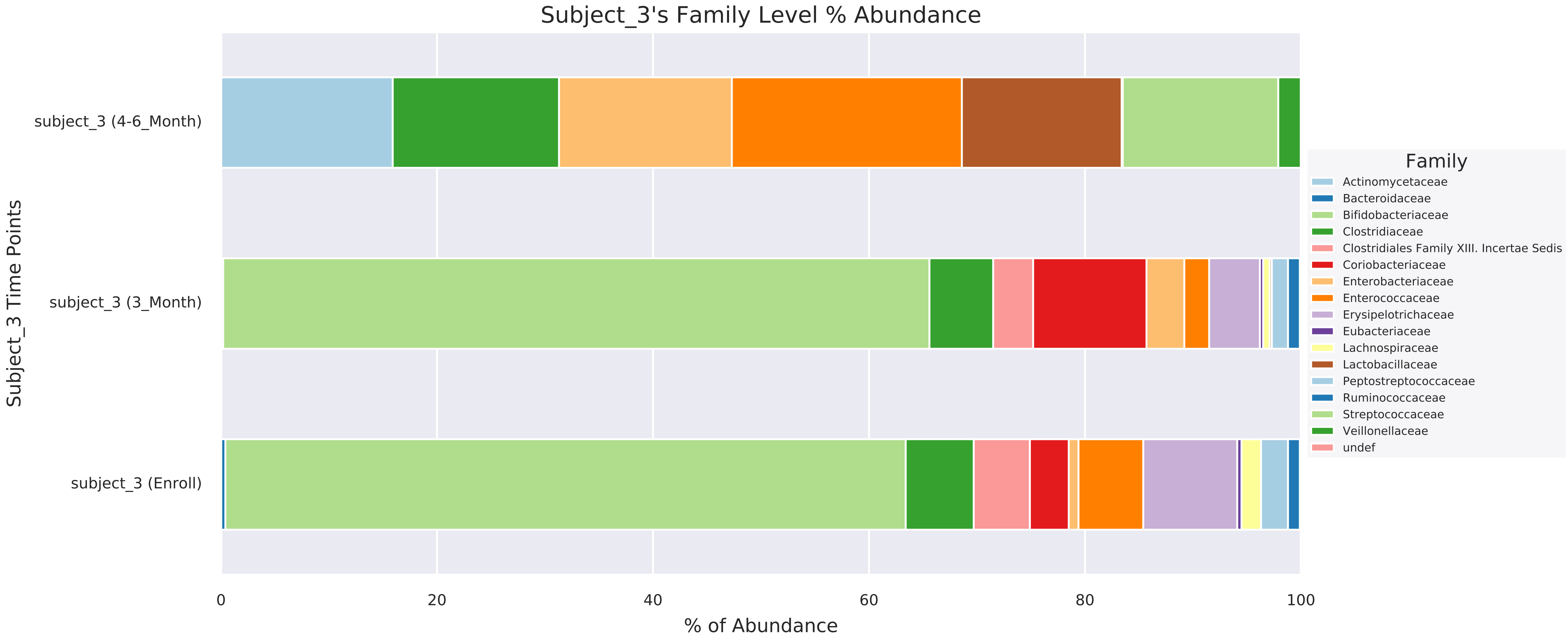
Subject_3's Family Level % Abundance
Subject_3 Time Points
subject_3 (4-6_Month)
subject_3 (3_Month)
subject_3 (Enroll)
0
20
40
60
80
100
% of Abundance
Family
Actinomycetaceae
Bacteroidaceae
Bifidobacteriaceae
Clostridiaceae
Clostridiales Family XIII. Incertae Sedis
Coriobacteriaceae
Enterobacteriaceae
Enterococcaceae
Erysipelotrichaceae
Eubacteriaceae
Lachnospiraceae
Lactobacillaceae
Peptostreptococcaceae
Ruminococcaceae
Streptococcaceae
Veillonellaceae
undef

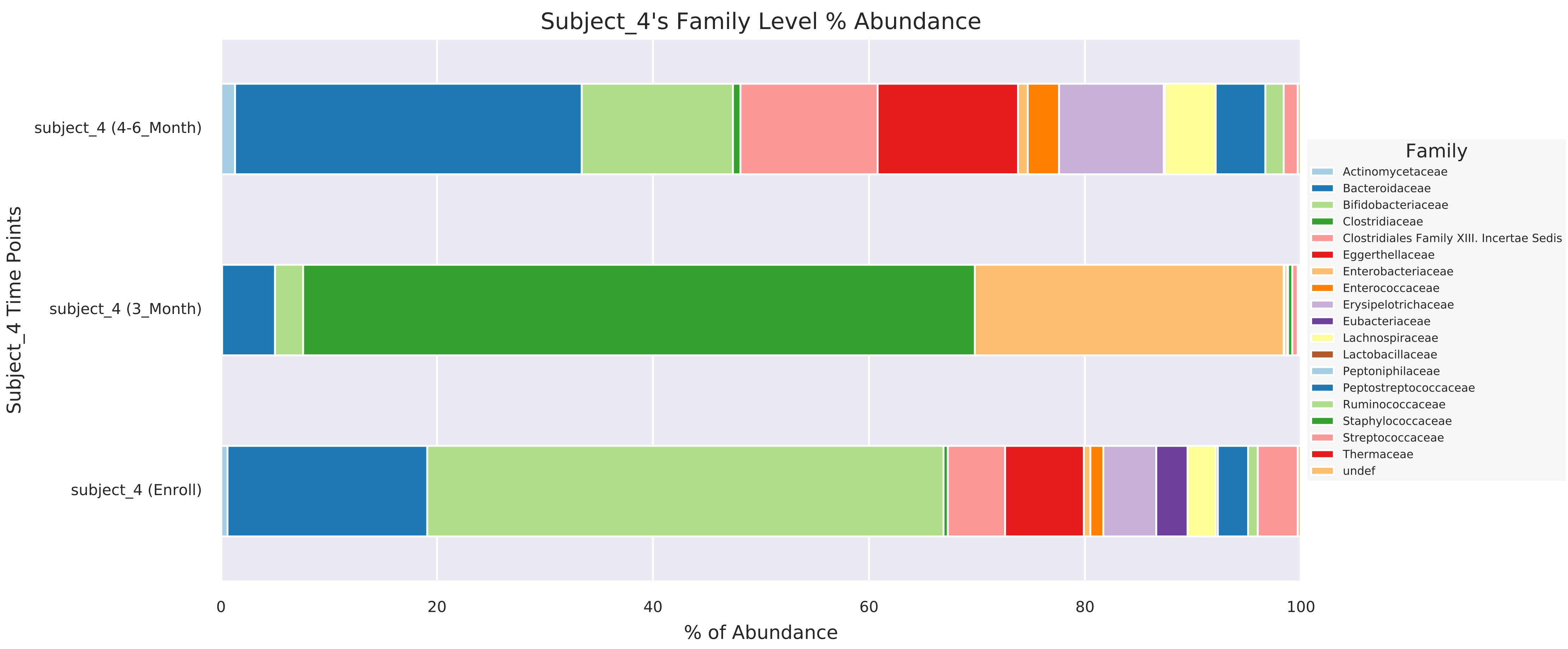
Subject_4's Family Level % Abundance
subject_4 (4-6_Month)
subject_4 (3_Month)
subject_4 (Enroll)
Subject_4 Time Points
0
20
40
60
80
100
% of Abundance
Family
Actinomycetaceae
Bacteroidaceae
Bifidobacteriaceae
Clostridiaceae
Clostridiales Family XIII. Incertae Sedis
Eggerthellaceae
Enterobacteriaceae
Enterococcaceae
Erysipelotrichaceae
Eubacteriaceae
Lachnospiraceae
Lactobacillaceae
Peptoniphilaceae
Peptostreptococcaceae
Ruminococcaceae
Staphylococcaceae
Streptococcaceae
Thermaceae
undef

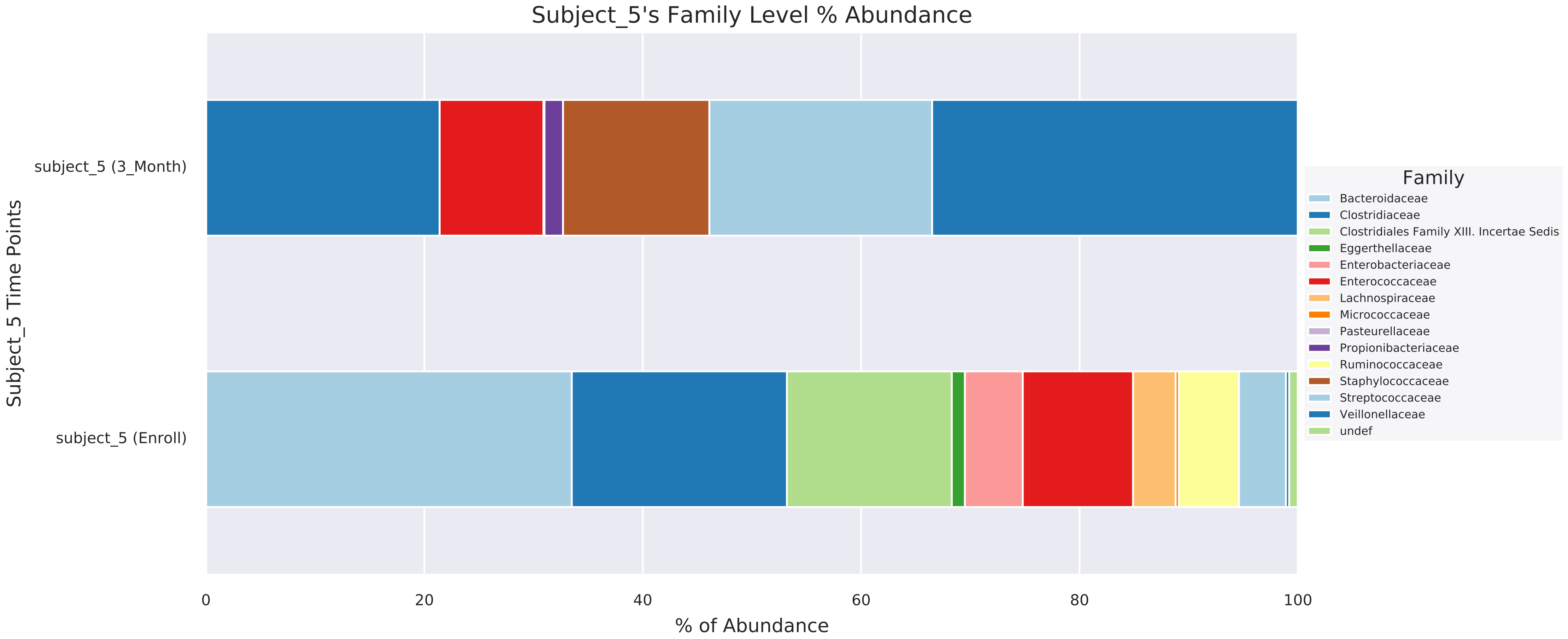
Subject_5's Family Level % Abundance
subject_5 (3_Month)
subject_5 (Enroll)
Subject_5 Time Points
0
20
40
60
80
100
% of Abundance
Family
Bacteroidaceae
Clostridiaceae
Clostridiales Family XIII. Incertae Sedis
Eggerthellaceae
Enterobacteriaceae
Enterococcaceae
Lachnospiraceae
Micrococcaceae
Pasteurellaceae
Propionibacteriaceae
Ruminococcaceae
Staphylococcaceae
Streptococcaceae
Veillonellaceae
undef

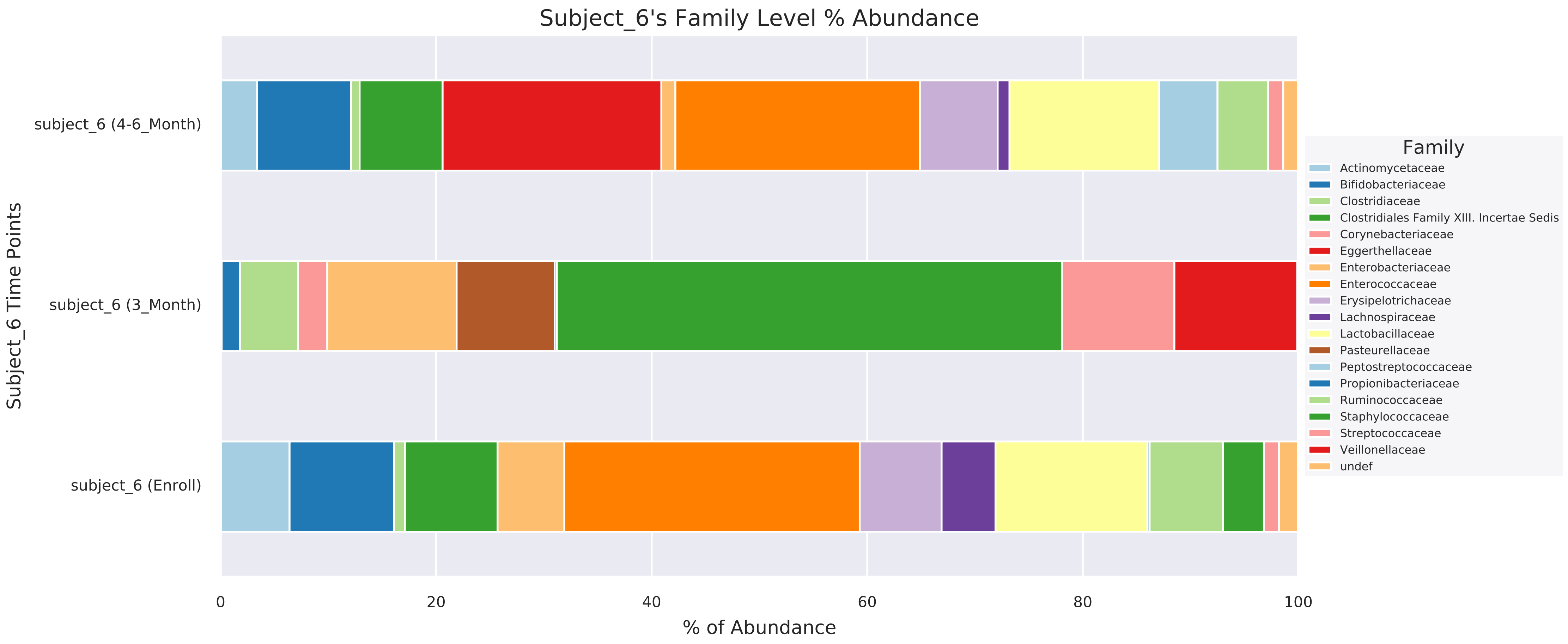
Subject_6's Family Level % Abundance
subject_6 (4-6_Month)
subject_6 (3_Month)
subject_6 (Enroll)
Subject_6 Time Points
0
20
40
60
80
100
% of Abundance
Family
Actinomycetaceae
Bifidobacteriaceae
Clostridiaceae
Clostridiales Family XIII. Incertae Sedis
Corynebacteriaceae
Eggerthellaceae
Enterobacteriaceae
Enterococcaceae
Erysipelotrichaceae
Lachnospiraceae
Lactobacillaceae
Pasteurellaceae
Peptostreptococcaceae
Propionibacteriaceae
Ruminococcaceae
Staphylococcaceae
Streptococcaceae
Veillonellaceae
undef

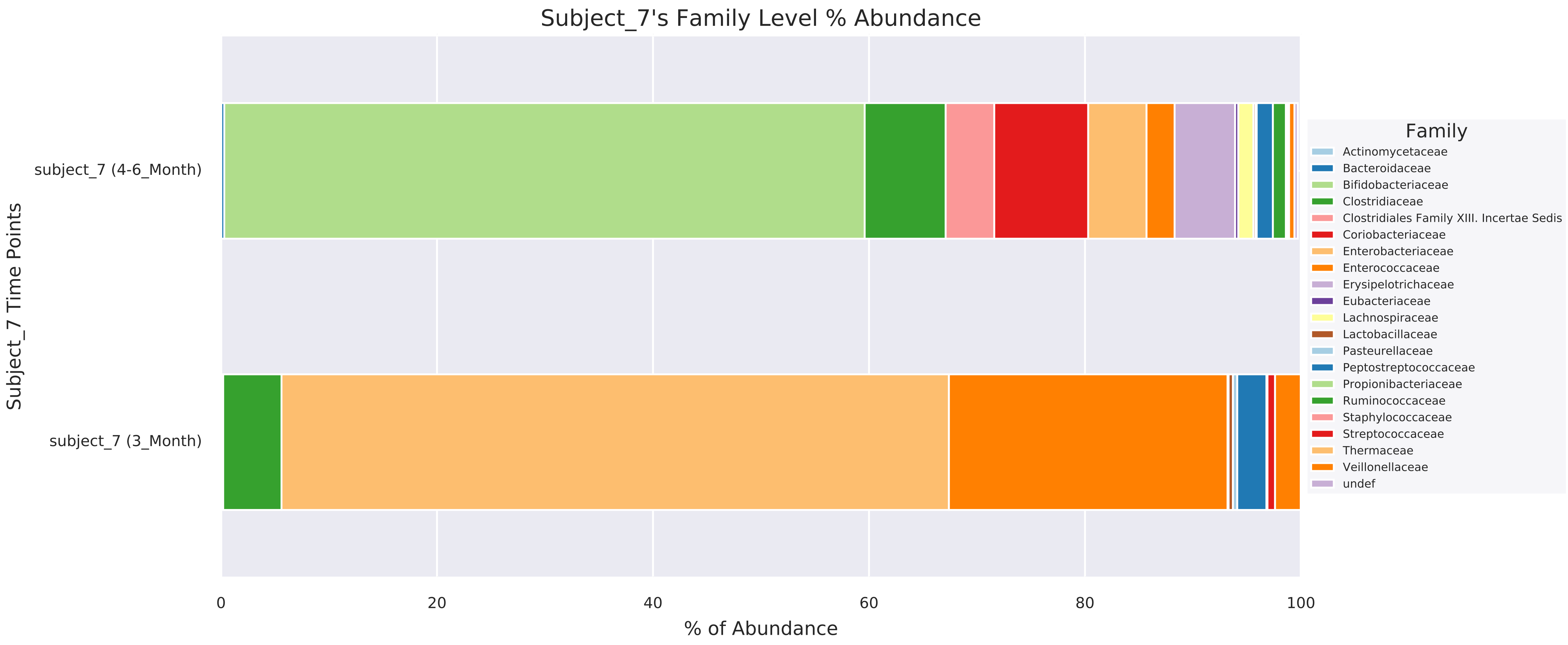
Subject_7's Family Level % Abundance
subject_7 (4-6_Month)
subject_7 (3_Month)
Subject_7 Time Points
0
20
40
60
80
100
% of Abundance
Family
Actinomycetaceae
Bacteroidaceae
Bifidobacteriaceae
Clostridiaceae
Clostridiales Family XIII. Incertae Sedis
Coriobacteriaceae
Enterobacteriaceae
Enterococcaceae
Erysipelotrichaceae
Eubacteriaceae
Lachnospiraceae
Lactobacillaceae
Pasteurellaceae
Peptostreptococcaceae
Propionibacteriaceae
Ruminococcaceae
Staphylococcaceae
Streptococcaceae
Thermaceae
Veillonellaceae
undef

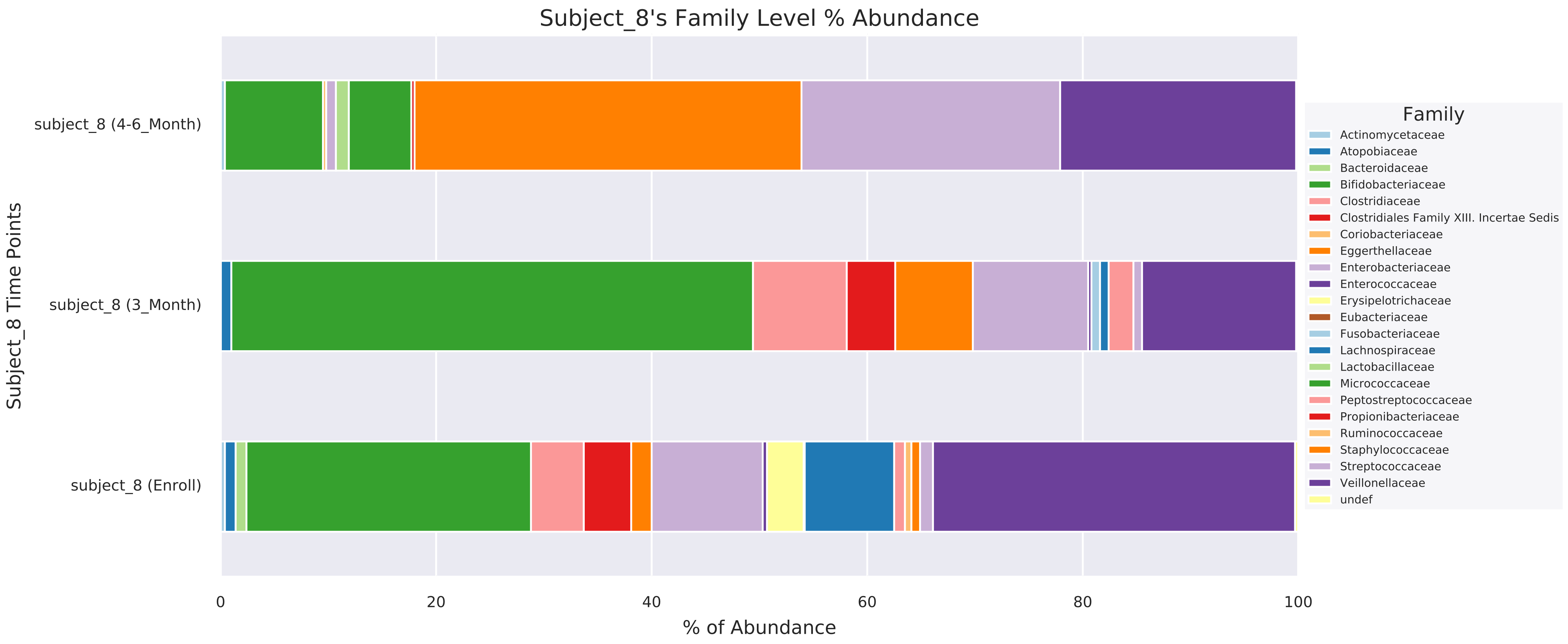

Subject_8's Family Level % Abundance
Subject_8 Time Points
subject_8 (4-6_Month)
subject_8 (3_Month)
subject_8 (Enroll)
0
20
40
60
80
100
% of Abundance
Family
Actinomycetaceae
Atopobiaceae
Bacteroidaceae
Bifidobacteriaceae
Clostridiaceae
Clostridiales Family XIII. Incertae Sedis
Coriobacteriaceae
Eggerthellaceae
Enterobacteriaceae
Enterococcaceae
Erysipelotrichaceae
Eubacteriaceae
Fusobacteriaceae
Lachnospiraceae
Lactobacillaceae
Micrococcaceae
Peptostreptococcaceae
Propionibacteriaceae
Ruminococcaceae
Staphylococcaceae
Streptococcaceae
Veillonellaceae
undef

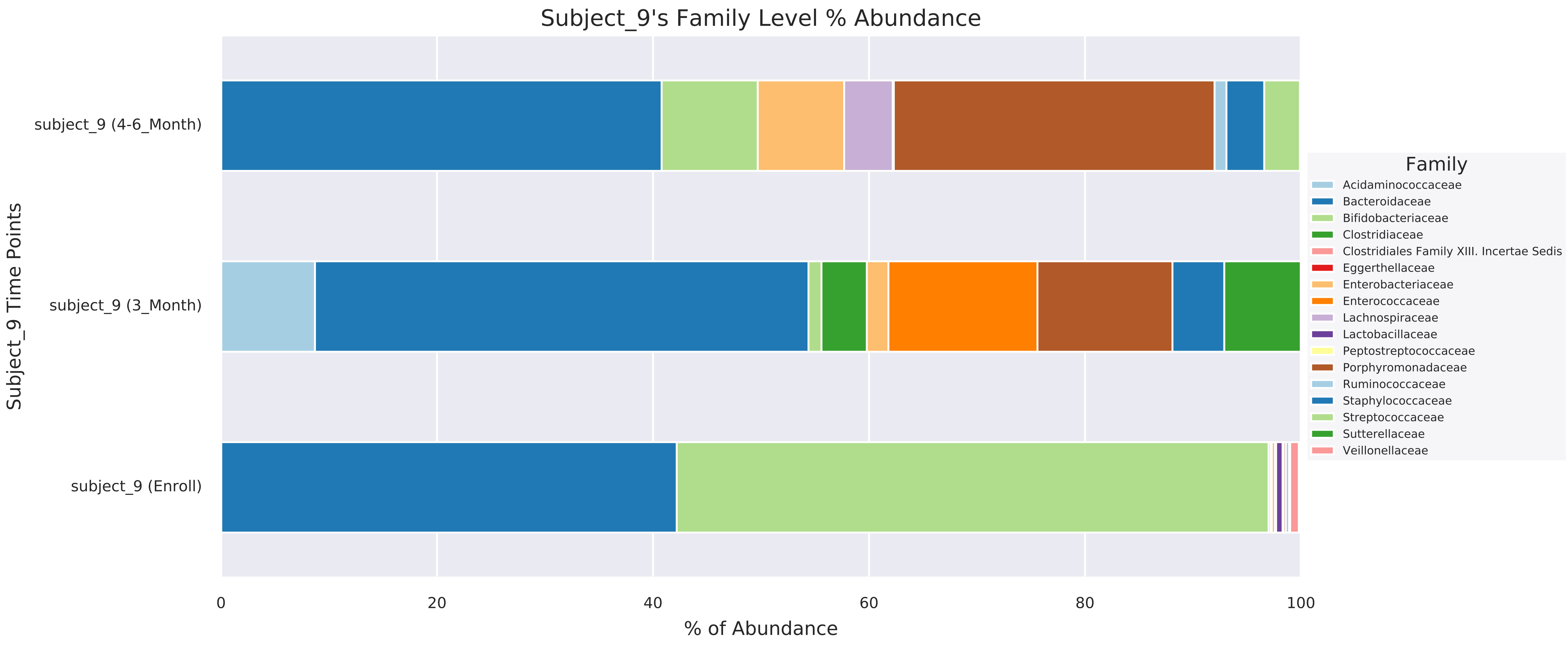

Subject_9's Family Level % Abundance
Subject_9 Time Points
subject_9 (4-6_Month)
subject_9 (3_Month)
subject_9 (Enroll)
0
20
40
60
80
100
% of Abundance
Family
Acidaminococcaceae
Bacteroidaceae
Bifidobacteriaceae
Clostridiaceae
Clostridiales Family XIII. Incertae Sedis
Eggerthellaceae
Enterobacteriaceae
Enterococcaceae
Lachnospiraceae
Lactobacillaceae
Peptostreptococcaceae
Porphyromonadaceae
Ruminococcaceae
Staphylococcaceae
Streptococcaceae
Sutterellaceae
Veillonellaceae

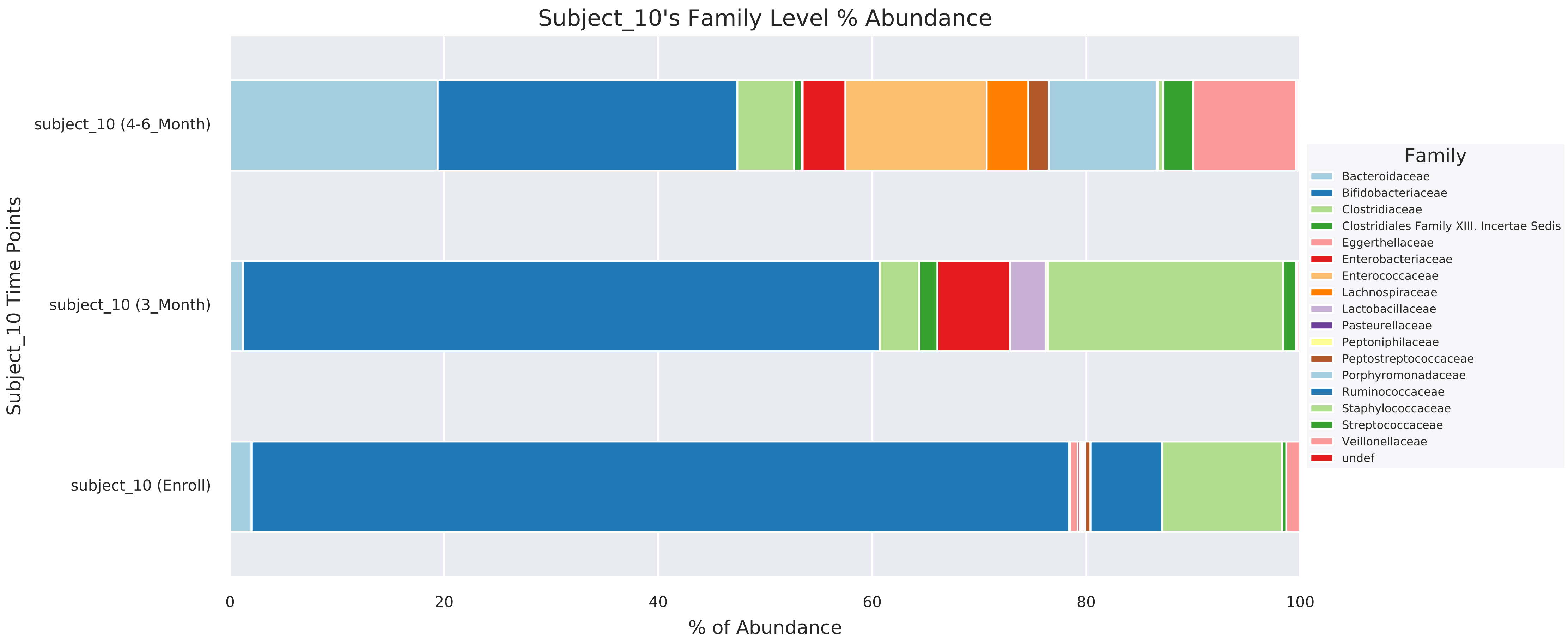
Subject_10's Family Level % Abundance
subject_10 (4-6_Month)
subject_10 (3_Month)
subject_10 (Enroll)
Subject_10 Time Points
0
20
40
60
80
100
% of Abundance
Family
Bacteroidaceae
Bifidobacteriaceae
Clostridiaceae
Clostridiales Family XIII. Incertae Sedis
Eggerthellaceae
Enterobacteriaceae
Enterococcaceae
Lachnospiraceae
Lactobacillaceae
Pasteurellaceae
Peptoniphilaceae
Peptostreptococcaceae
Porphyromonadaceae
Ruminococcaceae
Staphylococcaceae
Streptococcaceae
Veillonellaceae
undef

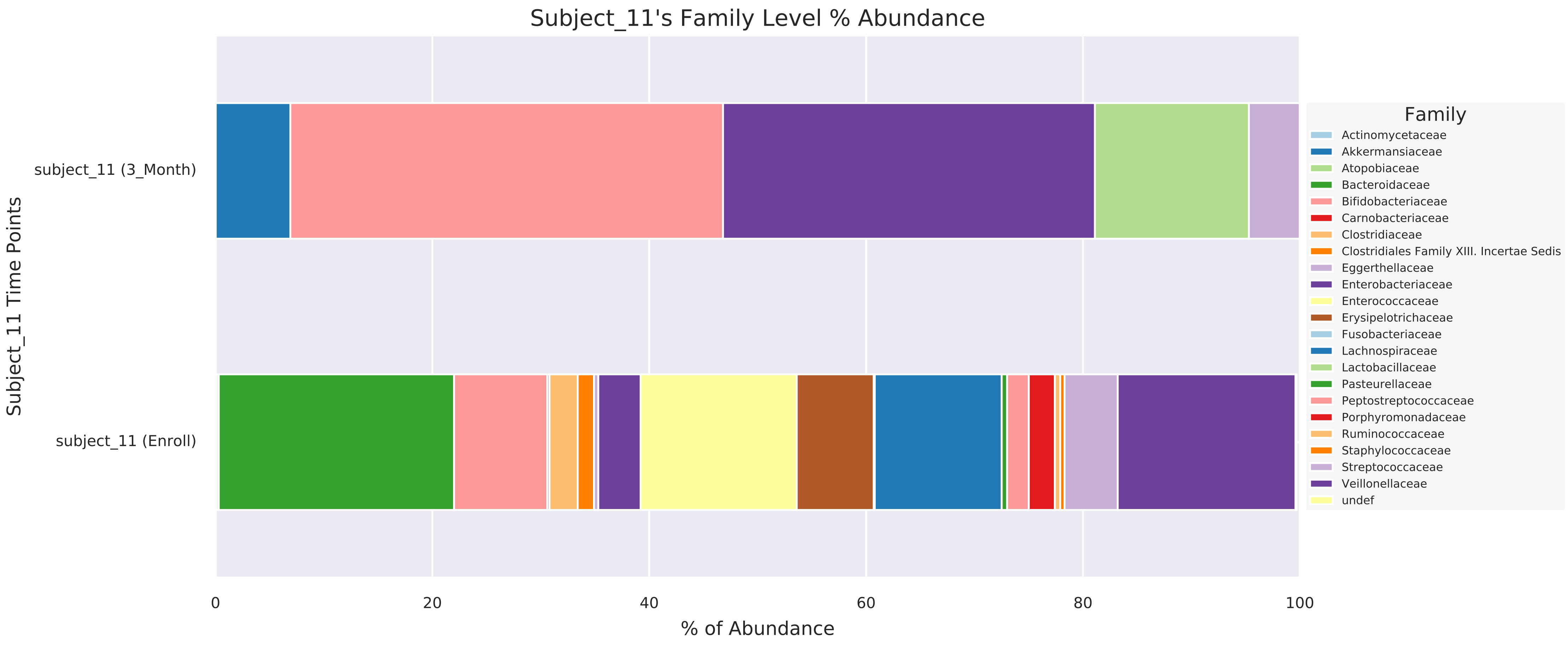

Subject_11's Family Level % Abundance
Subject_11 Time Points
subject_11 (3_Month)
subject_11 (Enroll)
0
20
40
60
80
100
% of Abundance
Family
Actinomycetaceae
Akkermansiaceae
Atopobiaceae
Bacteroidaceae
Bifidobacteriaceae
Carnobacteriaceae
Clostridiaceae
Clostridiales Family XIII. Incertae Sedis
Eggerthellaceae
Enterobacteriaceae
Enterococcaceae
Erysipelotrichaceae
Fusobacteriaceae
Lachnospiraceae
Lactobacillaceae
Pasteurellaceae
Peptostreptococcaceae
Porphyromonadaceae
Ruminococcaceae
Staphylococcaceae
Streptococcaceae
Veillonellaceae
undef

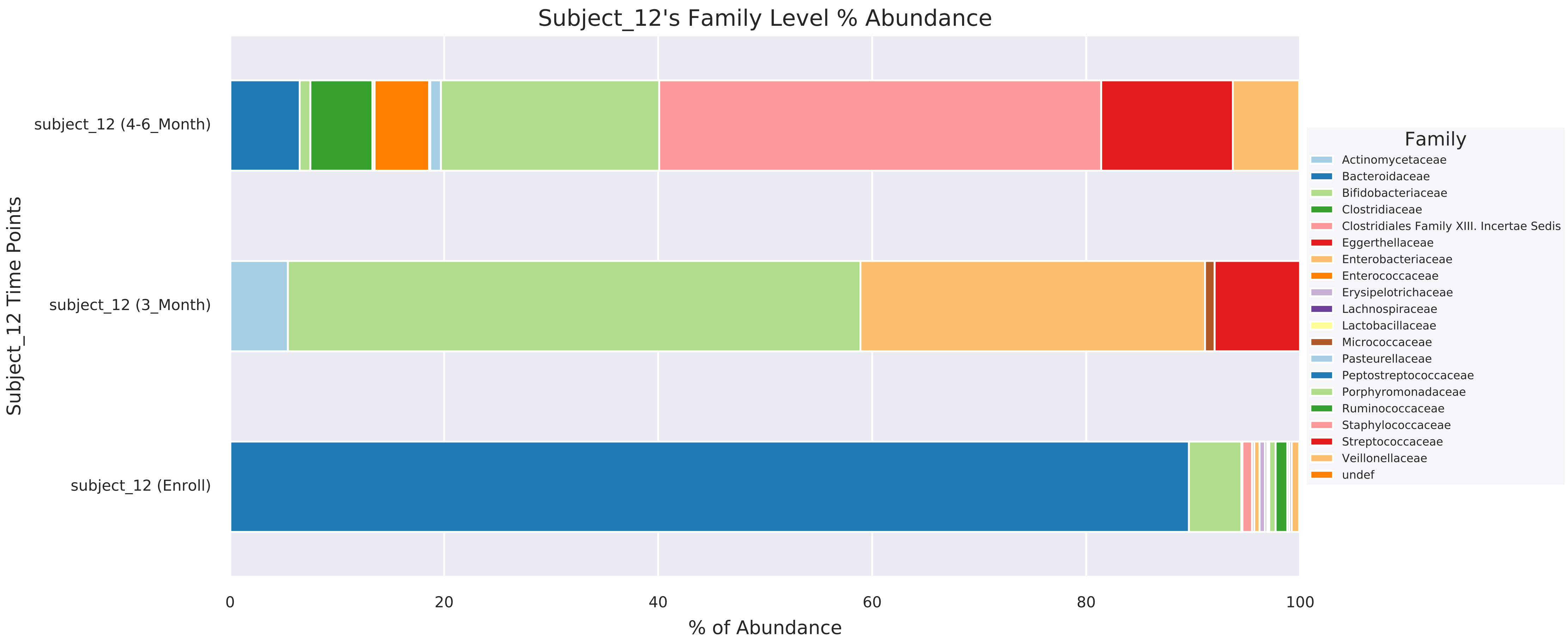
Subject_12's Family Level % Abundance
Subject_12 Time Points
subject_12 (4-6_Month)
subject_12 (3_Month)
subject_12 (Enroll)
0
20
40
60
80
100
% of Abundance
Family
Actinomycetaceae
Bacteroidaceae
Bifidobacteriaceae
Clostridiaceae
Clostridiales Family XIII. Incertae Sedis
Eggerthellaceae
Enterobacteriaceae
Enterococcaceae
Erysipelotrichaceae
Lachnospiraceae
Lactobacillaceae
Micrococcaceae
Pasteurellaceae
Peptostreptococcaceae
Porphyromonadaceae
Ruminococcaceae
Staphylococcaceae
Streptococcaceae
Veillonellaceae
undef

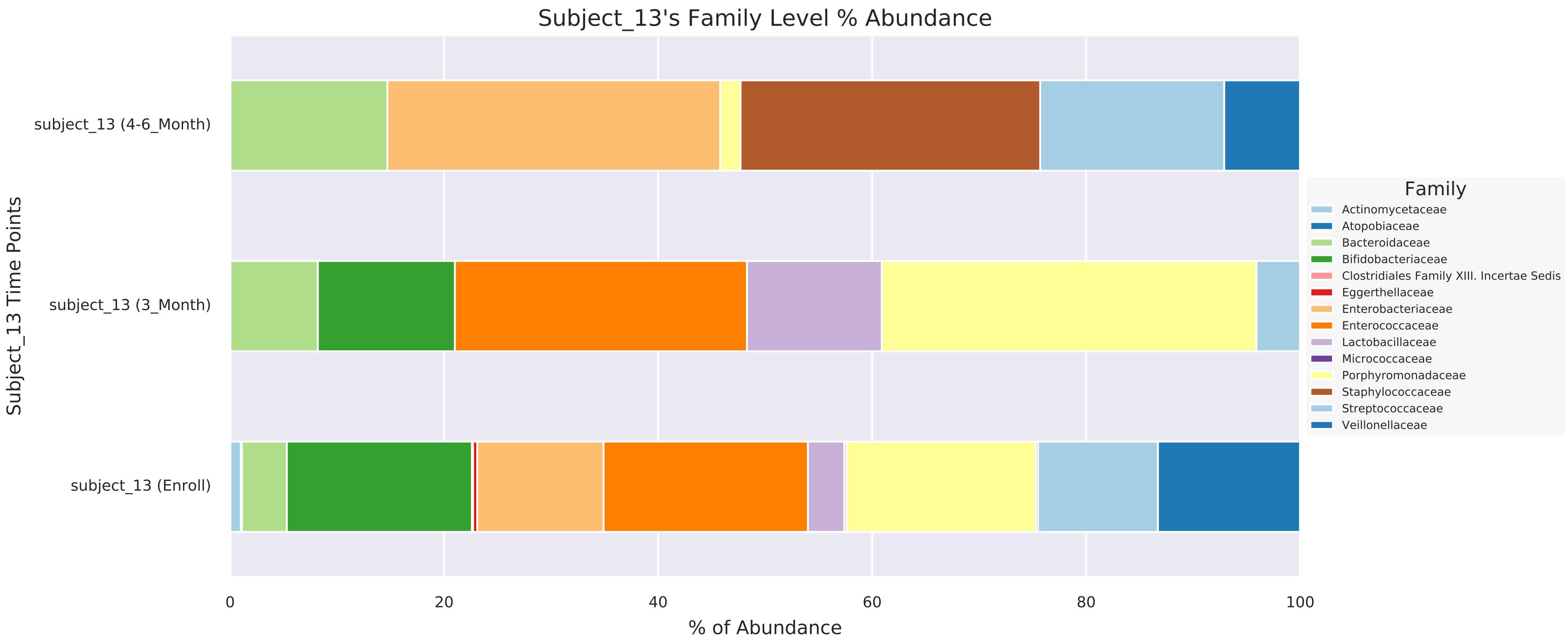

Subject_13's Family Level % Abundance
subject_13 (4-6_Month)
subject_13 (3_Month)
subject_13 (Enroll)
Subject_13 Time Points
0
20
40
60
80
100
% of Abundance
Family
Actinomycetaceae
Atopobiaceae
Bacteroidaceae
Bifidobacteriaceae
Clostridiales Family XIII. Incertae Sedis
Eggerthellaceae
Enterobacteriaceae
Enterococcaceae
Lactobacillaceae
Micrococcaceae
Porphyromonadaceae
Staphylococcaceae
Streptococcaceae
Veillonellaceae

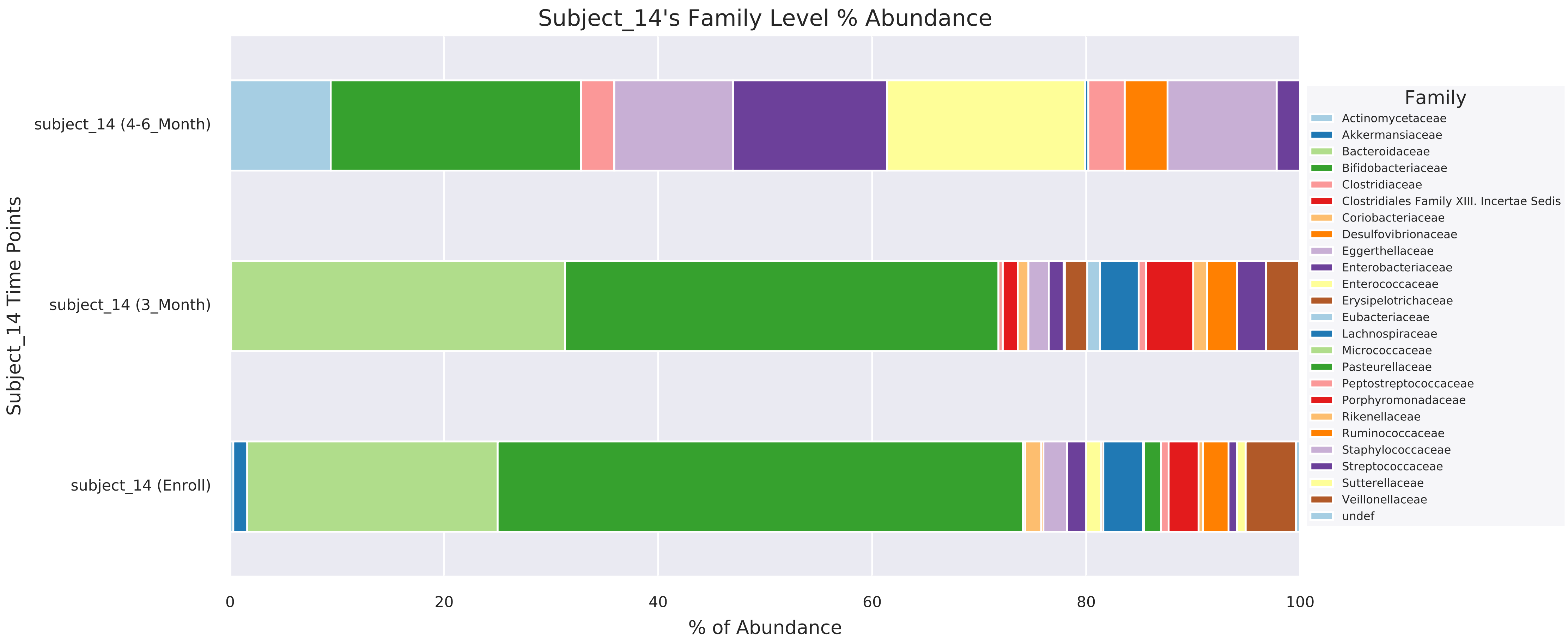

Subject_14's Family Level % Abundance
Subject_14 Time Points
subject_14 (4-6_Month)
subject_14 (3_Month)
subject_14 (Enroll)
0
20
40
60
80
100
% of Abundance
Family
Actinomycetaceae
Akkermansiaceae
Bacteroidaceae
Bifidobacteriaceae
Clostridiaceae
Clostridiales Family XIII. Incertae Sedis
Coriobacteriaceae
Desulfovibrionaceae
Eggerthellaceae
Enterobacteriaceae
Enterococcaceae
Erysipelotrichaceae
Eubacteriaceae
Lachnospiraceae
Micrococcaceae
Pasteurellaceae
Peptostreptococcaceae
Porphyromonadaceae
Rikenellaceae
Ruminococcaceae
Staphylococcaceae
Streptococcaceae
Sutterellaceae
Veillonellaceae
undef

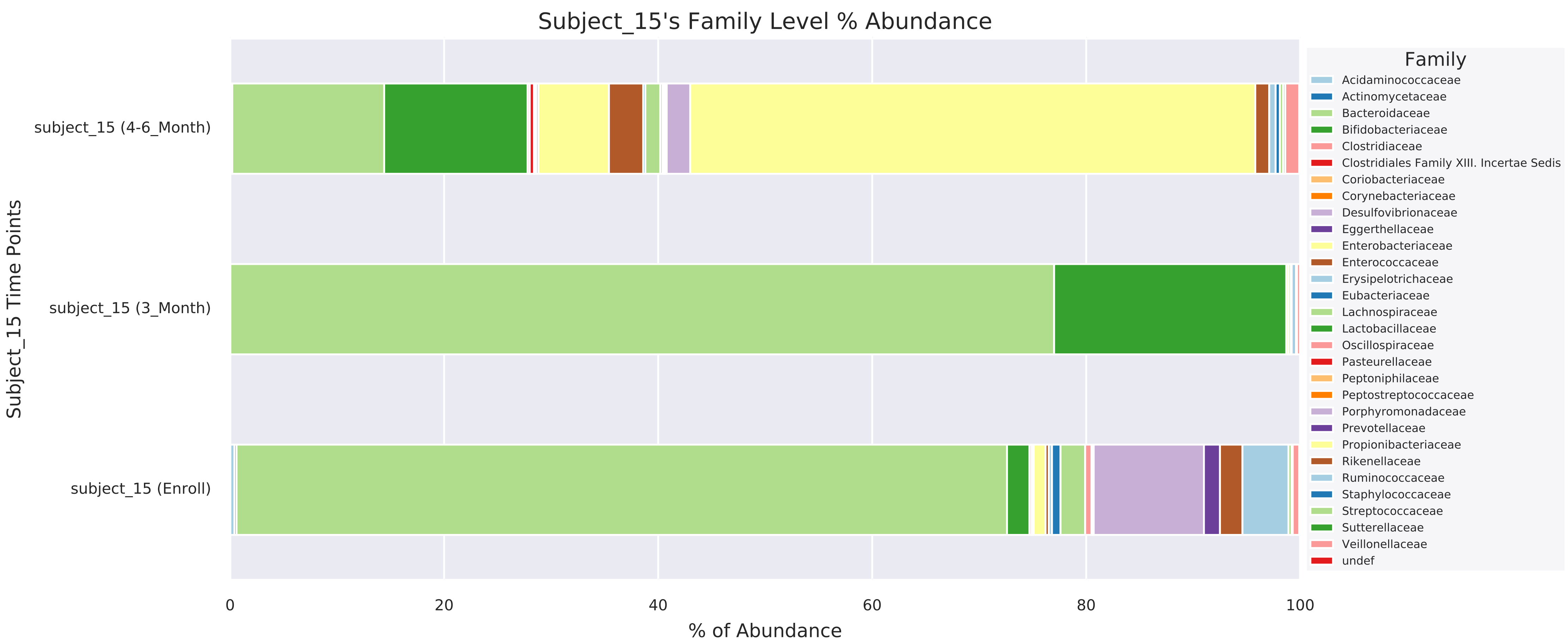
Subject_15's Family Level % Abundance
Subject_15 Time Points
subject_15 (4-6_Month)
subject_15 (3_Month)
subject_15 (Enroll)
% of Abundance
0
20
40
60
80
100
Family
Acidaminococcaceae
Actinomycetaceae
Bacteroidaceae
Bifidobacteriaceae
Clostridiaceae
Clostridiales Family XIII. Incertae Sedis
Coriobacteriaceae
Corynebacteriaceae
Desulfovibrionaceae
Eggerthellaceae
Enterobacteriaceae
Enterococcaceae
Erysipelotrichaceae
Eubacteriaceae
Lachnospiraceae
Lactobacillaceae
Oscillospiraceae
Pasteurellaceae
Peptoniphilaceae
Peptostreptococcaceae
Porphyromonadaceae
Prevotellaceae
Propionibacteriaceae
Rikenellaceae
Ruminococcaceae
Staphylococcaceae
Streptococcaceae
Sutterellaceae
Veillonellaceae
undef

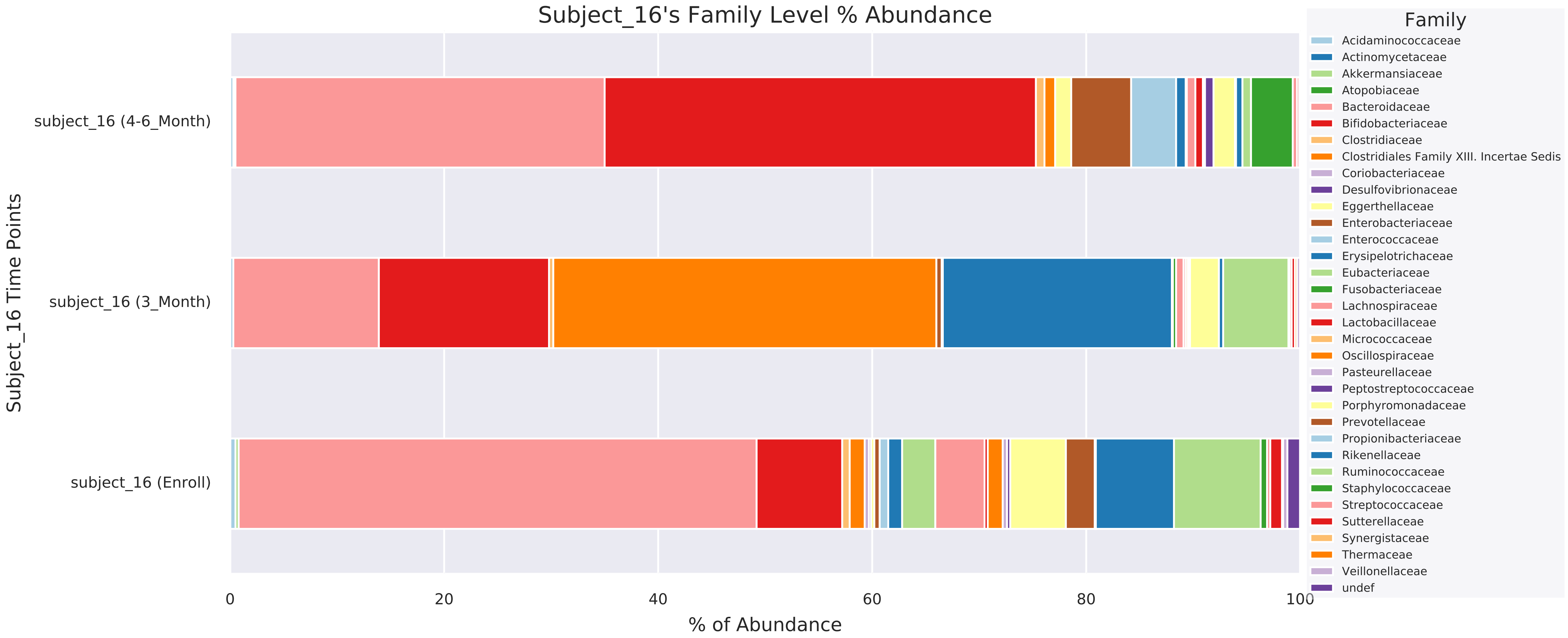

Subject_16's Family Level % Abundance
subject_16 (4-6_Month)
subject_16 (3_Month)
subject_16 (Enroll)
Subject_16 Time Points
0
20
40
60
80
100
% of Abundance
Family
Acidaminococcaceae
Actinomycetaceae
Akkermansiaceae
Atopobiaceae
Bacteroidaceae
Bifidobacteriaceae
Clostridiaceae
Clostridiales Family XIII. Incertae Sedis
Coriobacteriaceae
Desulfovibrionaceae
Eggerthellaceae
Enterobacteriaceae
Enterococcaceae
Erysipelotrichaceae
Eubacteriaceae
Fusobacteriaceae
Lachnospiraceae
Lactobacillaceae
Micrococcaceae
Oscillospiraceae
Pasteurellaceae
Peptostreptococcaceae
Porphyromonadaceae
Prevotellaceae
Propionibacteriaceae
Rikenellaceae
Ruminococcaceae
Staphylococcaceae
Streptococcaceae
Sutterellaceae
Synergistaceae
Thermaceae
Veillonellaceae
undef

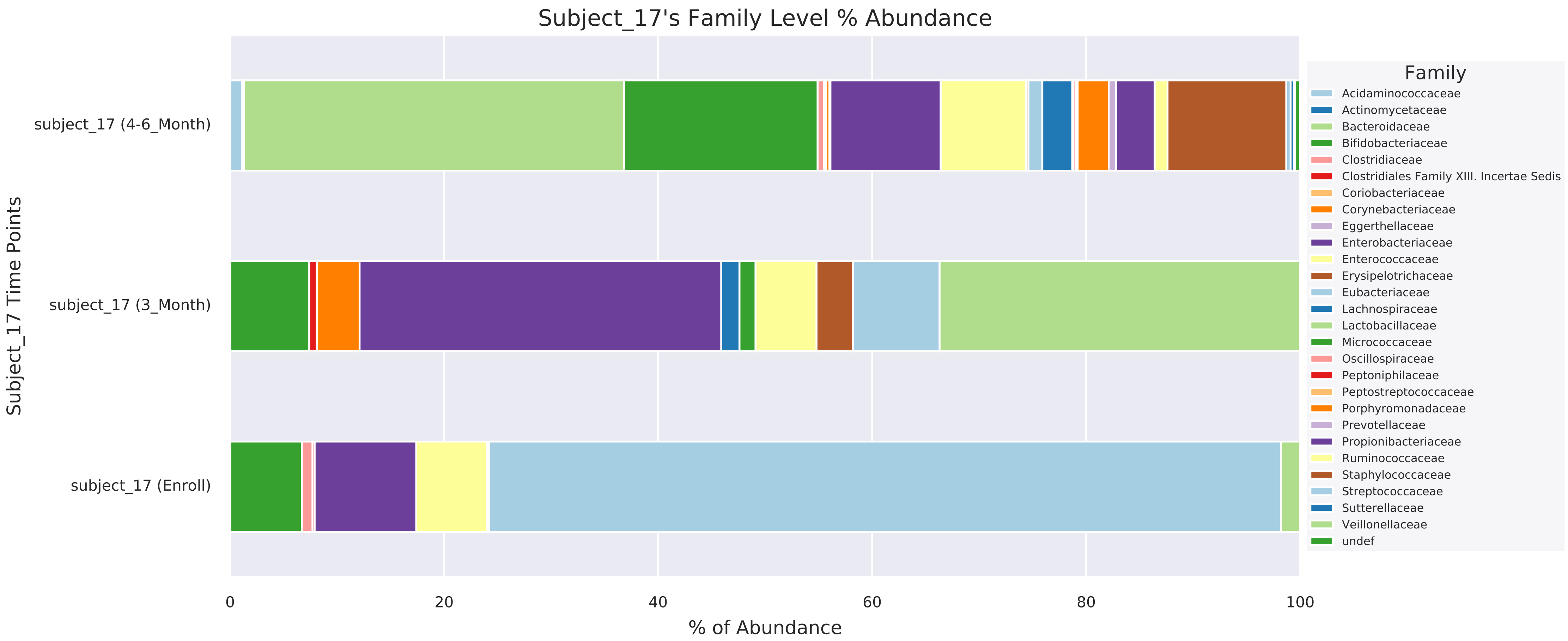
Subject_17's Family Level % Abundance
subject_17 (4-6_Month)
subject_17 (3_Month)
subject_17 (Enroll)
Subject_17 Time Points
0
20
40
60
80
100
% of Abundance
Family
Acidaminococcaceae
Actinomycetaceae
Bacteroidaceae
Bifidobacteriaceae
Clostridiaceae
Clostridiales Family XIII. Incertae Sedis
Coriobacteriaceae
Corynebacteriaceae
Eggerthellaceae
Enterobacteriaceae
Enterococcaceae
Erysipelotrichaceae
Eubacteriaceae
Lachnospiraceae
Lactobacillaceae
Micrococcaceae
Oscillospiraceae
Peptoniphilaceae
Peptostreptococcaceae
Porphyromonadaceae
Prevotellaceae
Propionibacteriaceae
Ruminococcaceae
Staphylococcaceae
Streptococcaceae
Sutterellaceae
Veillonellaceae
undef

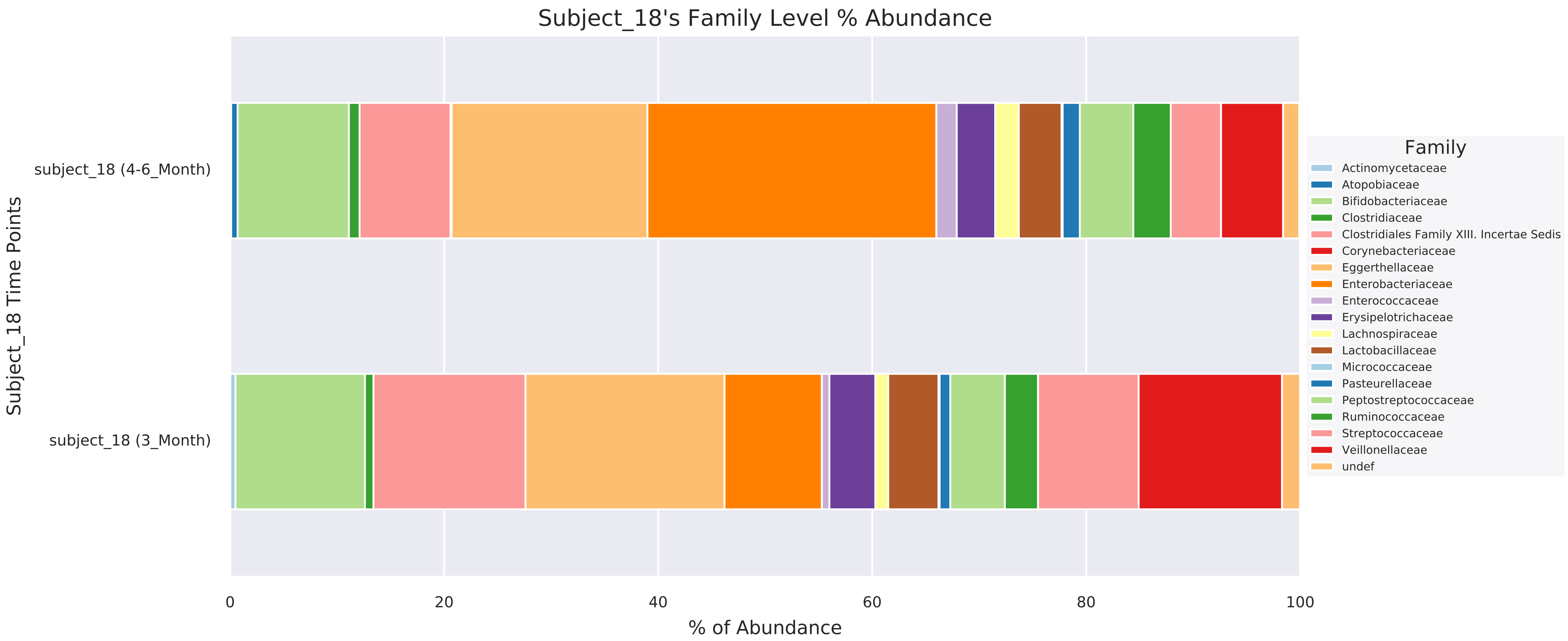

Subject_18's Family Level % Abundance
Subject_18 Time Points
subject_18 (4-6_Month)
subject_18 (3_Month)
0
20
40
60
80
100
% of Abundance
Family
Actinomycetaceae
Atopobiaceae
Bifidobacteriaceae
Clostridiaceae
Clostridiales Family XIII. Incertae Sedis
Corynebacteriaceae
Eggerthellaceae
Enterobacteriaceae
Enterococcaceae
Erysipelotrichaceae
Lachnospiraceae
Lactobacillaceae
Micrococcaceae
Pasteurellaceae
Peptostreptococcaceae
Ruminococcaceae
Streptococcaceae
Veillonellaceae
undef

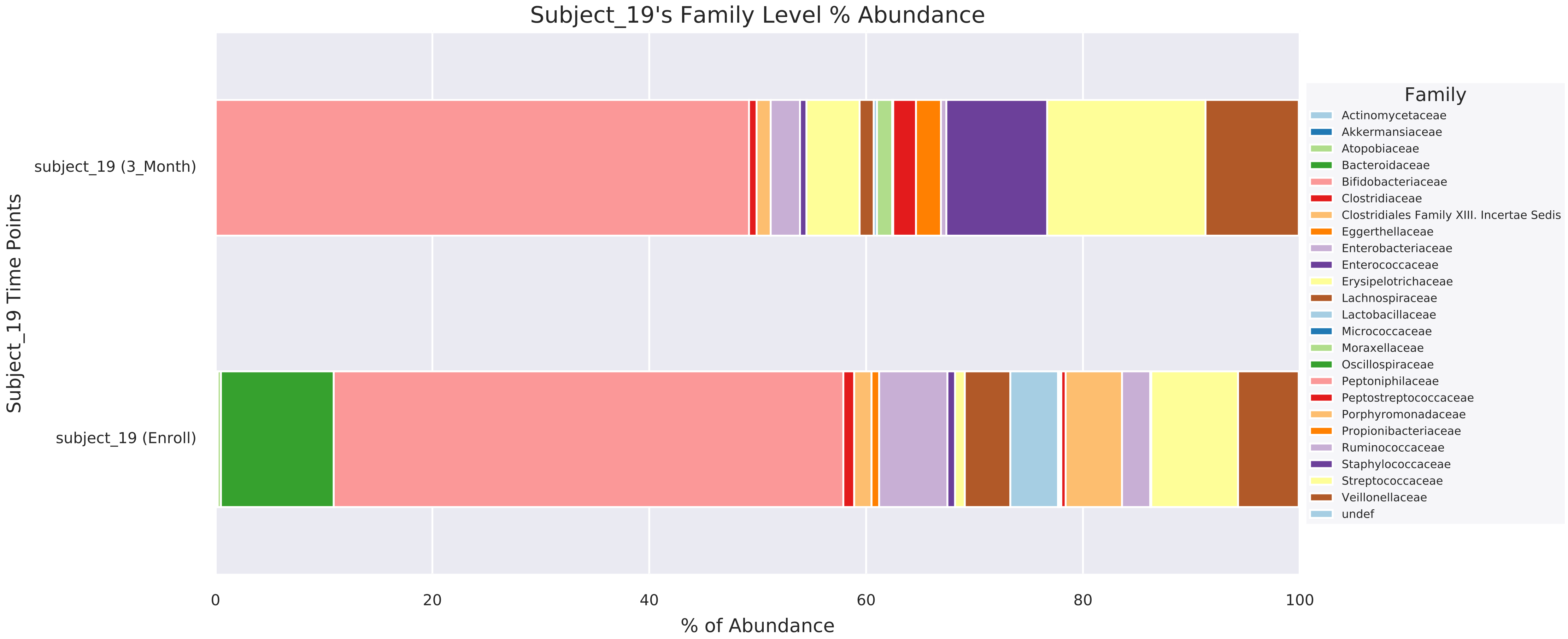

Subject_19's Family Level % Abundance
Subject_19 Time Points
subject_19 (3_Month)
subject_19 (Enroll)
0
20
40
60
80
100
% of Abundance
Family
Actinomycetaceae
Akkermansiaceae
Atopobiaceae
Bacteroidaceae
Bifidobacteriaceae
Clostridiaceae
Clostridiales Family XIII. Incertae Sedis
Eggerthellaceae
Enterobacteriaceae
Enterococcaceae
Erysipelotrichaceae
Lachnospiraceae
Lactobacillaceae
Micrococcaceae
Moraxellaceae
Oscillospiraceae
Peptoniphilaceae
Peptostreptococcaceae
Porphyromonadaceae
Propionibacteriaceae
Ruminococcaceae
Staphylococcaceae
Streptococcaceae
Veillonellaceae
undef

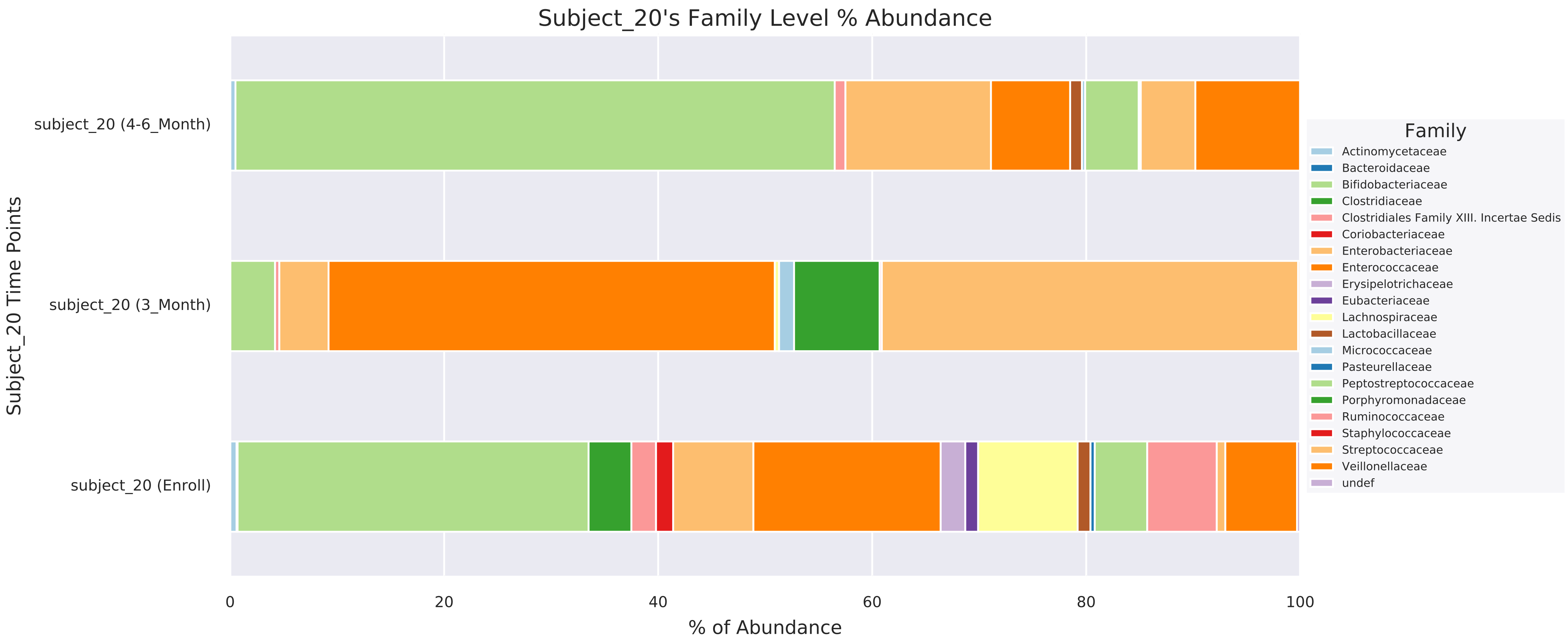
Subject_20's Family Level % Abundance
Subject_20 Time Points
subject_20 (4-6_Month)
subject_20 (3_Month)
subject_20 (Enroll)
0
20
40
60
80
100
% of Abundance
Family
Actinomycetaceae
Bacteroidaceae
Bifidobacteriaceae
Clostridiaceae
Clostridiales Family XIII. Incertae Sedis
Coriobacteriaceae
Enterobacteriaceae
Enterococcaceae
Erysipelotrichaceae
Eubacteriaceae
Lachnospiraceae
Lactobacillaceae
Micrococcaceae
Pasteurellaceae
Peptostreptococcaceae
Porphyromonadaceae
Ruminococcaceae
Staphylococcaceae
Streptococcaceae
Veillonellaceae
undef

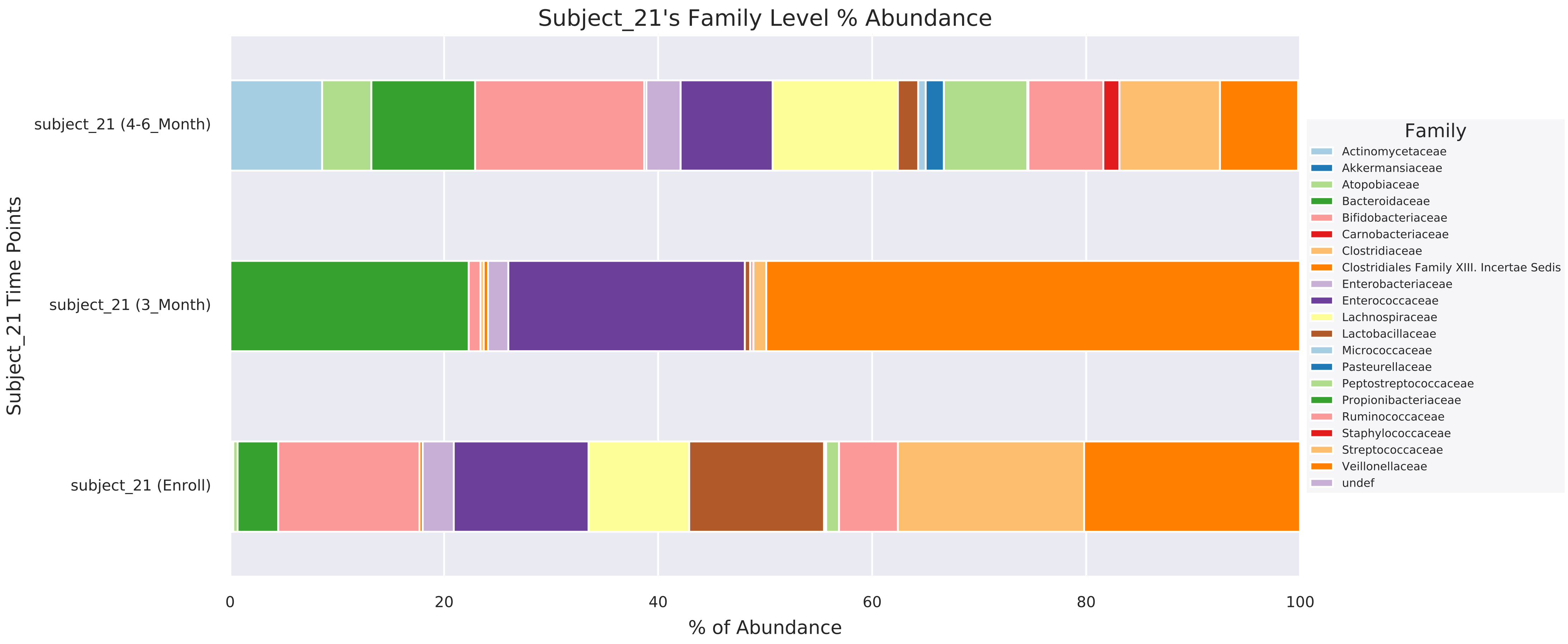
Subject_21's Family Level % Abundance
Subject_21 Time Points
subject_21 (4-6_Month)
subject_21 (3_Month)
subject_21 (Enroll)
0
20
40
60
80
100
% of Abundance
Family
Actinomycetaceae
Akkermansiaceae
Atopobiaceae
Bacteroidaceae
Bifidobacteriaceae
Carnobacteriaceae
Clostridiaceae
Clostridiales Family XIII. Incertae Sedis
Enterobacteriaceae
Enterococcaceae
Lachnospiraceae
Lactobacillaceae
Micrococcaceae
Pasteurellaceae
Peptostreptococcaceae
Propionibacteriaceae
Ruminococcaceae
Staphylococcaceae
Streptococcaceae
Veillonellaceae
undef

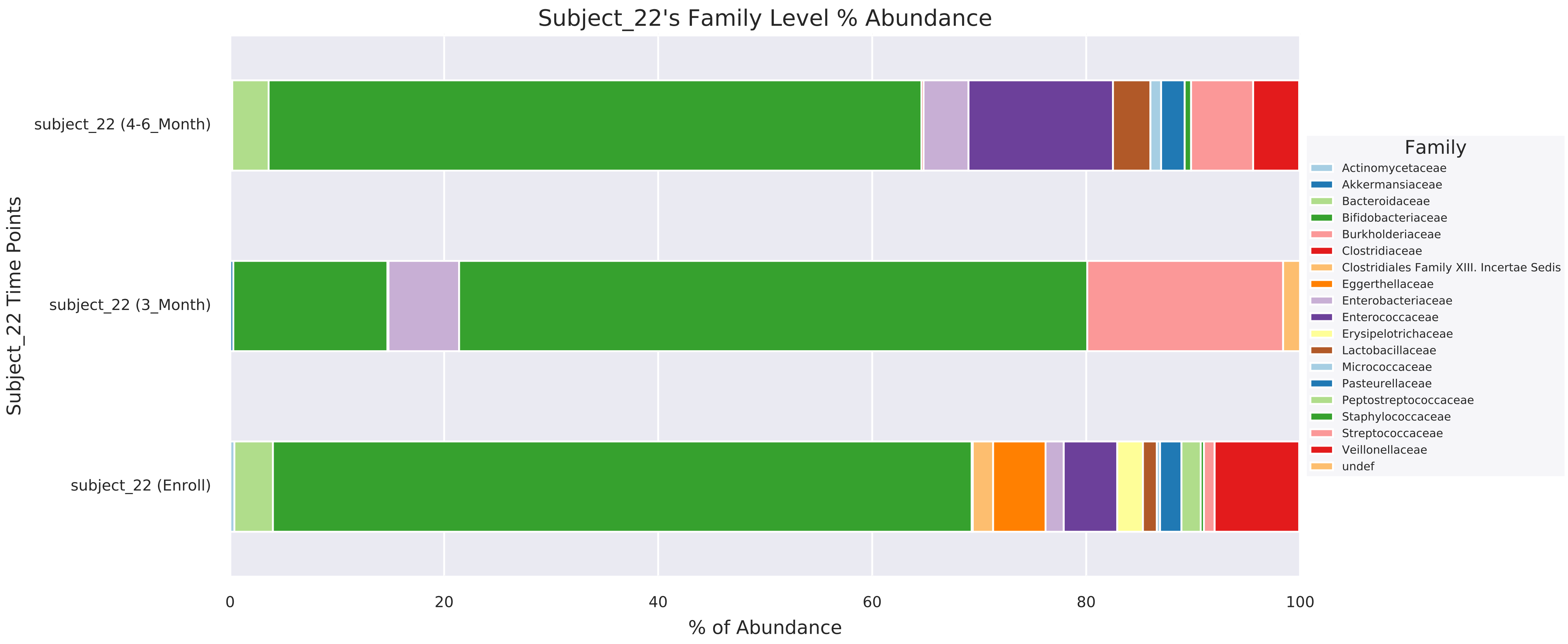

Subject_22's Family Level % Abundance
Subject_22 Time Points
subject_22 (4-6_Month)
subject_22 (3_Month)
subject_22 (Enroll)
0
20
40
60
80
100
% of Abundance
Family
Actinomycetaceae
Akkermansiaceae
Bacteroidaceae
Bifidobacteriaceae
Burkholderiaceae
Clostridiaceae
Clostridiales Family XIII. Incertae Sedis
Eggerthellaceae
Enterobacteriaceae
Enterococcaceae
Erysipelotrichaceae
Lactobacillaceae
Micrococcaceae
Pasteurellaceae
Peptostreptococcaceae
Staphylococcaceae
Streptococcaceae
Veillonellaceae
undef

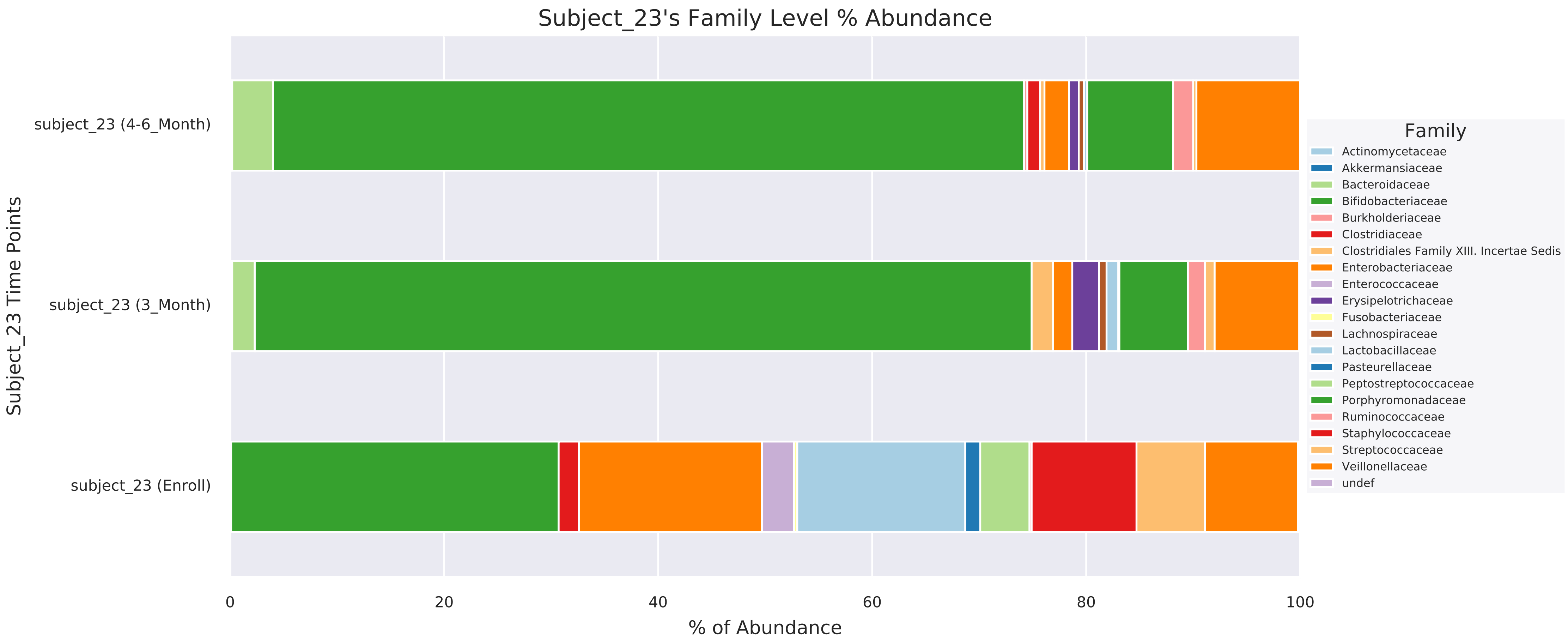
Subject_23's Family Level % Abundance
Subject_23 Time Points
subject_23 (4-6_Month)
subject_23 (3_Month)
subject_23 (Enroll)
0
20
40
60
80
100
% of Abundance
Family
Actinomycetaceae
Akkermansiaceae
Bacteroidaceae
Bifidobacteriaceae
Burkholderiaceae
Clostridiaceae
Clostridiales Family XIII. Incertae Sedis
Enterobacteriaceae
Enterococcaceae
Erysipelotrichaceae
Fusobacteriaceae
Lachnospiraceae
Lactobacillaceae
Pasteurellaceae
Peptostreptococcaceae
Porphyromonadaceae
Ruminococcaceae
Staphylococcaceae
Streptococcaceae
Veillonellaceae
undef

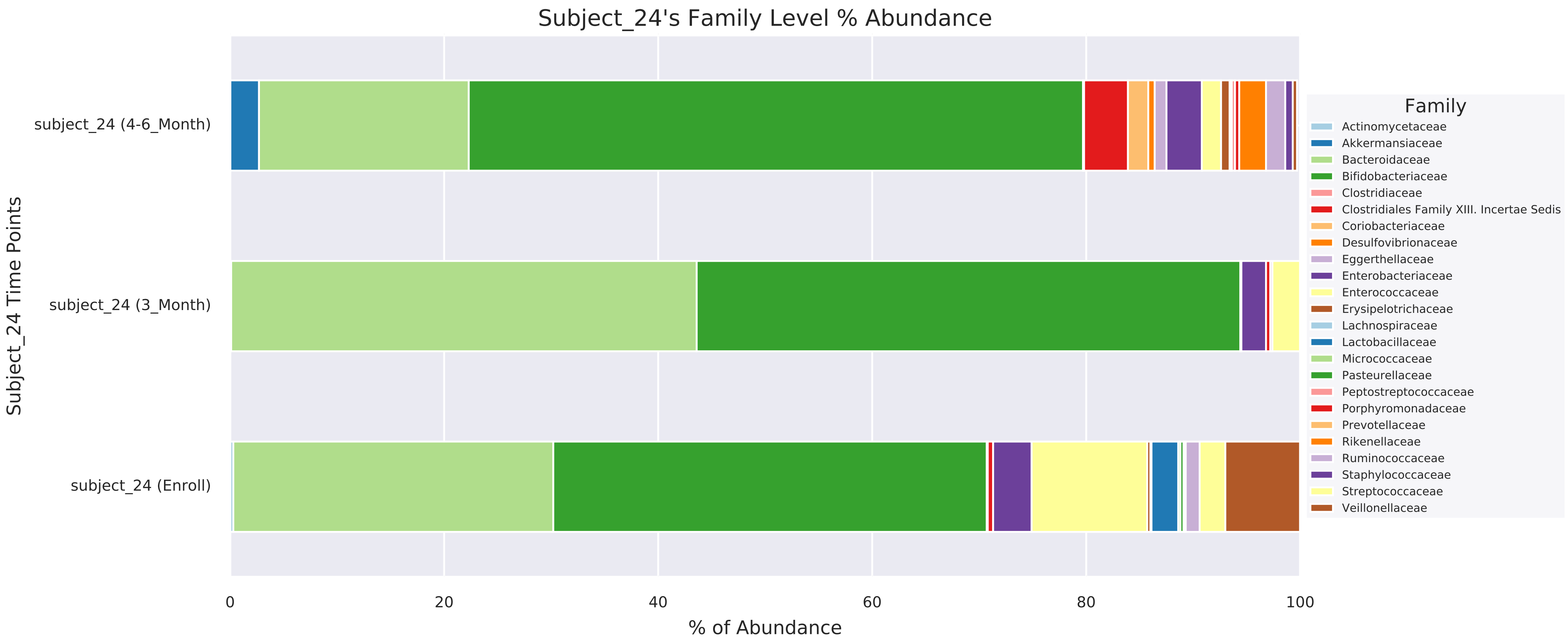

Subject_24's Family Level % Abundance
Subject_24 Time Points
subject_24 (4-6_Month)
subject_24 (3_Month)
subject_24 (Enroll)
0
20
40
60
80
100
% of Abundance
Family
Actinomycetaceae
Akkermansiaceae
Bacteroidaceae
Bifidobacteriaceae
Clostridiaceae
Clostridiales Family XIII. Incertae Sedis
Coriobacteriaceae
Desulfovibrionaceae
Eggerthellaceae
Enterobacteriaceae
Enterococcaceae
Erysipelotrichaceae
Lachnospiraceae
Lactobacillaceae
Micrococcaceae
Pasteurellaceae
Peptostreptococcaceae
Porphyromonadaceae
Prevotellaceae
Rikenellaceae
Ruminococcaceae
Staphylococcaceae
Streptococcaceae
Veillonellaceae

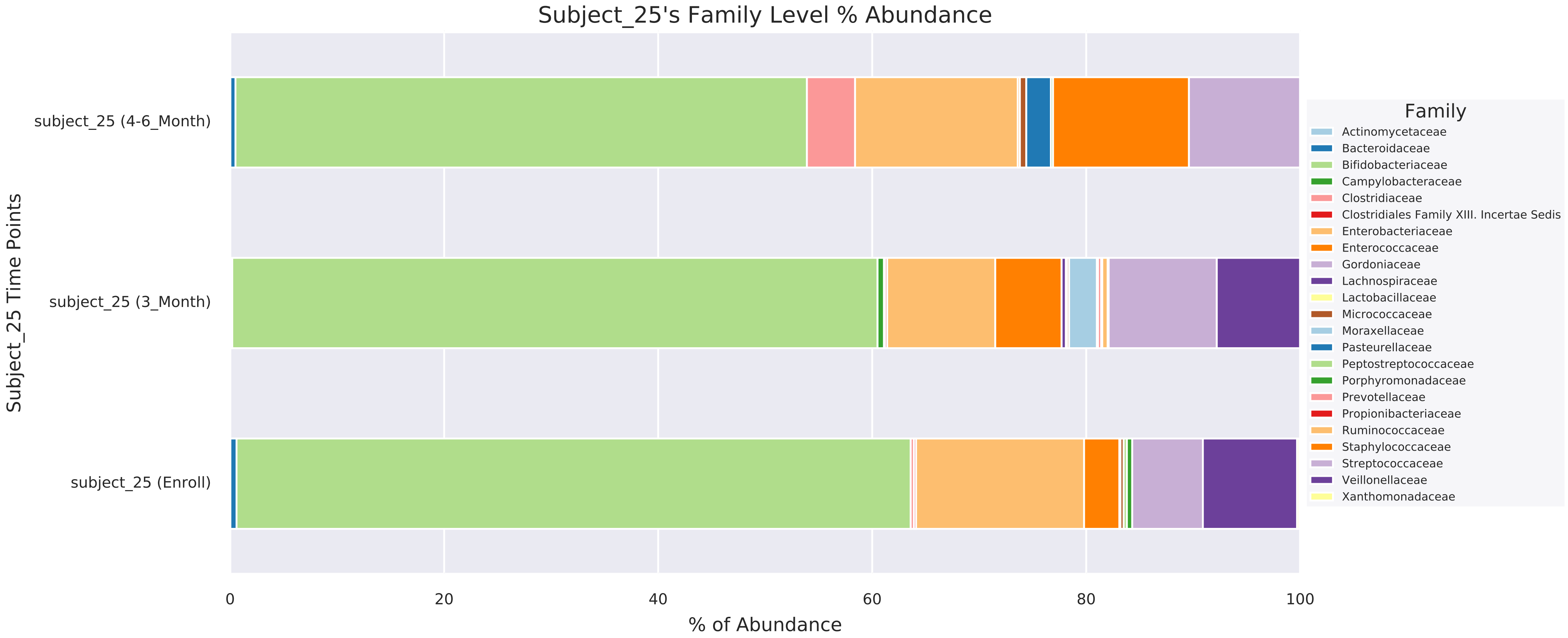
Subject_25's Family Level % Abundance
subject_25 (4-6_Month)
subject_25 (3_Month)
subject_25 (Enroll)
Subject_25 Time Points
0
20
40
60
80
100
% of Abundance
Family
Actinomycetaceae
Bacteroidaceae
Bifidobacteriaceae
Campylobacteraceae
Clostridiaceae
Clostridiales Family XIII. Incertae Sedis
Enterobacteriaceae
Enterococcaceae
Gordoniaceae
Lachnospiraceae
Lactobacillaceae
Micrococcaceae
Moraxellaceae
Pasteurellaceae
Peptostreptococcaceae
Porphyromonadaceae
Prevotellaceae
Propionibacteriaceae
Ruminococcaceae
Staphylococcaceae
Streptococcaceae
Veillonellaceae
Xanthomonadaceae

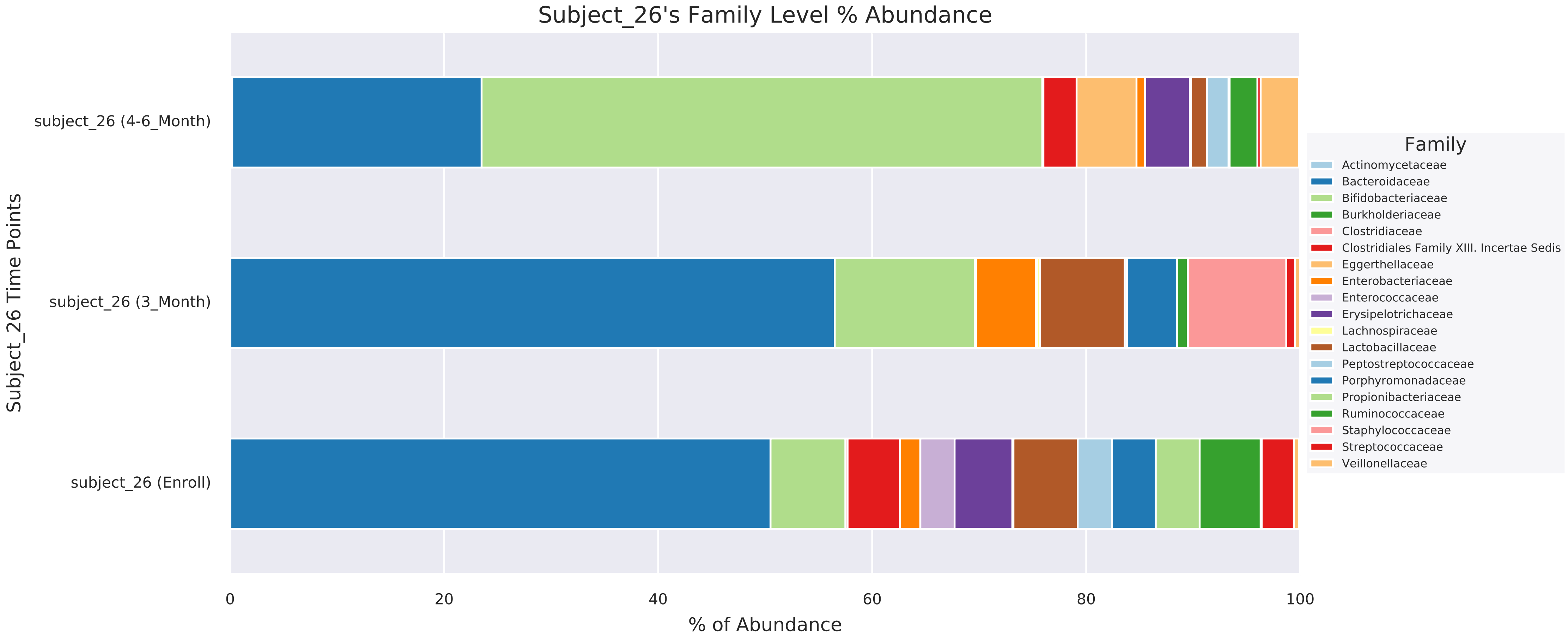
Subject_26's Family Level % Abundance
subject_26 (4-6_Month)
subject_26 (3_Month)
subject_26 (Enroll)
Subject_26 Time Points
0
20
40
60
80
100
% of Abundance
Family
Actinomycetaceae
Bacteroidaceae
Bifidobacteriaceae
Burkholderiaceae
Clostridiaceae
Clostridiales Family XIII. Incertae Sedis
Eggerthellaceae
Enterobacteriaceae
Enterococcaceae
Erysipelotrichaceae
Lachnospiraceae
Lactobacillaceae
Peptostreptococcaceae
Porphyromonadaceae
Propionibacteriaceae
Ruminococcaceae
Staphylococcaceae
Streptococcaceae
Veillonellaceae

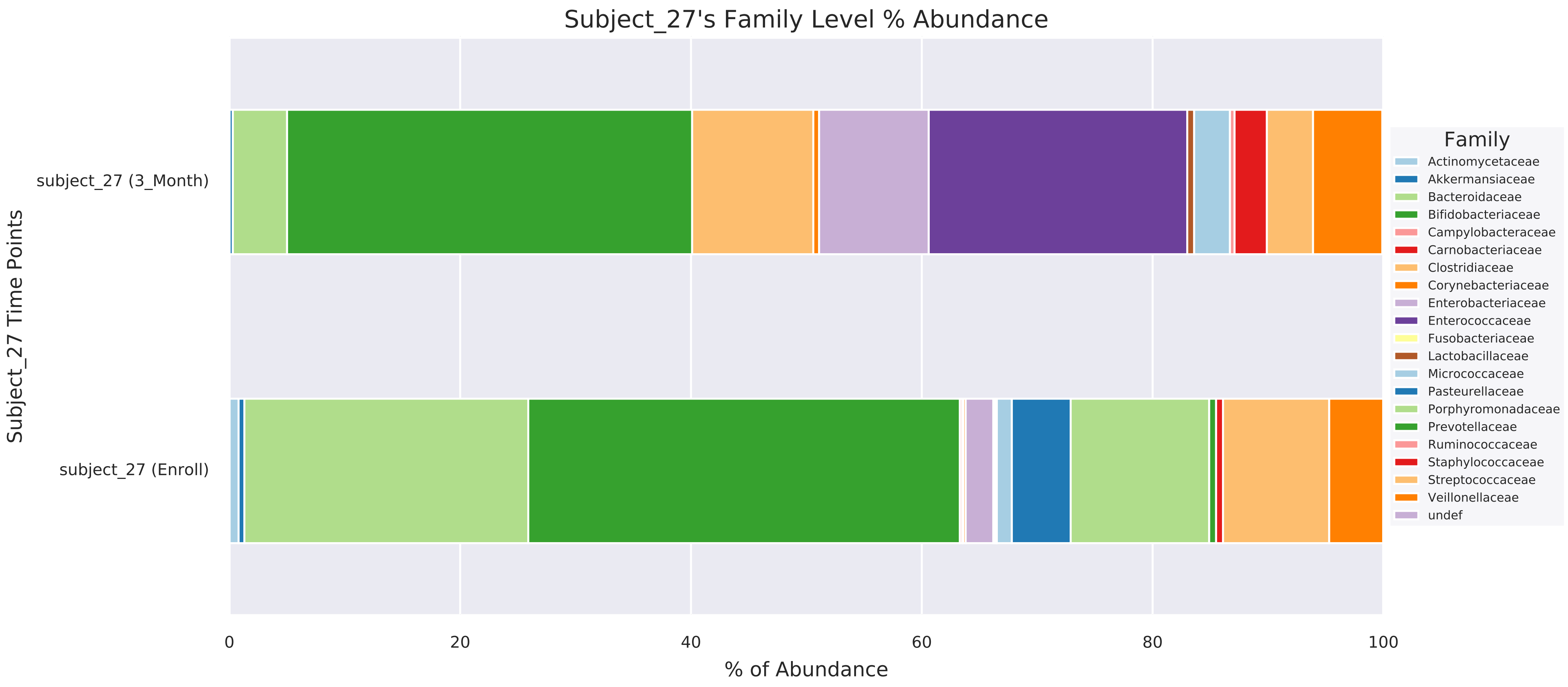

Subject_27's Family Level % Abundance
Subject_27 Time Points
subject_27 (3_Month)
subject_27 (Enroll)
% of Abundance
0
20
40
60
80
100
Family
Actinomycetaceae
Akkermansiaceae
Bacteroidaceae
Bifidobacteriaceae
Campylobacteraceae
Carnobacteriaceae
Clostridiaceae
Corynebacteriaceae
Enterobacteriaceae
Enterococcaceae
Fusobacteriaceae
Lactobacillaceae
Micrococcaceae
Pasteurellaceae
Porphyromonadaceae
Prevotellaceae
Ruminococcaceae
Staphylococcaceae
Streptococcaceae
Veillonellaceae
undef

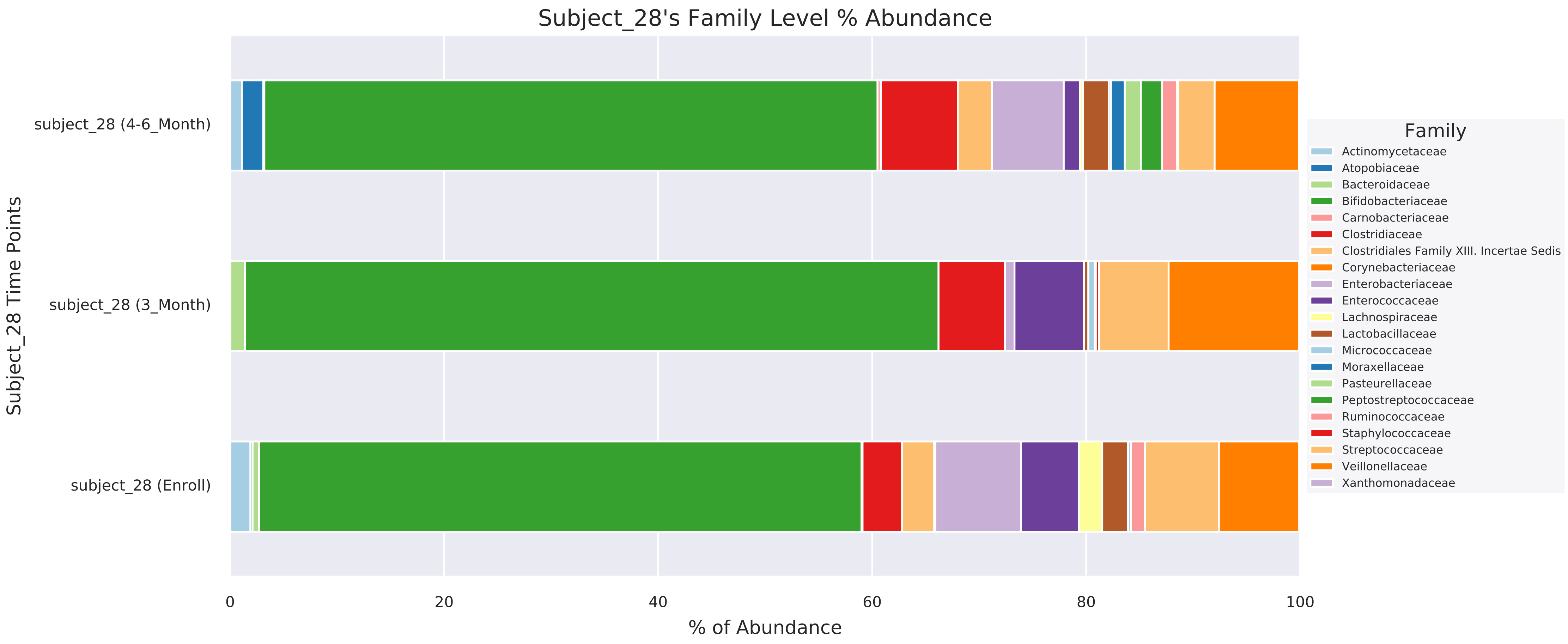

Subject_28's Family Level % Abundance
Subject_28 Time Points
subject_28 (4-6_Month)
subject_28 (3_Month)
subject_28 (Enroll)
0
20
40
60
80
100
% of Abundance
Family
Actinomycetaceae
Atopobiaceae
Bacteroidaceae
Bifidobacteriaceae
Carnobacteriaceae
Clostridiaceae
Clostridiales Family XIII. Incertae Sedis
Corynebacteriaceae
Enterobacteriaceae
Enterococcaceae
Lachnospiraceae
Lactobacillaceae
Micrococcaceae
Moraxellaceae
Pasteurellaceae
Peptostreptococcaceae
Ruminococcaceae
Staphylococcaceae
Streptococcaceae
Veillonellaceae
Xanthomonadaceae

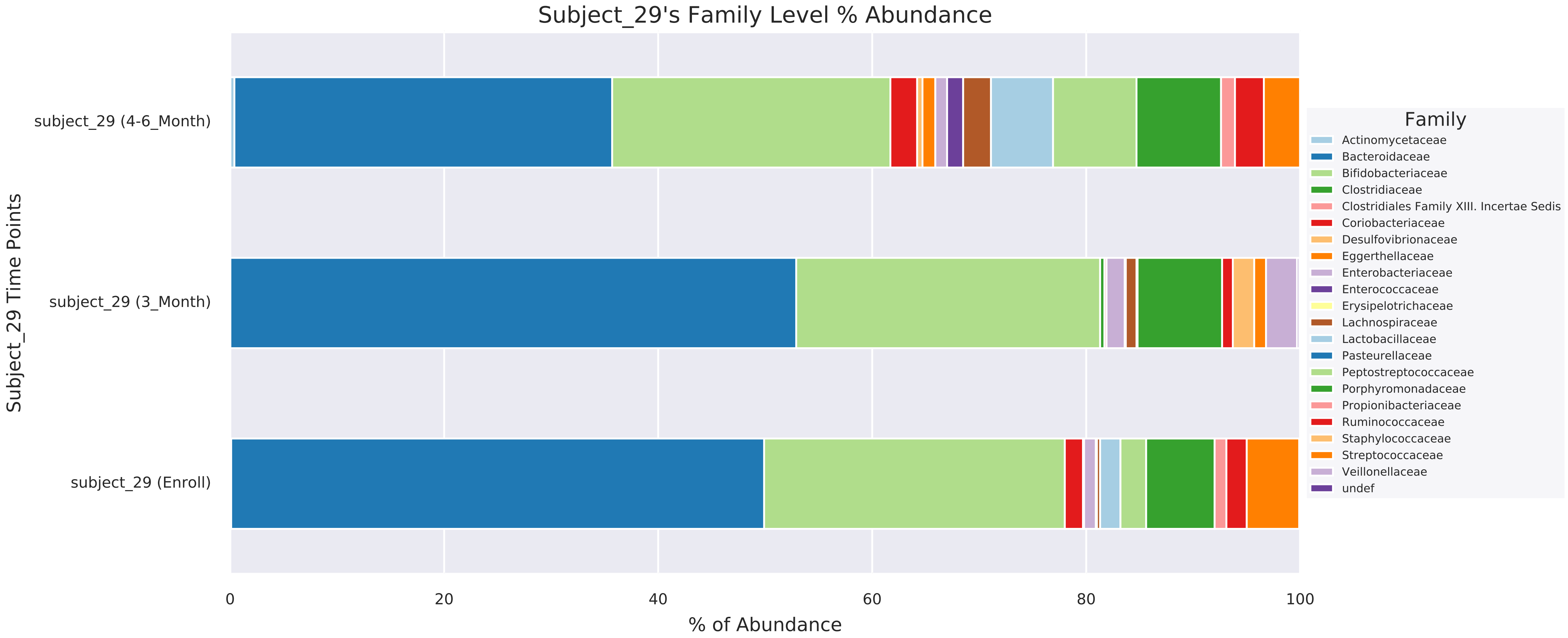
Subject_29's Family Level % Abundance
subject_29 (4-6_Month)
subject_29 (3_Month)
subject_29 (Enroll)
Subject_29 Time Points
0
20
40
60
80
100
% of Abundance
Family
Actinomycetaceae
Bacteroidaceae
Bifidobacteriaceae
Clostridiaceae
Clostridiales Family XIII. Incertae Sedis
Coriobacteriaceae
Desulfovibrionaceae
Eggerthellaceae
Enterobacteriaceae
Enterococcaceae
Erysipelotrichaceae
Lachnospiraceae
Lactobacillaceae
Pasteurellaceae
Peptostreptococcaceae
Porphyromonadaceae
Propionibacteriaceae
Ruminococcaceae
Staphylococcaceae
Streptococcaceae
Veillonellaceae
undef

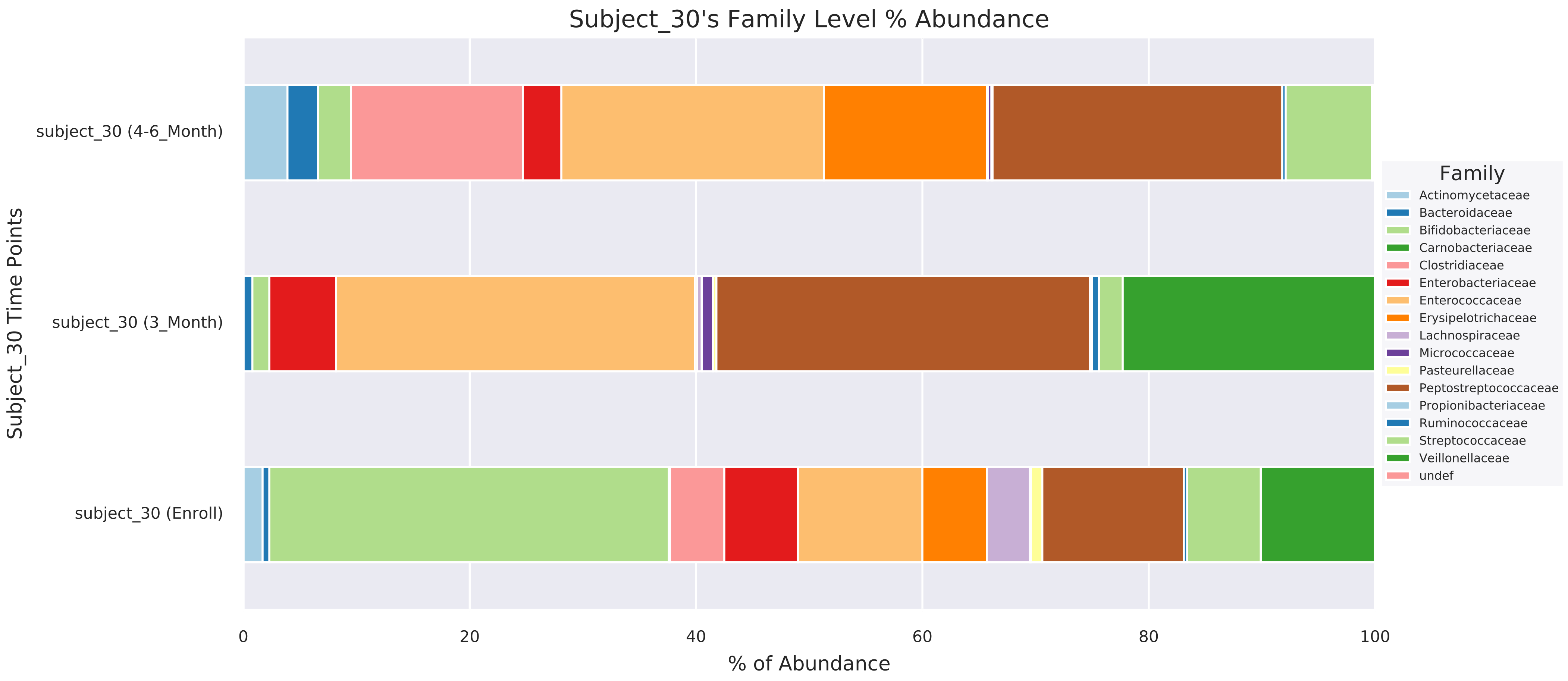
Subject_30's Family Level % Abundance
subject_30 (4-6_Month)
subject_30 (3_Month)
subject_30 (Enroll)
Subject_30 Time Points
0
20
40
60
80
100
% of Abundance
Family
Actinomycetaceae
Bacteroidaceae
Bifidobacteriaceae
Carnobacteriaceae
Clostridiaceae
Enterobacteriaceae
Enterococcaceae
Erysipelotrichaceae
Lachnospiraceae
Micrococcaceae
Pasteurellaceae
Peptostreptococcaceae
Propionibacteriaceae
Ruminococcaceae
Streptococcaceae
Veillonellaceae
undef

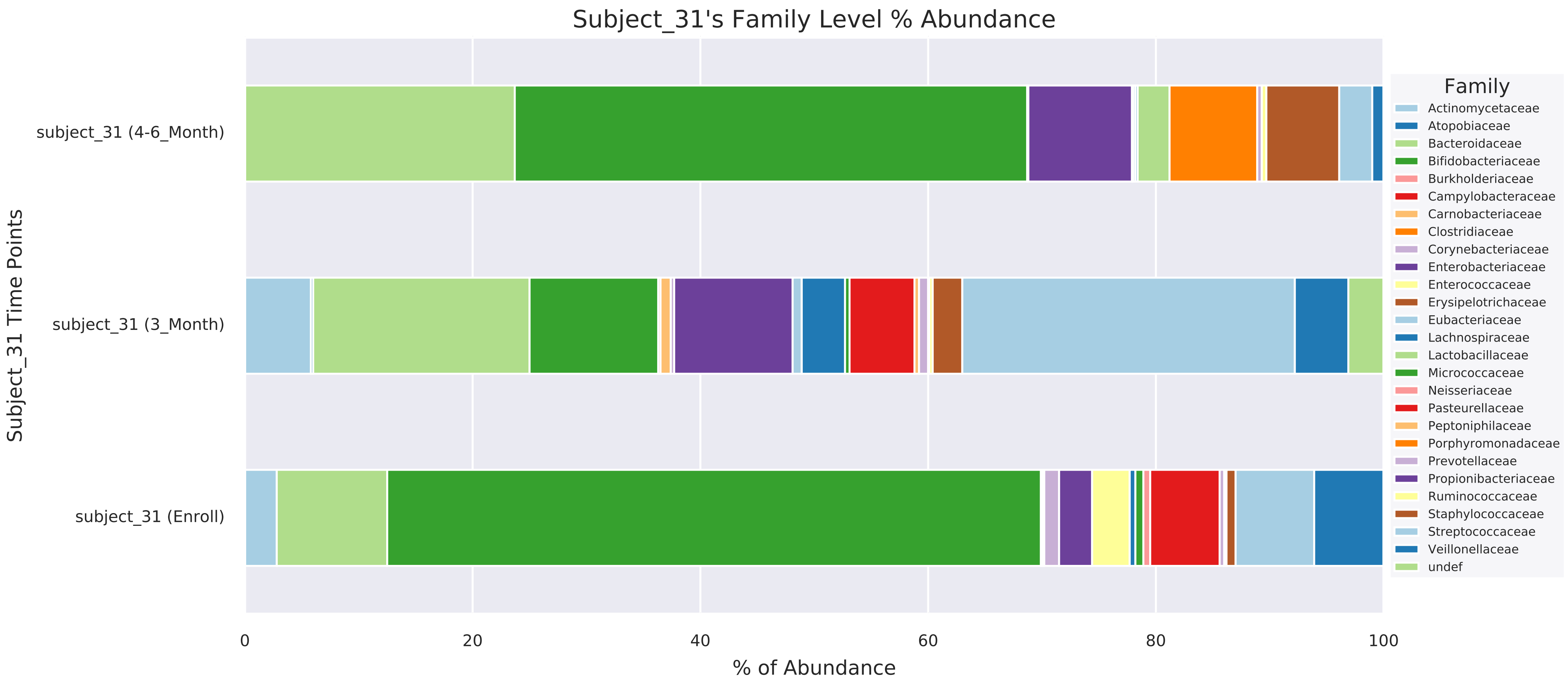

Subject_31's Family Level % Abundance
Subject_31 Time Points
subject_31 (4-6_Month)
subject_31 (3_Month)
subject_31 (Enroll)
0
20
40
60
80
100
% of Abundance
Family
Actinomycetaceae
Atopobiaceae
Bacteroidaceae
Bifidobacteriaceae
Burkholderiaceae
Campylobacteraceae
Carnobacteriaceae
Clostridiaceae
Corynebacteriaceae
Enterobacteriaceae
Enterococcaceae
Erysipelotrichaceae
Eubacteriaceae
Lachnospiraceae
Lactobacillaceae
Micrococcaceae
Neisseriaceae
Pasteurellaceae
Peptoniphilaceae
Porphyromonadaceae
Prevotellaceae
Propionibacteriaceae
Ruminococcaceae
Staphylococcaceae
Streptococcaceae
Veillonellaceae
undef
