# Supplementary material for: Multi-omics analysis reveals the influence of genetic and environmental risk factors on developing gut microbiota in infants at risk of celiac disease
Source: Microbiome. 2020 Sep 11;8:130. doi: 10.1186/s40168-020-00906-w (PMC7488762; doi:10.1186/s40168-020-00906-w)
Supplement: Supplementary file 7 — Additional file 6: Supplementary text describing details of data analysis methods. [file 40168_2020_906_MOESM6_ESM.zip › AdditionalFile6.docx]

# Supplementary text for

**Multi-omics analysis reveals the influence of genetic and environmental risk factors on developing gut microbiota in infants at risk of celiac disease**

Leonard, Maureen M; Karathia^*^, Hiren; Pujolassos^*^, Meritxell; Troisi^*^, Jacopo; Valitutti^*^, Francesco; Subramanian^*^, Poorani; Camhi, Stephanie; Kenyon, Victoria; Colucci, Angelo; Serena, Gloria; Cucchiara, Salvatore; Montuori, Monica; Malamisura, Basilio; Francavilla, Ruggiero; Elli, Luca; Fanelli, Brian; Colwell, Rita; Hasan, Nur; Zomorrodi^#^, Ali R.; Fasano^#^, Alessio

Table of Contents

[Supplementary text for 1](#_Toc42698899)

[Taxonomic profiling using the CosmosID’s metagenomic analysis platform 1](#_Toc42698900)

[Metabolomics profiling and analysis details 2](#_Toc42698901)

[Association analysis 3](#_Toc42698902)

[References 4](#_Toc42698903)

## Taxonomic profiling using the CosmosID’s metagenomic analysis platform

Metagenomic sequencing reads were analyzed by using CosmosID’s (CosmosID Inc., Rockville, MD) commercial metagenomic analysis platform (formerly knowns as GENIUS; https://app.cosmosid.com/login) [1, 2] to reveal the associated microbial community composition up to the species-level resolution. Briefly, this platform utilizes a comprehensive and highly curated microbial genomic database (GenBook®) and a high performance data-mining $k$-mer algorithm.

**The GeneBook**® **database**: This reference database constitutes both genomes or gene sequences publicly available through NCBI- RefSeq/WGS/SRA/nr, PATRIC, M5NR, IMG, ENA, DDBJ, CARD, ResFinder, ARDB, ARG-ANNOT, mvirdb, VFDB etc., as well as additional genomes sequenced by CosmosID and its collaborators. At the time of this submission, the total number of genomes and gene sequences in the GenBook database was 189,105, which includes over 1000 bacterial, 5000 viral, 250 protists and 1500 fungal species, as well as over 5500 antibiotic resistant and virulence associated genes. The genomes included in this reference database were rigorously checked, authenticated and cleaned for most common errors, contaminating sequences, and taxonomic misclassifications and inconsistencies present in open source databases, thus offering finer levels of taxonomic classification. Taken together, this reference database enables strain-level, multi-kingdom identification of bacteria, viruses, fungi and protists in addition to antimicrobial resistance and virulence characterization of microbiome samples.

**Algorithm**: The CosmosID’s proprietary machine learning-based $k$-mer algorithm aims to achieve robust sensitivity while having the false positive rate under control thereby ensuring high detection specificity and precision. The algorithm includes a pre-computation phase for the reference database and a per-sample computation phase. Instead of organizing the reference microbial genome database based on taxonomic hierarchy, in the pre-computation phase the algorithm organizes the databases as a phylogenic tree. It then searches for hundreds of millions of variable lengths $k$-mer signatures (biomarkers) that are uniquely identified with distinct nodes, branches and leaves of the tree. Additionally, the topology of the phylogenetic tree is mapped with corresponding taxonomic hierarchy information such that this organization helps differentially detect closely related strains based on the unique biomarkers each taxonomic unit can contain. In the per-sample computation phase, the algorithm searches hundreds of millions of short sequences reads against the biomarkers sets to identify reads for which there is an exact match with a $k$-mer uniquely identified with a reference genome. To enhance detection specificity, the algorithm then uses edit distance-scoring techniques to statistically score the entire reads against the corresponding reference genome in order to verify that the read is indeed uniquely identified with that reference. The resulting search statistics are then analyzed to give fine-grained community composition up to strain-level resolution and abundance estimates of the identified taxa. An abundance score for each identified taxon is calculated based on the number of organism-specific $k$-mers and their average observed frequency, which are then normalized to average percentage of organism specific $k$-mer hits in the sample. The platform also provides a machine learning based filtering function, which filters out low-confidence taxa by pruning the phylogenetic tree based on a number of internal statistical scores devised through analyzing a large number of benchmarking *in silico* and laboratory constructed samples. Notably, CosmosID’s metagenomic analysis platform has been selected as the top performer in the strain-level microbial profiling category of the Mosaic Community Challenge 1 [3] and in the PrecisionFDA CDRH Biothreat Detection Challenge [4]. Benchmarking of this platform using a large set of datasets and as compared to other commercial metagenomic analysis tools can be found at [5, 6].

Additional periodically updated details about the CosmosID platform can be found in the following links:

1. CosmosID Cloud Bioinformatics Platform: <https://app.cosmosid.com/>
2. CosmosID Documentation: <https://app.cosmosid.com/docs/>
3. CosmosID Recent Publications: <http://www.cosmosid.com/publications/>

## Metabolomics profiling and analysis details

**Extraction, Purification and Derivatization**: From each stool samples, 10 ± 1 mg was transferred to an Eppendorf microcentrifuge tube containing the extraction solution (solvent mixture plus the internal standard 2-isopropyl malic acid). The samples were then vortexed at 1250 rpm for 30 minutes, before putting the samples in an ultrasonic bath at 30° C for 30 minutes. The samples were then centrifuged for 5 minutes at 16,000 rpm at 4° C. From the supernatant, 200 µl was removed and transferred to an Eppendorf microcentrifuge tube containing a purification mixture, and then vortexed at 1250 rpm for 5 minutes. The sample was again centrifuged at 16,000 rpm (at 4° C). Finally, 175 µl supernatant was transferred into a 2 mL glass autosampler vial and freeze-dried overnight.

To facilitate derivatization, 50 µL of the first derivatization mixture (methoxylamine in pyridine) was added to lyophilized samples and vortexed at 1200 rpm (25° C) for 90 minutes; next, 25 µL of the second derivatization mixture (N,O-Bis(trimethylsilyl)trifluoroacetamide and trimethylchlorosilane,) was added and vortexed at 1200 rpm (25° C) for 90 minutes. The derivatized metabolome was centrifuged for 5 minutes at 16,000 rpm (at 4° C) before injecting into the GC-MS.

**GC-MS analysis:** Of the derivatized solution, 1.8 µL was injected into the GC-MS system. Chromatographic separation was achieved with a 30 m 0.25 mm J&W CP-Sil 8 CB fused silica capillary GC column with 1.00 µm film thickness (Agilent, Santa Clara, CA, USA), with helium as carrier gas.

The initial oven temperature of 100° C was held for 1 min and raised to 320° C at a rate of 6° C/min, with 2.33 minutes of hold time. The gas flow was set to achieve a constant linear velocity of 39 cm/s and the split flow was set to 1:5. The mass spectrometer was operated in electron impact (70 eV) in full scan mode in the interval of 35-600 m/z with a scan velocity of 3333 amu/sec and a solvent cut time of 4.5 minute. The complete GC program duration was 40 minutes.

Samples were divided in batches of 15. Each batch was monitored with 3 quality controls: a blank injection, an injection of a mixed standard and a repeat injection of one sample (randomly selected from the samples in that batch). The blank consisted of hexane while the mixed standard was made up of 50 molecules spanning a wide range of volatilities and molecular weights (e.g., organic acids, sugars, amino acids, sterols, fatty acids, vitamins, nitrogen bases, etc.). Each batch was considered valid if the blank generated no detectable peaks, the mixed standard resulted in all peak areas being within 10% of the expected value, and the peak areas of the 100 major peaks of the repeated injection were within 15% of the original sample.

Because the resulting chromatograms were not baseline resolved for all metabolites, extracted ion chromatograms were deconvoluted using the most intense fragment for all metabolites. Peak areas were calculated for each peak that respected the integration criteria (Area>10000; Slope>100/min; Width>1 sec). These peak areas comprise the dataset for statistical analysis.

**Data pre-processing:** Gas chromatography-mass spectrometry analysis detected >250 peaks in each sample; some of these peaks were not investigated further, as they were either not consistently found in all samples, too low in concentration, or could not be confirmed as metabolites due to poor spectral quality. A total of 207 endogenous metabolites were detected consistently and identified confidently.

Chromatograms were first aligned by means of parametric time warping using the PTW package[7]. For each chromatogram, peak areas for each metabolite of interest were first normalized to the sample mass and then to the peak area of the internal standard. These values were tabulated with one sample per row and one variable (normalized metabolite area) per column. Each value was transformed by taking the natural log and then scaled by mean-centering and dividing by the standard deviation of that column (auto scaling) [8].

## Association analysis

In order to link microbial species, pathways and metabolites that were identified as significant in our analyses, we performed an association analysis using Spearman correlation to identify statistically significant correlations between every possible pair of (microbe, metabolite) and (pathway, metabolite) where microbes, pathways and metabolites are the ones identified in MaAsLin, cross-sectional and longitudinal analysis. The analysis was performed separately for significant features identified in MaAsLin, cross-sectional and longitudinal analysis and for each time point. Any significant association (p-value < 0.05) for which the absolute value of the correlation coefficient is greater than 0.01 was reported. In some cases where too many associations were identified, we used a more stringent cutoff to reduce the number of reported associations. All simulations were performed in Visual Basic by writing a custom script to calculate the Spearson correlation coefficient and the p-values.

## References

1. Hasan NA, Young BA, Minard-Smith AT, Saeed K, Li H, Heizer EM, McMillan NJ, Isom R, Abdullah AS, Bornman DM *et al*: **Microbial community profiling of human saliva using shotgun metagenomic sequencing**. *PLoS One* 2014, **9**(5):e97699.

2. Ponnusamy D, Kozlova EV, Sha J, Erova TE, Azar SR, Fitts EC, Kirtley ML, Tiner BL, Andersson JA, Grim CJ *et al*: **Cross-talk among flesh-eating Aeromonas hydrophila strains in mixed infection leading to necrotizing fasciitis**. *Proc Natl Acad Sci U S A* 2016, **113**(3):722-727.

3. **Mosaic Community Challenge** [<https://platform.mosaicbiome.com/challenges/1>]

4. **PrecisionFDA CFSAN Pathogen Detection Challenge** [<https://precision.fda.gov/challenges/3/view/results>]

5. **Benchmarking of CosmosID's metagenomic analysis platform**[<https://www.cosmosid.com/blog-in/2019/3/20/extensive-academic-study-shows-cosmosids-best-in-class-accuracy-and-unrivaled-detection-resolution>]

6. Thoendel M, Jeraldo P, Greenwood-Quaintance KE, Yao J, Chia N, Hanssen AD, Abdel MP, Patel R: **Comparison of Three Commercial Tools for Metagenomic Shotgun Sequencing Analysis**. *J Clin Microbiol* 2019.

7. Wehrens R, Bloemberg TG, Eilers PH: **Fast parametric time warping of peak lists**. *Bioinformatics* 2015, **31**(18):3063-3065.

8. van den Berg RA, Hoefsloot HC, Westerhuis JA, Smilde AK, van der Werf MJ: **Centering, scaling, and transformations: improving the biological information content of metabolomics data**. *BMC Genomics* 2006, **7**:142.
