# Supplementary material for: Multi-omics analysis reveals the influence of genetic and environmental risk factors on developing gut microbiota in infants at risk of celiac disease
Source: Microbiome. 2020 Sep 11;8:130. doi: 10.1186/s40168-020-00906-w (PMC7488762; doi:10.1186/s40168-020-00906-w)

# Pathways Abundance: Cross-Sectional Analysis: Exposed- Not Exposed

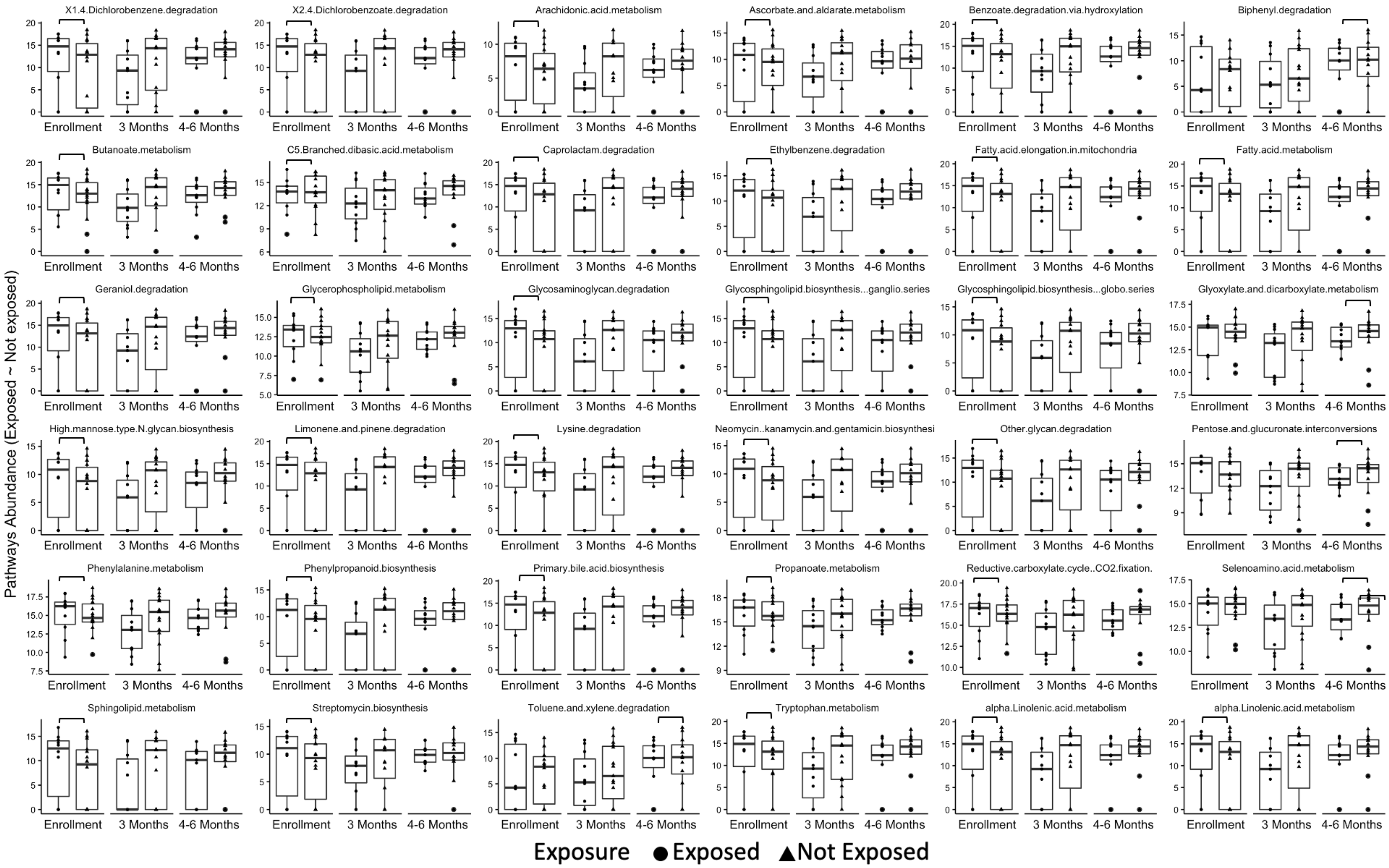

# Pathways Abundance: Longitudinal Analysis: Exposed

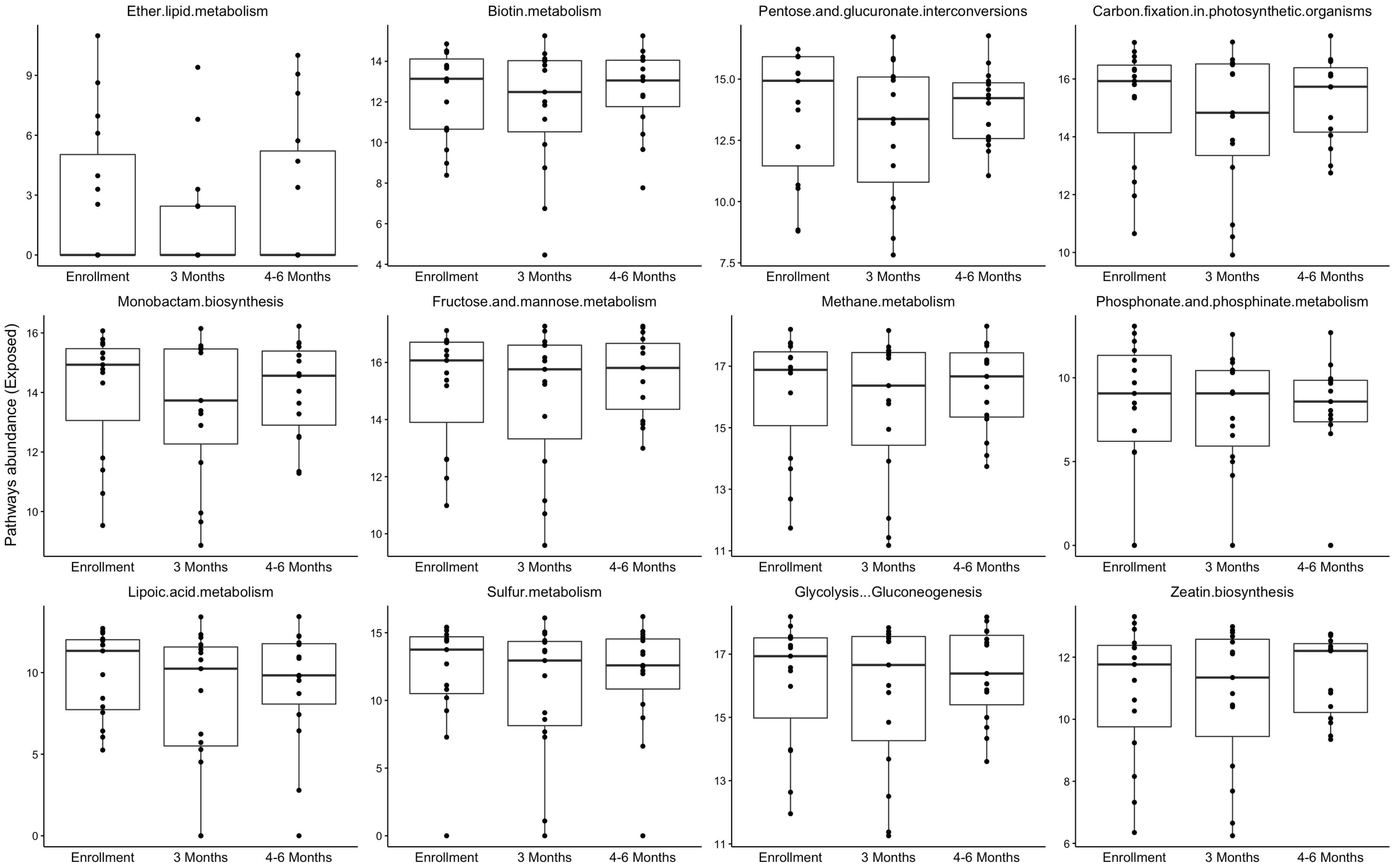

# Pathways Abundance: Longitudinal Analysis:  Not- Exposed

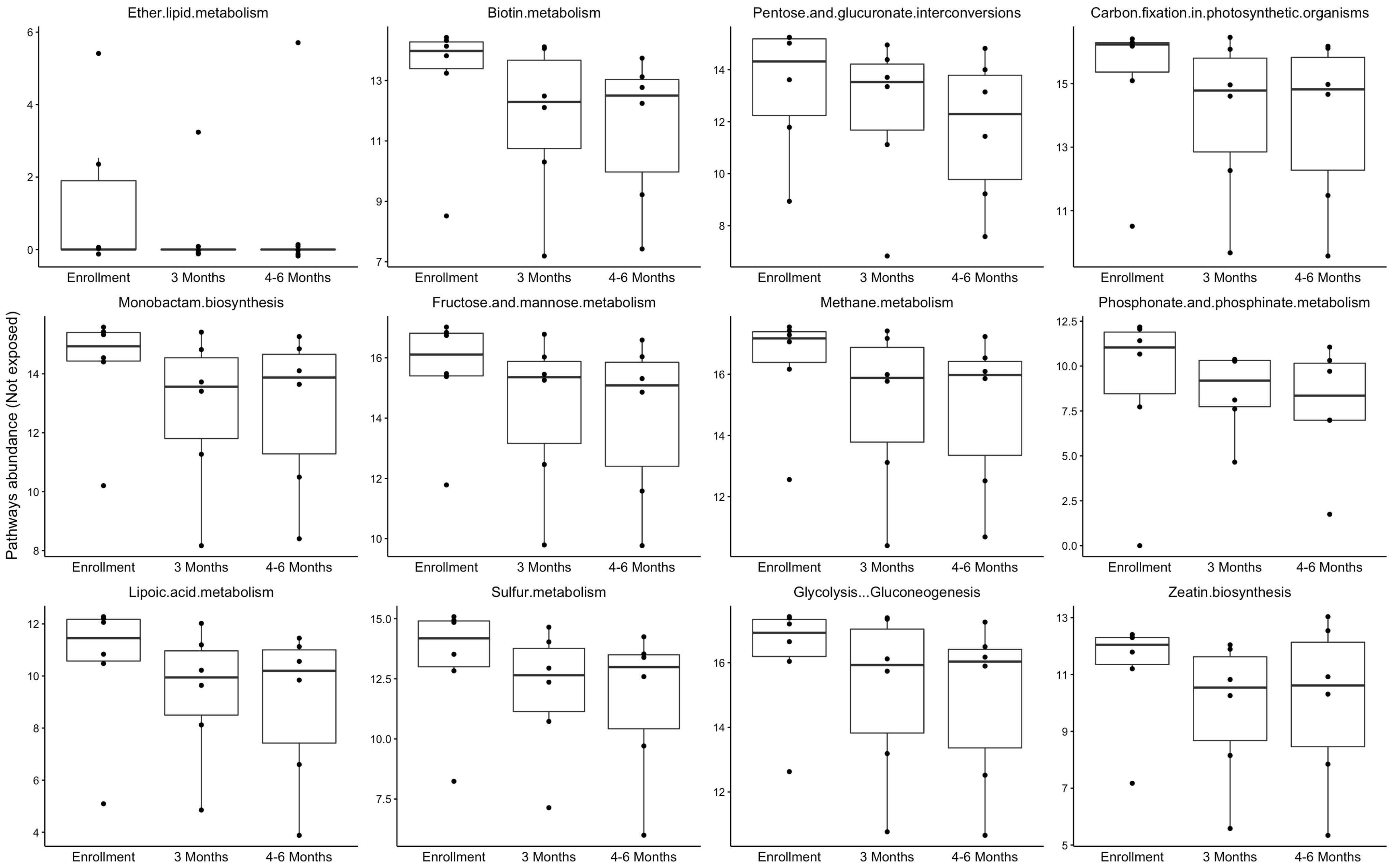

# Metabolite Abundance: Cross-Sectional Analysis: Exposed- Not Exposed

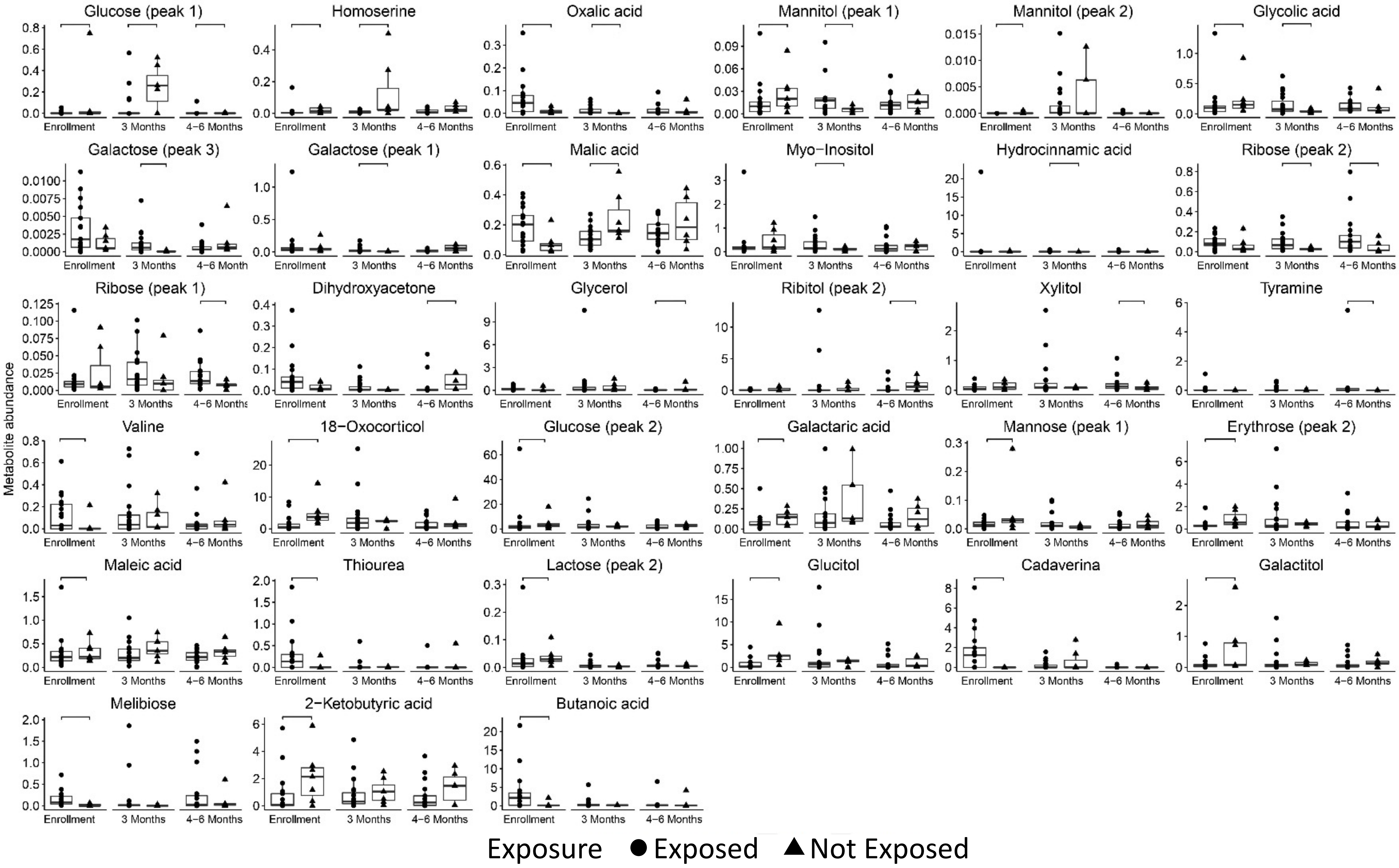

# Metabolite Abundance: Longitudinal Analysis: Exposed

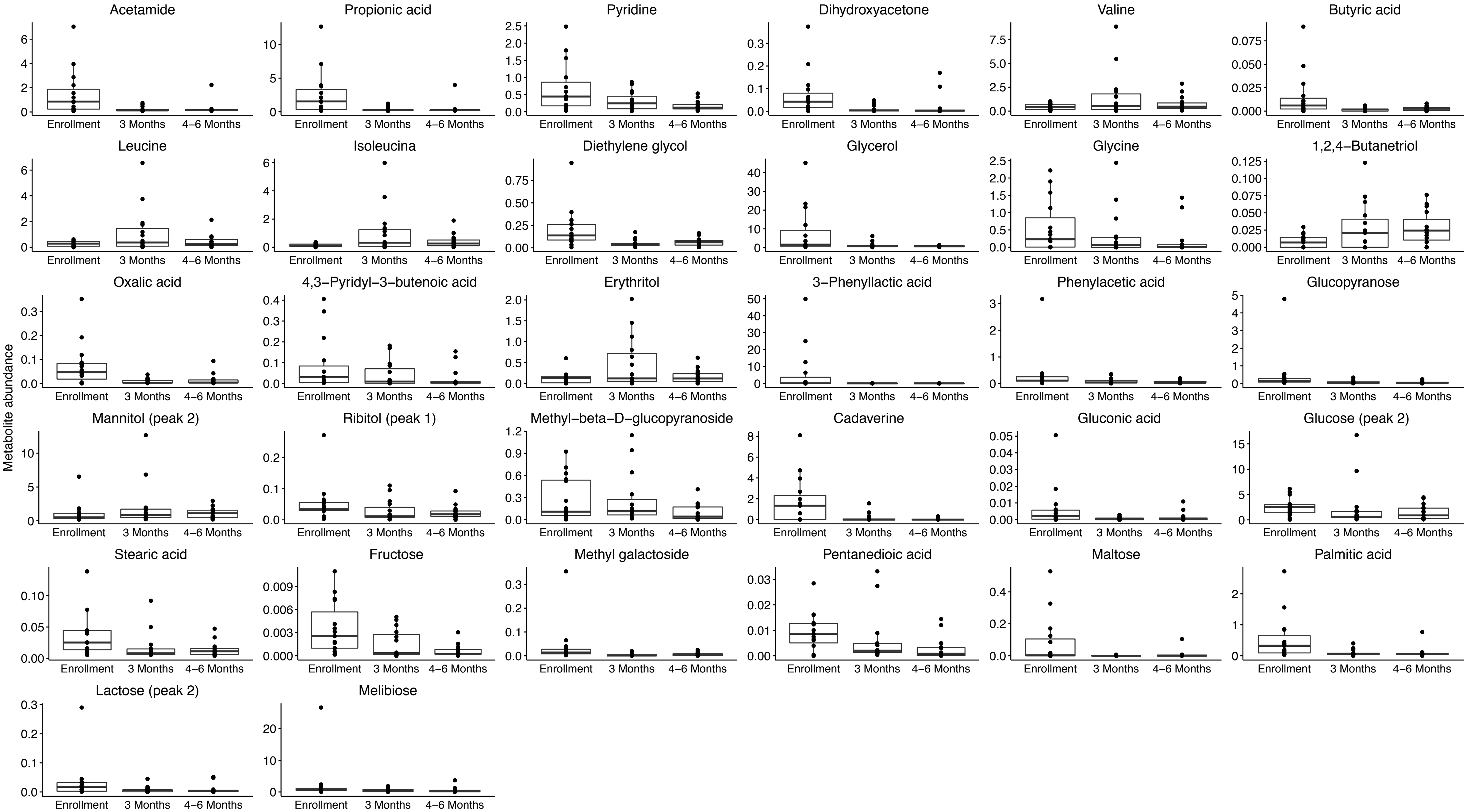

# Metabolite Abundance: Longitudinal Analysis: Not-Exposed

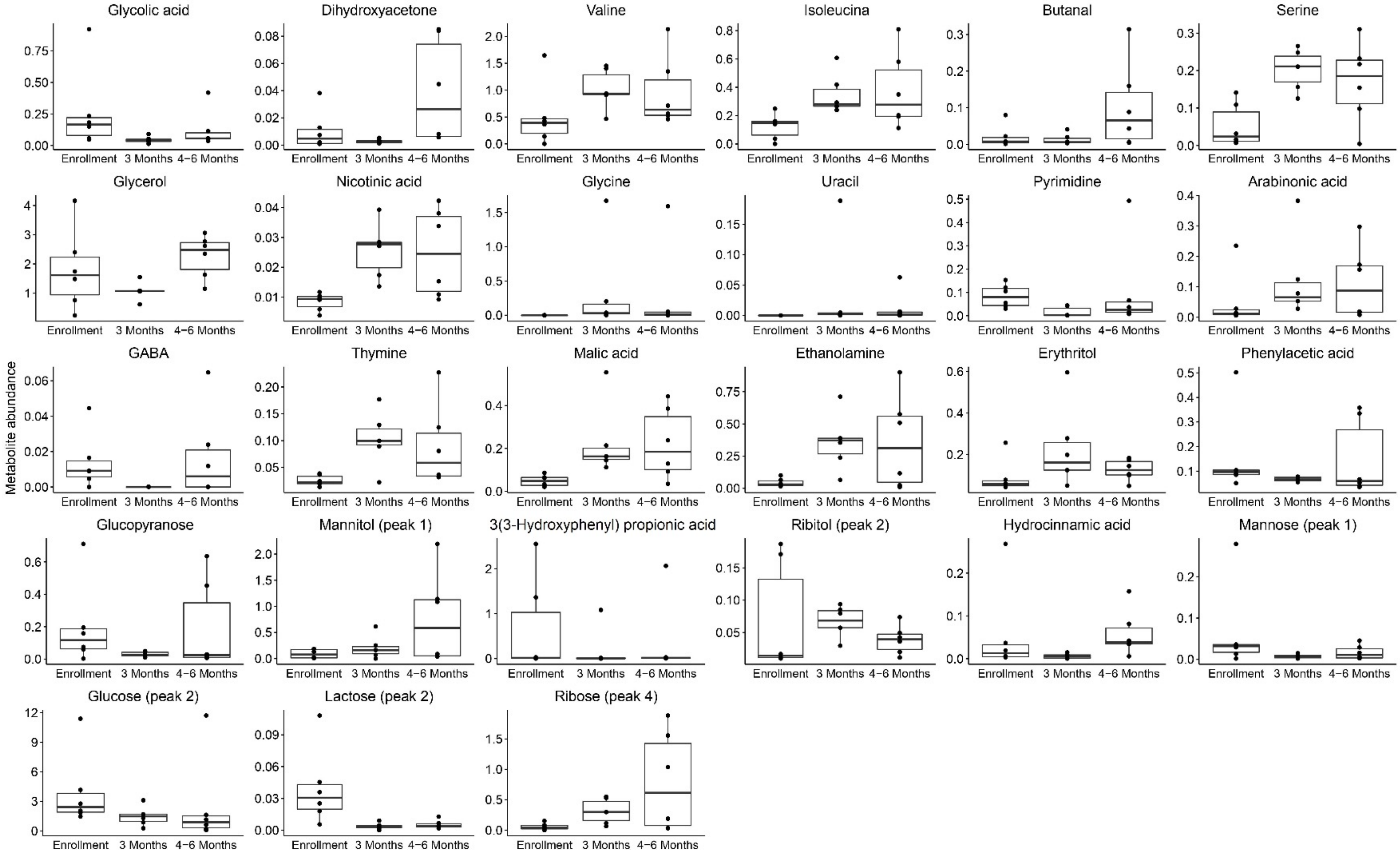

Supplement: Supplementary file 10 — Additional file 9: Boxplots for significant features in the cross-sectional and longitudinal analysis [file 40168_2020_906_MOESM9_ESM.pdf]
